# Synthesis of α-Chloroarylacetic Acid via Electrochemical Carboxylation of α,α-Dichloroarylmethane Derivatives

**DOI:** 10.3390/molecules28186704

**Published:** 2023-09-20

**Authors:** Corentin Maret, Nicolas David, David Pierrot, Eric Léonel, Vincent Levacher, Jean-François Brière, Sylvain Oudeyer

**Affiliations:** 1 Univ Rouen Normandie, INSA Rouen Normandie, CNRS, Normandie Univ, COBRA UMR 6014, INC3M FR 3038, F-76000 Rouen, France; corentin.maret@insa-rouen.fr (C.M.); nicolas.david@insa-rouen.fr (N.D.); david.pierrot@insa-rouen.fr (D.P.); vincent.levacher@insa-rouen.fr (V.L.); jean-francois.briere@insa-rouen.fr (J.-F.B.); 2 Université Paris-Est Créteil, ICMPE (UMR 7182), CNRS, UPEC, F-94320 Thiais, France; eric.leonel@cnrs.fr

**Keywords:** carboxylation reaction, electrochemical reduction, α-chlorophenylacetic acids

## Abstract

The electrocarboxylation of α,α-dichloroarylmethane derivatives in the presence of CO_2_ was achieved, providing several α-chloroarylacetic acid derivatives with modest yields but high selectivity (chlorinated vs. non-chlorinated or dicarboxylic acid products). The obtained products were then involved in several chemical transformations, underlining their potential as versatile intermediates in synthetic chemistry. A mechanism was also proposed based upon a control experiment and cyclic voltammetry (CV) study.

## 1. Introduction

**Scheme 1:**
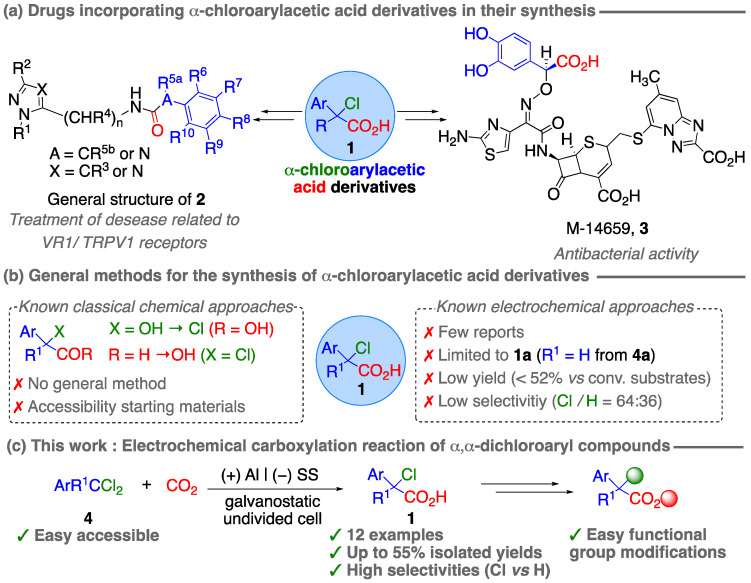
General context of the project.

α-chlorophenylacetic acid derivatives **1** are versatile synthetic intermediates involved in the synthesis of several drugs such as (1) amides or thioureas derivatives **2** used in the treatment of diseases that are at least partially mediated by vanilloid receptors 1 (VR1/TRPV1 receptors) [1] or (2) M-14659 **3**, cephalosporin, obtained by semi-synthesis, exhibiting a broad spectrum of antibacterial activity (Scheme 1a) [2]. Therefore, several chemical methods were reported to have access to α-chlorophenylacetic acids (mainly oxidation of α-chloroaryl acetaldehydes [3] or chlorination of α-hydroxyarylacetic acid [4]) but none of them proved to be general (Scheme 1b). As far as electrochemical approaches are concerned, the electrocarboxylation reaction of α-chlorobenzyl derivatives has been extensively studied [5,6,7,8,9,10,11]. Contrariwise, the electrocarboxylation of α,α-dihalobenzyl species [12] and, more particularly, α,α-dichlorobenzyl species remains more elusive. To the best of our knowledge, only two articles from the group of Silvestri (one original article and a review) deal with the electrocarboxylation reaction of α,α-dichloroarylmethane derivatives **4** [13,14]. In addition to being limited to only one example (α-chlorophenylacetic acid **1a** from α,α-dichlorophenylmethane **4a**), the experimental conditions and the electrochemical set-up are not properly described (diaphragmless cell, Al anode, Zn cathode, DMF, *n*-Bu_4_NBr (0.1 M), 100 A.m^−2^, 1.87 F.mol^−1^), no isolated yield (52% vs. converted substrate) was given for **1a** and no spectral data were provided. Moreover, the reaction proceeded with a poor selectivity (s), thus affording a 64/36 mixture of α-chlorophenylacetic **1a** acid and phenylacetic acid **5a**. Therefore, the selective reduction in one of the two benzylic geminal chlorine atoms remains a challenging task. Since the publication of these articles, electrosynthesis has undergone a rebirth, mainly during the last ten years, providing easily accessible and reproducible conditions for synthetic chemists [15,16,17,18,19,20]. We thus would like to report our contribution to the electrocarboxylation reaction of α,α-dichloroarylmethane **4** derivatives in order to provide an efficient and selective synthesis of α-chloroarylacetic acid derivatives **1** (Scheme 1c).

## 2. Results

### 2.1. Influence of Reaction Parameters

In order to investigate this transformation, we first turned our attention to the simple commercially available α,α-dichlorodiphenylmethane **4b** as a substrate in order to study the main reaction parameters (For a complete study of the reaction parameters, see Supporting Information). For the purpose of clarity, we have used the selectivity value which consists of the percentage obtained by dividing the NMR yield of the desired α-chlorophenylacetic acid methyl ester **6b** (obtained after an esterification step with trimethylsilyl diazomethane) by the sum of the NMR yields of **6b** and the over-reduced product **7b**. Starting from the aluminum anode as reported by Silvestri et al. [13] but in acetonitrile as a solvent instead of DMF, we have obtained a 66/34 mixture of α-chlorodiphenylacetic methyl ester **6b** and the over-reduced diphenylacetic methyl ester **7b** (s = 66%) in low (23% and 12%) NMR yields, respectively (Table 1, entry 1). As we were aware of the crucial role of the consumable anode in this type of reaction [21,22,23,24], we then examined the nature of this metallic electrode (Table 1, entries 2–4). Other metals such Co, Zn or Mg provided lower NMR yields, along with a significant amount of diphenylacetic methyl ester **7b**. Whereas in THF no reaction occurred (Table 1, entry 5), other aprotic polar solvents such as DMF and DMA (Table 1, entries 6 and 7) allow the reaction to proceed in 17% and 42% NMR yields, respectively, of **6b**, along with non-negligible amounts of **7b** (11% and 20% NMR yields, respectively). DMA was thus chosen as the solvent to study the influence of the cathode on the reaction performances (Table 1, entries 8–11). Among all cathodes tested, only stainless steel (SS) improved the NMR yield of **6b** to 54%, while reducing the NMR yield of **7b** to only 5% (s = 91%). As long as the charge is concerned, a 1.8 F/mol charge resulted in the complete selectivity of **6b** (s > 99%, only traces of **7b**), but at the expense of a lower, 33%, NMR yield (Table 1, entry 12), whereas a 3.2 F/mol charge provided a higher amount of **7b** (11% NMR yield) without improving the NMR yield of **6b** (54%, Table 1, entry 13). Regarding the intensity of the electrolysis, higher or lower intensities (10 or 30 mA instead of 20 mA) did not improve the NMR yield of **6b** (39% and 50%, respectively, Table 1, entries 14–15). Finally, the reduction in the concentration of **4b** from 83 mM to 35 mM had a positive impact on both the NMR yield of **6b** (55%) and the selectivity, delivering only a 5% NMR yield of **7b** (s > 92%). Under these conditions, a Faraday efficiency of 36.2% was calculated (see Supplementary Materials for calculations detail). In conclusion, after a careful study of the reaction parameters, we have found that the nature of the cathode, the solvent and the concentration has a major influence on the reaction, improving the results obtained by Silvestri [13] for compound **1a** in terms of the yield and selectivity.

### 2.2. Scope and Limitations

**Scheme 2:**
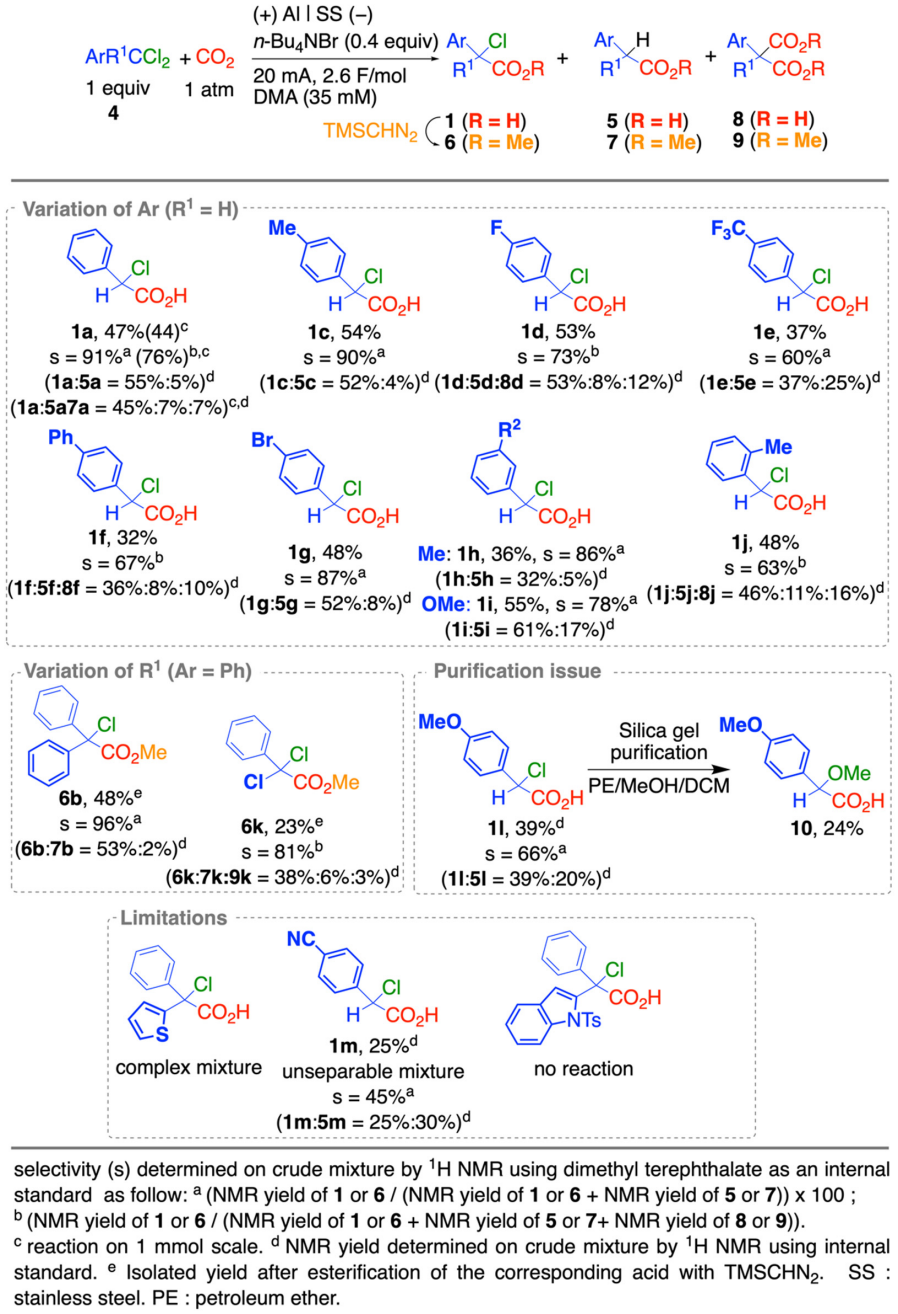
Scope and limitations of the electrochemical carboxylation of α,α-dichloroarylmethane derivatives **4**.

With these optimal conditions in hand, we then moved to the study of the scope and limitations of our methodology. Since only a few α,α-dichloroarylmethane derivatives **4** are commercially available, we put some efforts in their synthesis. By using conditions reported by Hegarty et al. starting from commercially available aromatic aldehydes **8** (PCl_5_, toluene, rt; see Section 3 below and Supplementary Materials for more details) [25], we were able to obtain several α,α-dichloroarylmethane derivatives **4**. Next, the scope and limitations of the electrochemical carboxylation reaction were studied (Scheme 2).

Generally speaking, even if a complete conversion was observed, modest yields of around 50% were obtained, but a high selectivity (Cl (**1** or **6**) vs. H (**5** or **7**) and/or dicarboxylated product (**8** or **9**)) were observed (s = 60–96%). Regarding the modulation of the Ar part, electron-withdrawing or -donating groups can be introduced, with electro-donating groups providing higher isolated yields (**1e**, 37% vs. **1i**, 55%) and selectivities (**1e**, s = 60% vs. **1i**, s = 78%). Fluoride and bromine at the *para* position are also well-tolerated (**1d**, 53% and **1g**, 48%, respectively). The introduction of a methyl substituent at the *para* or *ortho* position afforded the desired product in acceptable yields (**1c**, 54% and **1j**, 48%), whereas a methyl at the *meta* position resulted in a drop of the yield to 36% for compound **1h**. As far as the methyl substituent is concerned, an *ortho* substitution gave a lower selectivity than a *meta* and *para* substitution (s = 63% for **1j** vs. s = 90 and 86% for **1c** and **1h,** respectively). The phenyl group at the para position provided the desired product in a low yield and modest selectivity (**1f**, 32%, s = 67%). It is worthy of note that during the silica gel purification of **1l**, the chlorine atom was substituted by a methoxy group due to the presence of methanol in the mobile phase, affording compound **10** in a 24% isolated yield. We have also tried to vary the R^1^ substituent. Accordingly, a phenyl can be introduced, affording a decent 48% yield and excellent selectivity (s = 96%) for compound **6b**. Starting from α,α,α-trichlorotoluene, methyl ester **6k** bearing a *gem*-dichloro group was successfully obtained in a modest 23% yield but a good level of selectivity (s = 81%). However, some products remain problematic with otherwise improved methodology. In the case of thiophene derivatives, a complex mixture was obtained, whereas the formation of the product with an *N*-Ts indole moiety was not observed. Finally, compound **1m** was formed but as an inseparable mixture with **5m** (25% NMR yield, s = 45%).

### 2.3. Versatile Transformations of the α-Chlorophenylacetic Acid **1a**

**Scheme 3:**
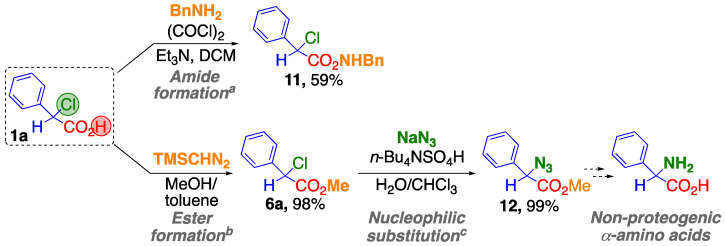
Chemical transformations on α-chlorophenylacetic acid **1a**. ^a^ (1) (COCl)_2_ (1.1 equiv), DMF (5 drops), CH_2_Cl_2_; (2) BnNH_2_ (1.3 equiv), Et_3_N (1.3 equiv), CH_2_Cl_2_. ^b^ TMSCHN_2_ (2.5 equiv), toluene/MeOH (7:3 *v*/*v*), 0 °C, rt. ^c^ NaN_3_ (2 equiv), *n*-Bu_4_NHSO_4_ (0.15 equiv), H_2_O/CHCl_3_ (1:1 *v*/*v*).

In order to demonstrate the synthetic utility of the *α*-chloroarylacetic acids, we have turned our attention to several chemical transformations (Scheme 3).

First, the acid moiety was submitted to an amidation reaction via the corresponding acid chloride in the presence of benzyl amide, affording the amide derivatives **11** in 59%. An esterification was also possible by using trimethylsilyldazomethane in a mixture of toluene and methanol to give the chlorinated ester **6a** in almost quantitative yields. More interestingly, the chlorine atom of **6a** can be submitted to nucleophilic substitution in the presence of sodium azide under liquid/liquid phase-transfer conditions, leading to **12** in an excellent yield. The product **12** is of particular interest as it paves the way to the access of non-proteogenic α-amino acid derivatives [26,27].

### 2.4. Mechanistic Considerations

**Scheme 4:**
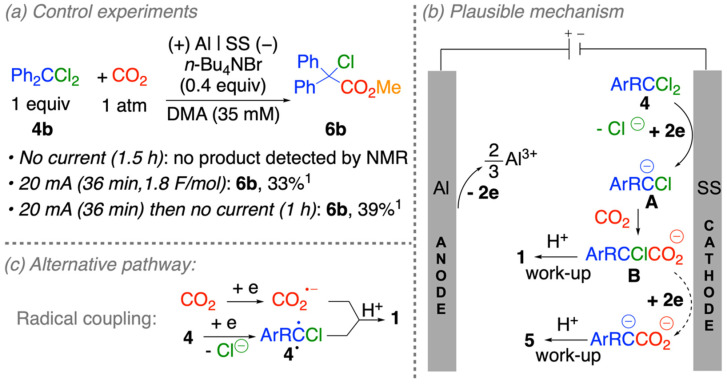
Mechanistic considerations: (**a**) control experiments; (**b**) plausible mechanism; (**c**) alternative pathway. ^1 1^H NMR yield determined on the crude mixture by using dimethyl terephthalate as an internal standard.

Finally, control experiments and CV analyses were performed to gain insight into the mechanism (Scheme 4). We first checked whether the reaction proceeded exclusively under electrochemical conditions by performing the reaction without a current for 1.5 h. No product was detected by an NMR analysis of the crude mixture (Scheme 4a). Then, the reaction was performed at 20 mA for 36 min (1.8 F/mol) and, then, the mixture was stirred for one additional hour without a current. The result was almost identical to that obtained when the reaction was stopped after the 1.8 F/mol electrolysis (see also Table 1, entry 12), indicating that the presence of Al^3+^ presumably formed at the anode during electrolysis is not involved in the reaction.

According to previous reports [13,14] and our own study (see Supplementary Materials for CV experiments) ((a) The reduction potential of PhCHCl_2_ in DMA (E_red_ = −2.2 V/SCE) was found to be similar to previously reported value in DMF, see ref [28] and supporting information. (b) Our attempts to determine the reduction potential of CO_2_ in DMA failed (see supporting information). (c) Our attempts to clarify the exact role of the Al^3+^ by CV have not been conclusive (see Supporting Information)), we have proposed a plausible mechanism (Scheme 4b). First, the α,α-dichloroarylmethane derivatives PhRCCl_2_ **4** would be reduced at the cathode (E_red_ = −2.2 V/SCE for R = H and E_red_ = −1.8 V/SCE for R = Ph in DMF) [28] thanks to a two electron process to provide the corresponding α-chloro anion **A** which then would add to CO_2_ (E_red_ = −2.2 V/SCE in DMF) [29] to give the α-chlorophenylacetic carboxylate **B** flanked by an aluminum counter-ion. Simultaneously, the aluminum anode plate would be oxidized to afford the corresponding aluminum (III) cation in solution. This aluminum counter-ion is expected to stabilize the carboxylate anion generated during the electrolysis which is generally unstable under the electrochemical conditions [7] and also to avoid its possible transformation into mandelic acid [13]. Finally, the expected product **1** would be obtained after aqueous work-up. The by-product **5** could be obtained by the further reduction in **B** at the cathode followed by aqueous work-up. Nevertheless, due the close proximity between the reduction potential of CO_2_ and that of some of the α,α-dichloroarylmethane derivatives **4**, a mechanism first involving the reduction in CO_2_ to form the corresponding radical anion followed by the radical coupling with **4^•^** (resulting from the one electron reduction in **4** at the cathode) cannot totally be ruled out (Scheme 4c).

## 3. Materials and Methods

### 3.1. General Information

Reactions were performed using oven dried glassware under an inert atmosphere of nitrogen. Unless otherwise noted, all reagent-grade chemicals and solvents were obtained from commercial suppliers and were used as received. Toluene was dried over an MBRAUN MB SPS-800 apparatus (MBRAUN inertgas-system gmbh, Garching, Germany). Reactions were monitored by thin-layer chromatography with silica gel 60 F254 pre-coated aluminum plates (0.25 mm). Visualization was performed under UV light, phosphomolybdic acid or KMnO_4_ oxidation. Chromatographic purification of compounds was achieved with 60 silica gel (40–63 μm). Melting points were measured on a WME Köfler hot-stage (Stuart SMP3) and are uncorrected (Barloworld Scientific France SAS, Nemours, France). Infrared spectra (IR) were recorded on a PerkinElmer Spectrum 100 Series FT-IR spectrometer. Liquids and solids were applied on the Single Reflection Attenuated Total Reflectance (ATR) Accessories (PerkinElmer, Wellesley, MA, USA). Data are reported in cm^−1^. ^1^H Spectra (300 MHz) and ^13^C NMR spectra (75 MHz) were recorded on a Bruker Advance 300 (Bruker, Billerica, MA, USA). Processing and analysis of the spectra were performed with the Topspin 3.6 software (Bruker, Billerica, MA, USA) on a PC workstation. Data appear in the following order: chemical shifts in ppm which were referenced to the internal solvent signal, number of protons, multiplicity (s, singlet; d, doublet; t, triplet; q, quadruplet; dd, doublet of doublet, ddd, doublet of doublet of doublet, dt, doublet of triplet; ddt, doublet of doublet of triplet, td, triplet of doublet; tdd, triplet of doublet of doublet; m, multiplet, ABq, AB system) and coupling constant J in Hertz. Accurate mass measurements (HRMS) were performed by the Mass Spectrometry Laboratory of the University of Rouen and were recorded with a Waters LCP 1er XR spectrometer (Waters, Milford, CT, USA). The electrosynthesis were carried out by means of IKAElectraSynth^®^ 2.0 apparatus. Electrodes were all purchased from IKA® (IKA, Staufen, Germany). Cyclic Voltammetry (CV) measurements were carried out with an OrigaFlex® potentiostat/galvanostat by means of three electrodes (Origalys, Rilleux-la-Pape, France).

### 3.2. General Procedure for the Synthesis of α,α-Dichloro Benzyl Derivatives **4** [25]

To a dry 10 mL flask containing PCl_5_ (1.3 equiv), dry toluene (5 mL) was added the corresponding aldehyde (1 equiv) and the mixture was then stirred for 16 h. After completion, the reaction was diluted with 20 mL EtOAc and washed two times with 20 mL saturated NaHCO_3_ aqueous solution, then once with 10 mL brine. The organic layer was dried over MgSO_4_, filtered, and concentrated at 40 °C under 80 mbar pressure. The residue was purified by silica gel column chromatography (100/0 EP:EtOAc to 95/5) to obtain the corresponding α,α-dichloro aryl compounds **4**. As mentioned by the authors and due their high reactivity, products **4** were generally used rapidly after their synthesis; **4a**, **4j** and **4k** were commercially available and were used as received.

*α,α-dichloro-4-methyltoluene* **4c**: Following the general procedure with 4-methylbenzaldehyde (300 mg, 2.5 mmol), the title compound **4c** was obtained as a white solid (367 mg, 84%). 1H NMR (300 MHz, CDCl3) δ 7.49–7.46 (m, 2H, C*H*_Ar_), 7.23–7.20 (m, 2H, C*H*_Ar_), 6.70 (s, 1H, C*H*), 2.39 (s, 3H, C*H*_3_). 13C NMR (75 MHz, CDCl3) δ 140.3 (*C*_Ar_), 137.8 (*C*_Ar_), 129.6 (*C*H_Ar_), 126.1 (*C*H_Ar_), 72.0 (*C*H), 21.4 (*C*H_3_). The spectral data were in agreement with those previously reported [30].

### 3.3. General Procedure A for the Synthesis of α-Chloroarylacetic Acid Derivatives **1**

In a dry electrochemical cell (10 mL vial from IKA) containing *n*-Bu_4_NBr (33 mg, 0.1 mmol) DMA (7 mL) was added and α,α-dichloro aryl derivates **4** (0.25 mmol) under nitrogen. The electrodes (aluminum electrode as anode and stainless steel as cathode) were installed, and carbon dioxide was bubbled through the solution for 5 min using a CO_2_ balloon. The reaction was carried out at room temperature for 48 min, with a constant current of 20 mA and a charge of 3.2 F.mol^−1^. After completion, the electrodes were rinsed subsequently with EtOAc, HCl 1M and then water. The reaction mixture was diluted with 15 mL of EtOAc and washed with 15 mL of HCl 0.5 M. The aqueous phase was washed a second time with 10 mL of EtOAc and then the combined organic extract was washed three times with 10 mL of HCl 0.5 M. The organic layer was dried over MgSO_4_, filtered and concentrated under reduced pressure. The NMR yield was measured by ^1^H NMR of crude by means of dimethyl terephthalate as internal standard (0.25 equiv, 12.2 mg). The residue was purified by silica gel column chromatography (5/25/70 MeOH:CH_2_Cl_2_:PE, then 1/20/79 HCOOH:Et_2_O:PE) to obtain the corresponding α-chloroarylacetic acid **1**.

#### 1 mmol Procedure

In a dry electrochemical cell (20 mL vial from IKA) containing *n*-Bu_4_NBr (110 mg, 0.34 mmol) DMA was added (16 mL) and α,α-dichlorotoluene **4a** (126 mL, 1.00 mmol) under nitrogen. The electrodes (aluminum electrode as anode and stainless steel as cathode) were installed, and carbon dioxide was bubbled through the solution for 5 min using a CO_2_ balloon. The reaction was carried out at room temperature for 48 min, with a constant current of 20 mA and a charge of 3.2 F.mol^−1^. After completion, the electrodes were rinsed subsequently with EtOAc, HCl 1M and then water. The reaction mixture was diluted with 30 mL of EtOAc and washed with 30 mL of HCl 0.5 M. The aqueous phase was washed a second time with 20 mL of EtOAc and then the combined organic extract was washed three times with 20 mL of HCl 0.5 M. The organic layer was dried over MgSO_4_, filtered and concentrated under reduced pressure. The residue was purified by silica gel column chromatography (5/25/70 MeOH:CH_2_Cl_2_:PE, then 1/20/79 HCOOH:Et_2_O:PE) to obtain the corresponding 2-chloro-2-phenylacetic acid **1a** as a white solid (75 mg, 44%).

*2-chloro-2-(4-methylphenyl)acetic acid* **1c**: Following the general procedure A with **4c** (46 mg, 0.26 mmol), the title compound **1c** was obtained as a white solid (26 mg, 54%). mp = 89–90 °C. 1H NMR (300 MHz, CDCl3) δ 10.55 (bs, 1H, CO_2_*H*), 7.40–7.38 (d, J = 8.2 Hz, 2H, C*H*_Ar_), 7.21–7.19 (d, J = 8.2 Hz, 2H, C*H*_Ar_), 5.35 (s, 1H, C*H*), 2.36 (s, 3H, C*H*_3_). 13C NMR (75 MHz, CDCl3) δ 174.3 (*C*O_2_H), 139.8 (*C*_Ar_), 132.0 (*C*_Ar_), 129.7 (*C*H_Ar_), 127.9 (*C*H_Ar_), 58.6 (*C*H), 21.3 (*C*H_3_). IR (neat) n 2323, 1721, 1512, 1412, 1286, 1201, 912, 804, 787 cm^−1^. HRMS (TOF-ESI^−^): calcd for C_9_H_8_Cl_2_O_2_ [(M-H)^−^]: 183.0218; found: 183.0206.

### 3.4. Synthesis of Methyl Methyl 2-Chloro-2-phenylacetate **6a**

The 2-chloro-2-phenylacetic acid **1a** (1.00 g, 5.86 mmol) was then dissolved in toluene/methanol mixture (10 mL, 7:3 *v*/*v*) and cooled to 0 °C. TMS diazomethane (2M in hexanes, 2.17 mL, 14.7 mmol) was then added until a yellow coloration persisted in the solution. The solution was then stirred for 30 min at 0 °C and then 30 min at r.t. The solution was then quenched with acetic acid (0.1 mL), MeOH was evaporated and the residual toluene solution was diluted with ethyl acetate. The organic phase was washed with HCl 1M and saturated with aqueous NaHCO_3_ solution and then water. The organic layer was dried over MgSO_4_, filtered and concentrated under reduced pressure. Methyl 2-chloro-2-phenylacetate derivatives **6** (1.06 g, 5.74 mmol, 98%) was obtained as a transparent oil. 1H NMR (300 MHz, CDCl3) δ 7.51–7.48 (m, 2H, C*H*_Ar_), 7.40–7.36 (m, 3H, C*H*_Ar_), 5.37 (s, 1H, C*H*), 3.78 (s, 3H, C*H*_3_). Spectroscopic data were in agreement with those previously reported [31].

### 3.5. General Procedure B for the Synthesis of Methyl 2-Chloro-2-phenylacetate Derivatives **6b,k**

In a dry electrochemical cell (10 mL vial from IKA) containing *n*-Bu_4_NBr (33 mg, 0.1 mmol), DMA was added (7 mL) and α,α-dichloro aryl derivates **4** (0.25 mmol) under nitrogen. The electrodes (aluminum electrode as anode and stainless steel as cathode) were installed, and carbon dioxide was bubbled through the solution for 5 min using a CO_2_ balloon. The reaction was carried out at room temperature for 48 min, with a constant current of 20 mA and a charge of 3.2 F.mol^−1^. After completion, the electrodes were rinsed subsequently with EtOAc, HCl 1M and then water. The reaction mixture was diluted with 15 mL of EtOAc and washed with 15 mL of HCl 0.5 M. The aqueous phase was washed a second time with 10 mL of EtOAc and then the combined organic extract was washed three times with 10 mL of HCl 0.5 M. The organic layer was dried over MgSO_4_, filtered and concentrated under reduced pressure. The crude mixture was then dissolved in toluene/methanol mixture (7:3 *v*/*v*) and cooled to 0 °C. TMS diazomethane was then added until a yellow coloration persisted in the solution. The solution was then stirred for 15 min at 0 °C and then 15 min at r.t. The solution was then quenched with acetic acid and extracted with ethyl acetate and washed with saturated aqueous NaHCO_3_ solution, HCl 1M and then water. The NMR yield was measured by ^1^H NMR of crude by means of dimethyl terephthalate as internal standard (0.25 equiv, 12.2 mg). The residue was purified by silica gel column chromatography (5/25/70 MeOH:CH_2_Cl_2_:PE, then 1/20/79 HCOOH:Et_2_O:PE) to obtain the corresponding methyl 2-chloro-2-phenylacetate derivatives **6**.

*Methyl 2-chloro-2,2-diphenylacetate* **6b**: Following the general procedure B with **4b** (59 mg, 0.25 mmol), the title compound **6b** was obtained as colorless oil (31 mg, 48%). 1H NMR (300 MHz, CDCl3) δ 7.42–7.26 (m, 10H, C*H*_Ar_), 3.78 (s, 3H, C*H*_3_). Spectroscopic data were in agreement with those previously reported [32].

### 3.6. Procedure for the Synthesis of 2-Methoxy-2-(4-methoxyphenyl)acetic Acid **10**

Compound **10** was obtained after a slight modification of the general procedure A. The crude product was dissolved in MeOH, SiO_2_ was added and the resulting mixture was stirred overnight at room temperature. After filtration and evaporation of MeOH, the resulting mixture was purified according to general procedure A. Starting from **1l** (48 mg, 0.25 mmol), the title compound **10** was obtained as a white solid (12 mg, 24%). 1H NMR (300 MHz, CDCl3) δ 7.37–7.32 (m, 2H, C*H*_Ar_), 6.93–6.88 (m, 2H, C*H*_Ar_), 4.73 (s, 1H, C*H*), 3.81 (s, 3H, C*H*_3_), 3.39 (s, 3H, C*H*_3_). Spectroscopic data were in agreement with those previously reported [33].

### 3.7. Procedure for the Synthesis of N-Benzyl-2-chloro-2-phenylacetamide **11**

Product **11** was synthesized following the procedure reported in the literature [34], as follows: oxalyl chloride (273 mL, 3.22 mmol, 1.1 equiv) and DMF (5 drops) were added to a 0 °C solution of 2-chloro-2-phenylacetic acid **1a** (500 mg, 2.93 mmol, 1 equiv) in anhydrous CH_2_Cl_2_ (5 mL) in an oven-dried schlenk tube. The reaction mixture was allowed to warm to room temperature, stirring for 1 h. The solution was then transferred by cannula to a solution of benzyl amine (417 mL, 3.81 mmol, 1.3 equiv) and triethylamine (514 mL, 3.81 mmol, 1.3 equiv) in anhydrous CH_2_Cl_2_ (5 mL) at 0 °C. The suspension was stirred for 4 h and then the reaction was quenched by the addition of HCl (1 M). The reaction mixture was extracted with CH_2_Cl_2_ (25 mL × 3) and the combined organic layers were washed with brine, dried over Na_2_SO_4_ and concentrated. The residue was purified by flash chromatography (hexane/dichloromethane = 2/1) which furnished the desired product (450 mg, 59%) as white solid. ^1^H NMR (300 MHz, CDCl_3_) *δ δ* 7.46–7.27 (m, 10H, C*H*_Ar_), 7,00 (br s, 1H, N*H*) 5.43 (s, 1H, C*H*), 4.52 (d, *^3^J* = 5.8 Hz, 2H, C*H*_2_). Spectroscopic data were in agreement with those previously reported [35].

### 3.8. Procedure for the Synthesis of Methyl 2-Azido-2-phenylacetate **12**

The title product was synthesized according to a reported procedure [36], as follows: Methyl 2-chloro-2-phenylacetate **6a** (194 mg, 1 mmol) was mixed with NaN_3_ (130 mg, 2 mmol) and tetrabutylammonium hydrogensulfate (50 mg, 0.15 equiv) in a mixture of water (1 mL) and chloroform (1 mL). The reaction was allowed to stir at room temperature for 24 h in the dark, after which time the aqueous layer was removed and the organic layer was washed with water (3 × 10 mL) and dried over sodium sulfate. The solvent was removed carefully by rotary evaporation under reduced pressure without heating. The title compound **12** was obtained as a colorless oil (191 mg, 99%). 1H NMR (300 MHz, CDCl3) δ 7.41–7.37 (m, 5H, C*H*_Ar_), 4.99 (s, 1H, C*H*), 3.76 (s, 3H, C*H*_3_). Spectroscopic data were in agreement with those previously reported [37].

## 4. Conclusions

In conclusion, we have reported on an efficient and selective electrocarboxylation reaction of α,α-dichlorobenzyl compounds **4** to afford the corresponding α-chloroaryl acetic acid derivatives **1** with moderate isolated yields but high selectivity (the other chlorine atom is not affected by the reduction process). Several α-chloroaryl acetic acid derivatives with different substitution patterns, both on the methylene carbon at the benzylic position and the aromatic ring, were obtained. Moreover, we have demonstrated the versatility of the obtained products by modulating either the carboxylic acid part (ester or amide formation) or the chlorine atom (introduction of an azide moiety *en route* to the synthesis of non-proteogenic α-amino acids). Finally, thanks to control experiments and a cyclic voltammetry study, a plausible mechanism was proposed.

## Figures and Tables

**Table 1:** Optimization of the reaction conditions.

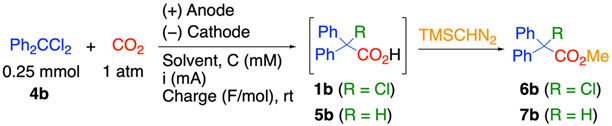							
Entry	Anode/Cathode	Solvent	Conc. (mM)	Charge (F/mol)	i (mA)	NMR Yield 6b/7b (%) ^1^	Selectivity, s (%) ^2^
1	Al/Ni_Foam_	ACN	83	2.6	20	23/12	66
2	**Co**/Ni_Foam_	ACN	83	2.6	20	13/11	54
3	**Zn**/Ni_Foam_	ACN	83	2.6	20	4/7	36
4	**Mg**/Ni_Foam_	ACN	83	2.6	20	6/17	26
5	**Al**/Ni_Foam_	**THF**	83	2.6	20	nr/-	-
6	Al/Ni_Foam_	**DMF**	83	2.6	20	17/11	61
7	Al/Ni_Foam_	**DMA**	83	2.6	20	42/20	68
8	Al/**Glassy C**	DMA	83	2.6	20	32/26	55
9	Al/**Graphite**	DMA	83	2.6	20	25/24	51
10	Al/**Ni**	DMA	83	2.6	20	36/10	78
11	Al/**SS**	DMA	83	2.6	20	54/5	91
12	Al/SS	DMA	83	**1.8**	20	33/traces	>99
13	Al/SS	DMA	83	**3.2**	20	54/11	83
14	Al/SS	DMA	83	2.6	**10**	39/6	87
15	Al/SS	DMA	83	2.6	**30**	50/8	86
16	Al/SS	DMA	**125**	2.6	20	29/7	81
17	Al/SS	DMA	**35**	2.6	20	55(48) ^3^/<5	>92

^1^ NMR yields were determined on ^1^H NMR spectra of the crude product (after an esterification step with TMS diazomethane) in the presence of dimethyl terephthalate as an internal standard. ^2^ Selectivity (s) determined on crude mixture by ^1^H NMR using an internal standard, as follows: (NMR yield of **6b**/(NMR yield of **6b** + NMR yield **7b**)) × 100. ^3^ Isolated yield. SS: stainless steel. nr: no reaction. Bold text indicates the parameter which has been modified.

## Data Availability

Data is contained within the article or Supplementary Materials.

## Supplementary Materials

The following supporting information can be downloaded at: https://www.mdpi.com/article/10.3390/molecules28186704/s1; complete study of reaction parameters, cyclic voltammetry analyses, faraday efficiency calculations, general procedure for the synthesis of all compounds, spectral data and copies of NMR for compounds **1a**,**c–j**,**l–m**, **4c–j**,**l–m**, **6a**,**b**,**k**, **10**, **11**, **12**. Refs. [38,39,40,41] are cited in Supplementary Materials.
