# Supplementary material for: Synthesis of α-Chloroarylacetic Acid via Electrochemical Carboxylation of α,α-Dichloroarylmethane Derivatives
Source: Molecules. 2023 Sep 20;28(18):6704. doi: 10.3390/molecules28186704 (PMC10537669; doi:10.3390/molecules28186704)
Supplement: Supplementary file 1 [file molecules-28-06704-s001.zip › molecules-2614554-supplementary.pdf]

Synthesis of α-Chloroarylacetic Acid *via* Electrochemical Carboxylation of α,α-Dichloroarylmethane Derivatives

Corentin Maret [1], Nicolas David [1], David Pierrot [1], Eric Léonel [2], Vincent Levacher [1], Jean-François Brière [1] and Sylvain Oudeyer [1,*]

[1] Univ Rouen Normandie, INSA Rouen Normandie, CNRS, Normandie Univ, COBRA UMR 6014, INC3M FR 3038, F-76000, Rouen, France; corentin.maret@insa-rouen.fr (C.M.); nicolas.david@insa-rouen.fr (N.D.); david.pierrot@insa-rouen.fr (D.P.); vincent.levacher@insa-rouen.fr (V.L.); jean-francois.briere@insa-rouen.fr (J.-F.B.)
[2] Université Paris-Est Créteil, ICMPE (UMR 7182), CNRS, UPEC, F-94320 Thiais, France; eric.leonel@cnrs.fr
* Correspondence: sylvain.oudeyer@univ-rouen.fr

I. GENERAL INFORMATION 2

II. INFLUENCE OF THE REACTION PARAMETERS 2

1. Electrodes 2

2. Temperature 3

3. Solvent 3

4. Cathode 3

5. Charge 4

6. Current intensity 4

7. Electrolyte 5

8. Concentration 5

III. CYCLIC VOLTAMMETRY 5

IV. FARADAY EFFICIENCY 8

V. SYNTHESIS OF ALPHA,AALPHA-DICHLORO ARYL COMPOUNDS 4 9

VI. SYNTHESIS OF ALPHA-CHLOROARYLACETIC ACID 1 12

VII. COPIES OF NMR SPECTRA 20

I. General information

Reactions were performed using oven dried glassware under inert atmosphere of nitrogen. Unless otherwise noted, all reagent-grade chemicals and solvents were obtained from commercial suppliers and were used as received. Toluene was dried over MBRAUN MB SPS-800 Apparatus. Reactions were monitored by thin-layer chromatography with silica gel 60 F254 pre-coated aluminum plates (0.25 mm). Visualization was performed under UV light, phosphomolybdic acid or $KMnO_4$ oxidation. Chromatographic purification of compounds was achieved with 60 silica gel (40-63 μm). Melting points were measured on a WME Köfler hot-stage (Stuart SMP3) and are uncorrected. Infrared spectra (IR) were recorded on a PerkinElmer Spectrum 100 Series FT-IR spectrometer. Liquids and solids were applied on the Single Reflection Attenuated Total Reflectance (ATR) Accessories. Data are reported in cm^{-1}. ^1H Spectra (300 MHz) and ^{13}C NMR spectra (75 MHz) were recorded on a Bruker Advance 300. Processing and analysis of the spectra were performed with the Topspin 3.6 software from Bruker on a PC workstation. Data appear in the following order: chemical shifts in ppm which were referenced to the internal solvent signal, number of protons, multiplicity (s, singlet; d, doublet; t, triplet; q, quadruplet; dd, doublet of doublet, ddd, doublet of doublet of doublet, dt, doublet of triplet; ddt, doublet of doublet of triplet, td, triplet of doublet; tdd, triplet of doublet of doublet; m, multiplet, ABq, AB system) and coupling constant J in Hertz. Accurate Mass measurements (HRMS) were performed by the Mass Spectrometry Laboratory of the University of Rouen and were recorded with a Waters LCP 1er XR spectrometer. The electrosynthesis were carried out by means of IKA ElectraSyn® 2.0 apparatus. Electrodes were all purchased from IKA®. Cyclic Voltammetry (CV) measurements were carried with an OrigaFlex® potentiostat/galvanostat by means of three electrodes.

II. Influence of the reaction parameters

1. Electrodes

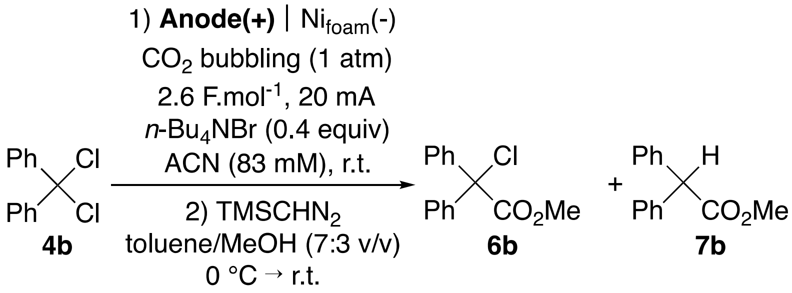

Entry	Anode	NMR yield **6b**[a]	NMR yield **7b**[a]
1	Mg	31	traces
**2**	**Al**	**37**	**traces**
3	Co	13	11

4	Zn	4	7

[a]: NMR yields were determined on ^1H NMR spectra of the crude product (after an esterification step with TMS diazomethane) in the presence of dimethyl terephtalate as an internal standard.

2. Temperature

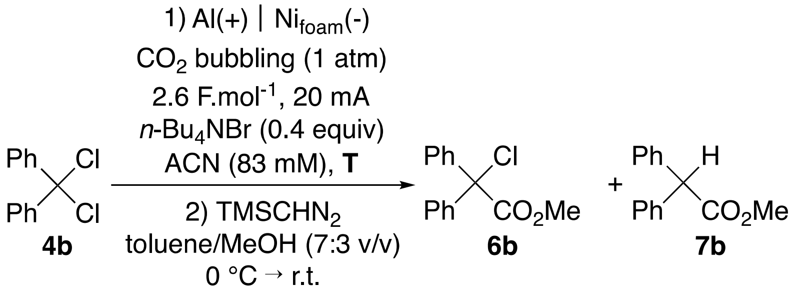

Entry	T	NMR yield **6b**[a]	NMR yield **7b**[a]
**1**	**rt**	**37**	**traces**
2	0 °C	29	13
3	50°C	13	11

[a]: NMR yields were determined on ^1H NMR spectra of the crude product (after an esterification step with TMS diazomethane) in the presence of dimethyl terephtalate as an internal standard.

3. Solvent

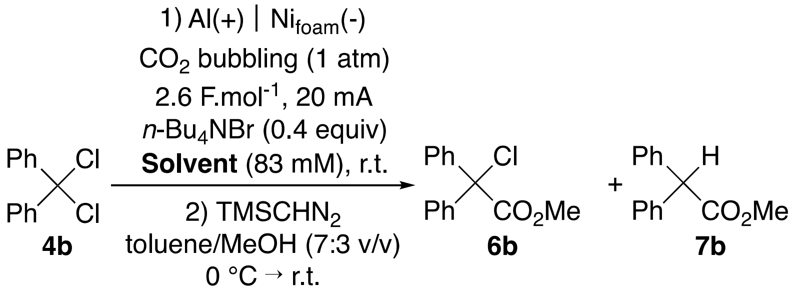

Entry	Solvent	NMR yield **6b**[a]	NMR yield **7b**[a]
1	ACN	37	traces
2	DMF	17	11
3	THF	nr	nr
4	DMF/THF (67/33)	nr	nr
**5**	**DMA**	**42**	**20**
6	DMA/CAN (33/67)	36	11
7	DMSO	traces	10

[a]: NMR yields were determined on ^1H NMR spectra of the crude product (after an esterification step with TMS diazomethane) in the presence of dimethyl terephtalate as an internal standard. nr : no reaction

4. Cathode

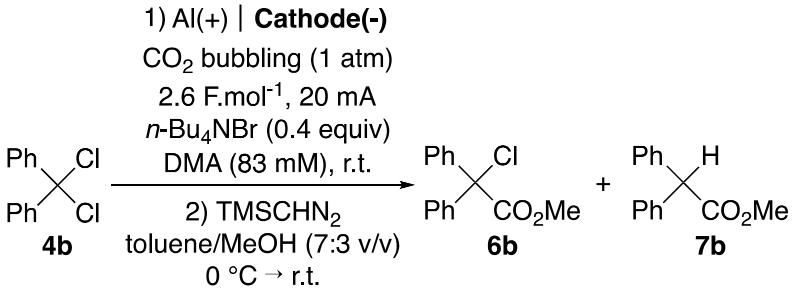

Entry	Cathode	NMR yield **6b**[a]	NMR yield **7b**[a]
1	Ni_{foam}	42	20
2	graphite	25	24
3	GC	32	26
4	Ni	36	10
5	Cu	30	7
6	Au	24	3
7	BDD	55	12
**8**	**SS**	**54**	**5**

[a]: NMR yields were determined on ^{1}H NMR spectra of the crude product (after an esterification step with TMS diazomethane) in the presence of dimethyl terephthalate as an internal standard. GC: glassy carbon. SS: stainless steel.

5. Charge

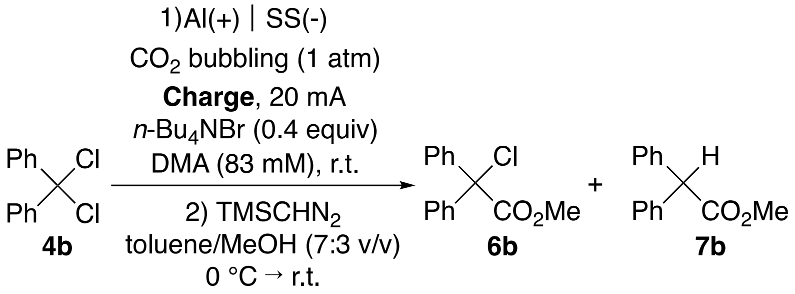

Entry	Charge (F.mol[-1])	NMR yield **6b**[a]	NMR yield **7b**[a]
1	1.8	33	Traces
2	2.3	38	3
**3**	**2.6**	**55**	**5**
4	2.9	50	10
5	3.2	54	11
6	3.8	58	16
7	4.2	44	23

[a]: NMR yields were determined on ^{1}H NMR spectra of the crude product (after an esterification step with TMS diazomethane) in the presence of dimethyl terephtalate as an internal standard. SS: stainless steel.

6. Current intensity

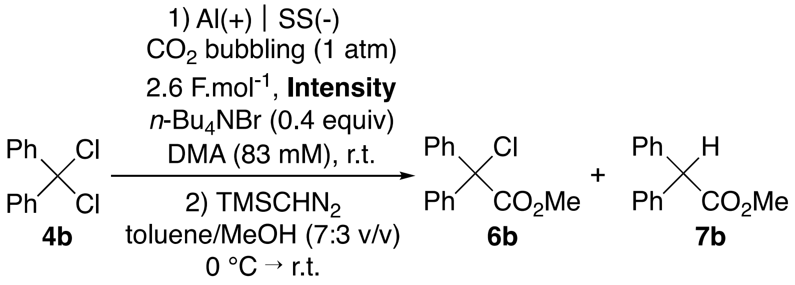

Entry	Intensity (mA)	NMR yield **6b**[a]	NMR yield **7b**[a]
1	10	39	6
2	15	54	13
**3**	**20**	**55**	**5**
4	25	53	8
5	30	50	8
6	40	46	11

[a]: NMR yields were determined on ^{1}H NMR spectra of the crude product (after an esterification step with TMS diazomethane) in the presence of dimethyl terephtalate as an internal standard. SS: stainless steel.

7. Electrolyte

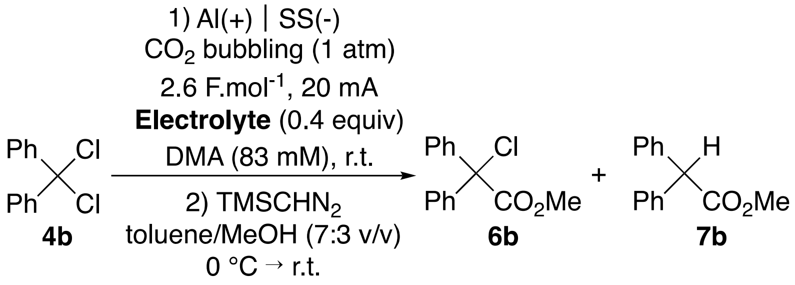

Entry	Electrolyte	NMR yield **6b**[a]	NMR yield **7b**[a]
1	*n*-Bu_4NBr	55	5
2	*n*-Bu_4NPF_6	54	13
3	*n*-Bu_4NBF_4	nr (high resistance)	
4	$LiClO_4$	nr (high resistance)	

[a]: NMR yields were determined on ^{1}H NMR spectra of the crude product (after an esterification step with TMS diazomethane) in the presence of dimethyl terephtalate as an internal standard. SS: stainless steel. nr: no reaction.

8. Concentration

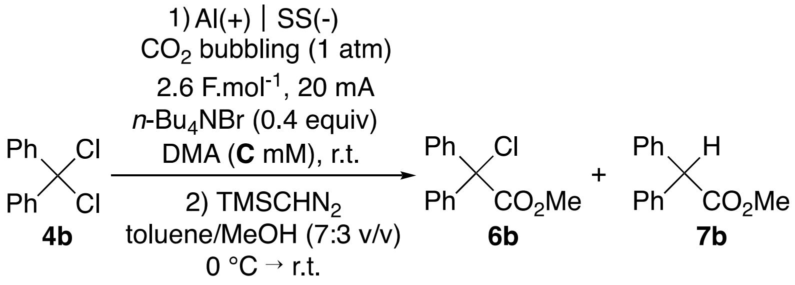

Entry	C (mM)	NMR yield **6b**[a]	NMR yield **7b**[a]
1	125	29	7
2	83	55	5
3	63	37	2
4	42	54	2
**5**	**35**	**55**	**<5**

[a]: NMR yields were determined on ^{1}H NMR spectra of the crude product (after an esterification step with TMS diazomethane) in the presence of dimethyl terephtalate as an internal standard. SS: stainless steel.

III. Cyclic voltammetry

Cyclic voltammetry measurements were recorded using a standard three-electrode setup in 14.0 mL of ACN or DMA with *n*-Bu_4NBF_4 (0.1 M) as the supporting electrolyte and substrate (0.01 M) at room temperature. The working electrode was a platinum electrode (100 μm diameter), the counter electrode was a platinum wire, and the reference was a Saturated-Calomel-Electrode (SCE) at a sweep rate of 200 or 300 mV.s^{-1}.

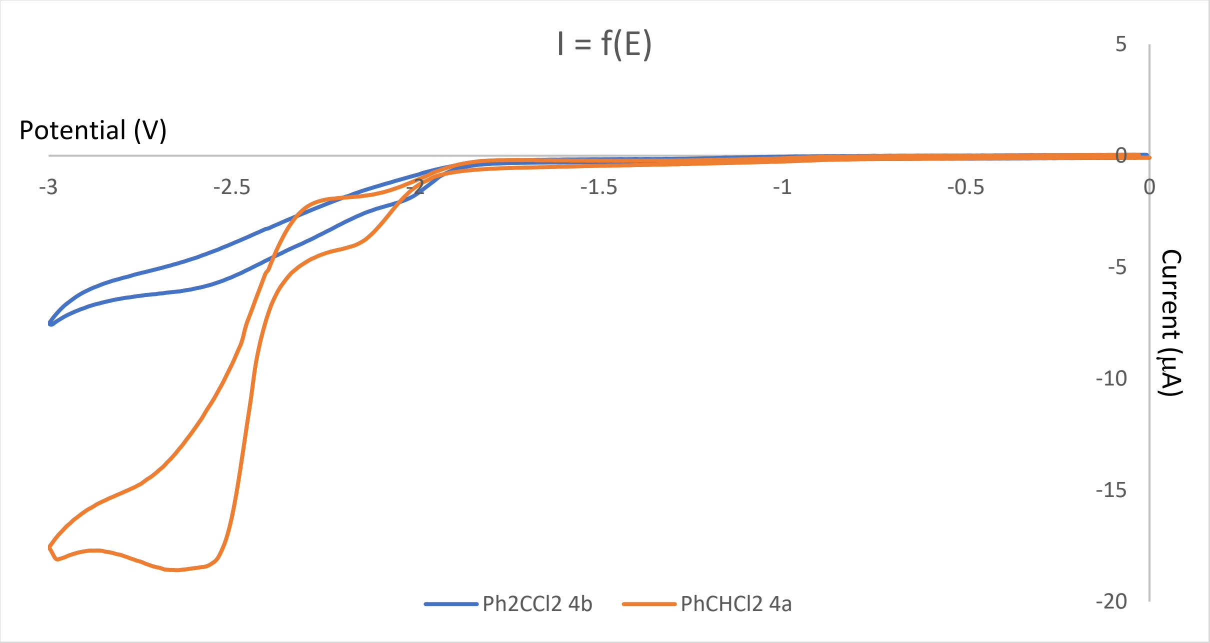

Figure S1. Cyclic voltammetry of **4a** and **4b** in ACN at 300 mV.S^{-1}.

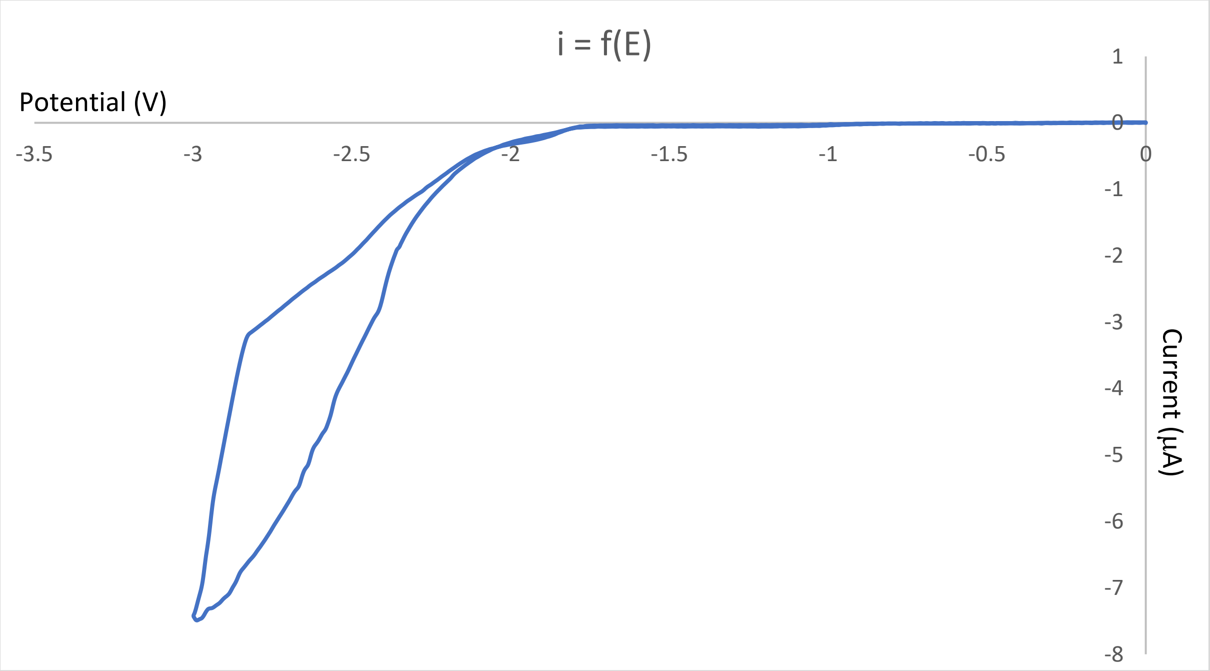

Figure S2. Cyclic voltammetry of **4a** in DMA at 200 mV.S^{-1}.

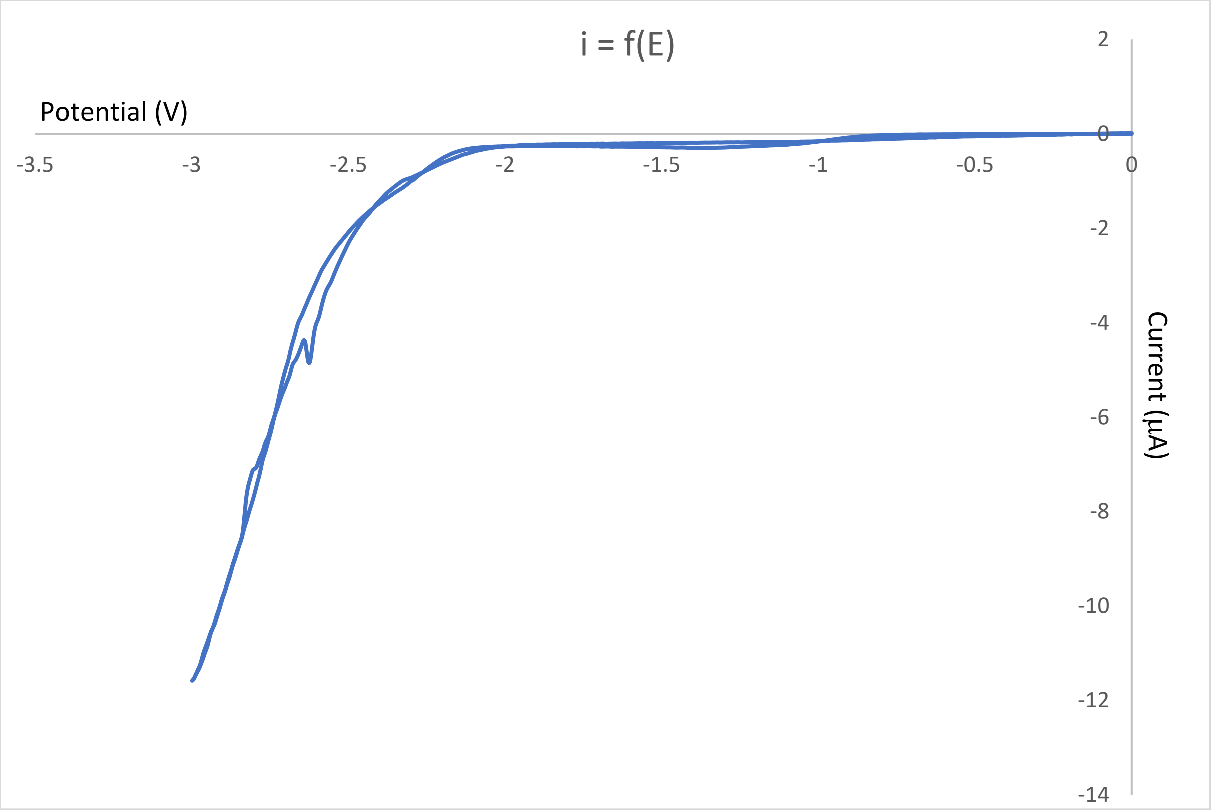

Figure S3. Cyclic voltammetry of **1a** in DMA at 200 mV.S^{-1}.

Whereas CV measurements of both **4a** (Figure S1, orange) and **4b** (Figure S1, blue) in ACN gave reduction potentials of respectively -2,07 V/SCE or -1.98 V/SCE (for the first reduction wave). Unfortunately, the measurement of **4a** in DMA gave unexploitable results (Figure S2) as well as the product of the reaction **1a** (Figure S3).

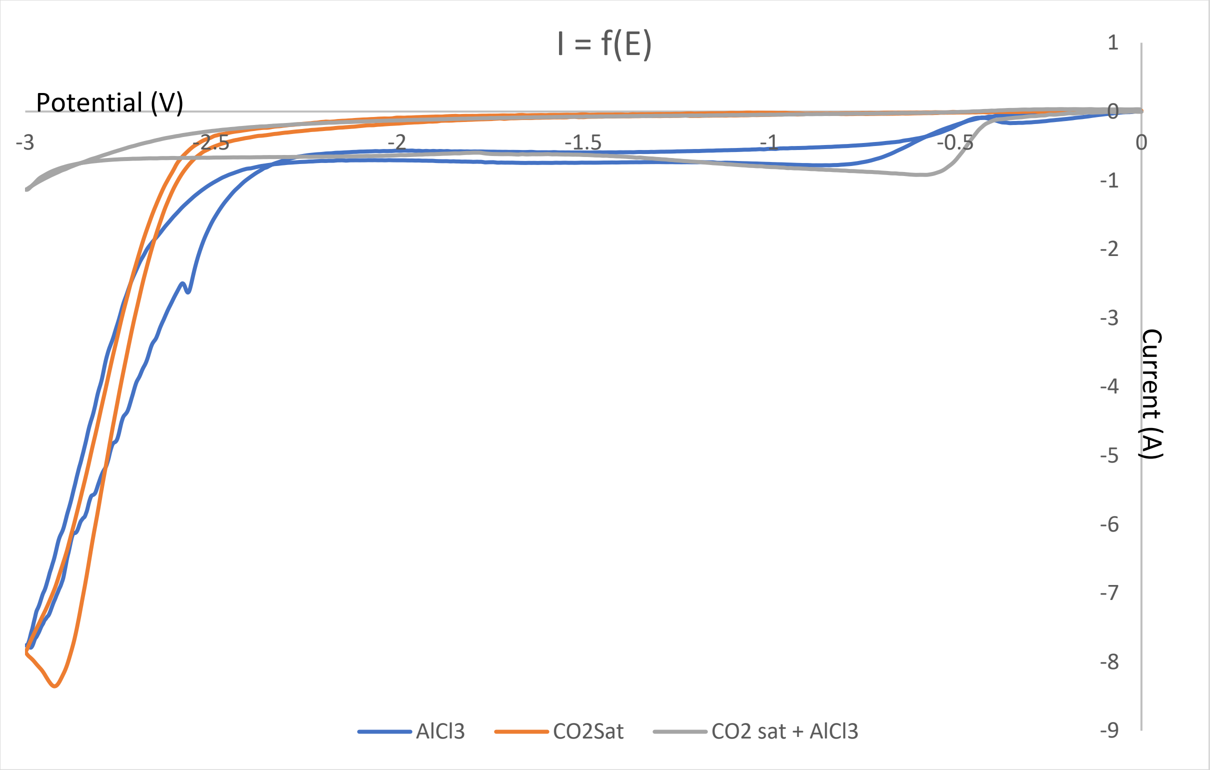

Figure S4. Cyclic voltammetry of $AlCl_3$, CO_2-saturated solution of DMA, mixture of $AlCl_3$ and CO_2-saturated solution of DMA at 200 mV.S^{-1}.

We have tried to study the influence of the Al^{3+} salts generated during the electrolysis. We performed a series of CV analysis in the presence of $AlCl_3$. We have recorded the CV curves for a CO_2-saturated solution (Figure S4, orange), $AlCl_3$ (Figure S4, blue) and a mix of CO_2-saturated solution + $AlCl_3$ (Figure S4, grey). Whereas the reduction wave of Al^{3+} around -0.5 V/SCE remained, it was difficult draw a clear-cut conclusion regarding the reduction wave of CO_2. Then, we wanted to study the influence of $AlCl_3$ on the reduction potential of $PhCHCl_2$. Unfortunately, a reaction occurred when mixing both reagents, making the recording of CV curves untrustworthy.

IV. Faraday efficiency

The Faraday efficiency (FE) was calculated for the electrosynthesis of the model product 1a which has been performed on both 0.25 mmol (50 % NMR yield, 47 % isolated yield) and 1.00 mmol (45 % NMR yield, 44 % isolated yield), according to the following equation:

$$FE = \frac{Q_{theo}}{Q_{exp}} \times 100 = \frac{n_e \times n_{prod} \times F}{i \times t} \times 100 = \frac{n_e \times (n_{reag} \times Y) \times F}{i \times t} \times 100$$

where n_e is the number of electrons added to or removed from one product molecule (2 in our case), n_{prod} the amount of product in mol, n_{reag} the amount of reagent used in mol, Y the isolated yield (%), F the Faraday constant (96485 C), i the current in A (20 mA) and t the time in seconds (3135 and 12540 seconds from 52 min and 3h29 respectively)

according to : P. Gandeepan, L. H. Finger, T. H. Meyer, L. Ackermann, *Chem. Soc. Rev.* **2020**, *49*, 4254-4272.

Calculation details for product **1a** on 0.25 mmol scale

$$FE = \frac{2 \times (0.00025 \times 0.47) \times 96485}{0.02 \times 3135} \times 100 = 36.2\ \%$$

Calculation details for product **1a** on 1 mmol scale

$$FE = \frac{2 \times (0.001 \times 0.44) \times 96485}{0.02 \times 12540} \times 100 = 33.8\ \%$$

V. Synthesis of α,α-dichloro aryl compounds **4**

General procedure for the synthesis of α,α-dichloro benzyl derivatives 4 [1]

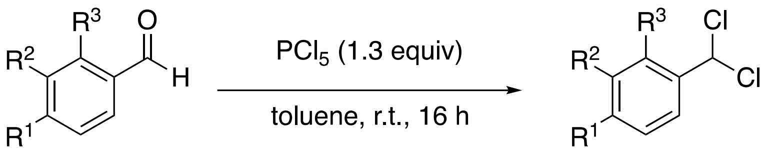

To a dry 10 mL flask containing PCl_5 (1.3 equiv) in dry toluene (5 mL) was added the corresponding aldehyde (1 equiv) and the mixture was then stirred for 16 hours. After completion, the reaction was diluted with 20 mL EtOAc and washed two times with 20 mL saturated $NaHCO_3$ aqueous solution then once with 10 mL brine. The organic layer was dried over $MgSO_4$, filtered, and concentrated at 40 °C under 80 mbar pressure. The residue was purified by silica gel column chromatography (100/0 EP:EtOAc to 95/5) to obtain the corresponding α,α-dichloro aryl compounds **4**. As mentioned by the authors and due their high reactivity, products **4** were generally used rapidly after their synthesis.

4a, **4j** and **4k** were commercially available and were used as received.

α,α-dichloro-4-methyltoluene **4c**

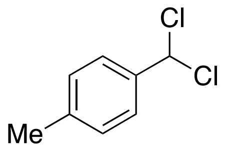

Following the general procedure with 4-methylbenzaldehyde (300 mg, 2.5 mmol), the title compound **4c** was obtained as a white solid (367 mg, 84 %). ^{1}H NMR (300 MHz, $CDCl_3$) δ 7.49 – 7.46 (m, 2H, CH_{Ar}), 7.23 – 7.20 (m, 2H, CH_{Ar}), 6.70 (s, 1H, C*H*), 2.39 (s, 3H, CH_3). ^{13}C NMR (75 MHz, $CDCl_3$) δ 140.3 (C_{Ar}), 137.8 (C_{Ar}), 129.6 (CH_{Ar}), 126.1 (CH_{Ar}), 72.0 (*C*H), 21.4 (CH_3). The spectral data were in agreement with those previously reported [2].

α,α-dichloro-4-fluorotoluene **4d**

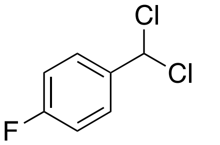

Following the general procedure with 4-fluorobenzaldehyde (310 mg, 2.5 mmol), the title compound **4d** was obtained as a colorless oil (376 mg, 84 %). ^{1}H NMR (300 MHz, $CDCl_3$) δ 7.59 – 7.55 (m, 2H, CH_{Ar}), 7.12 – 7.07 (m, 2H, CH_{Ar}), 6.70 (s, 1H, C*H*). ^{13}C NMR (75 MHz, $CDCl_3$) δ 163.3 (d, ^{1}J = 248.3 Hz, C_{Ar}), 136.4 (d, ^{4}J = 3.0 Hz, C_{Ar}), 128.2 (d, ^{3}J = 8.3 Hz, CH_{Ar}), 115.8 (d, ^{2}J = 21.8 Hz, CH_{Ar}), 70.9 (*C*H). ^{19}F NMR (282 MHz, $CDCl_3$) δ_F : -110.6. The spectral data were in agreement with those previously reported [3].

α,α-dichloro-4-(trifluoromethyl)toluene **4e**

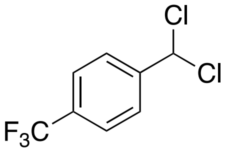

Following the general procedure with 4-(trifluoromethyl)benzaldehyde (435 mg, 2.5 mmol), the title compound **4e** was obtained as a colorless oil (560 mg, 96 %). ^{1}H NMR (300 MHz, $CDCl_3$) δ 7.73 – 7.66 (m, 4H, CH_{Ar}), 6.74 (s, 1H, C*H*). ^{19}F NMR (282 MHz, $CDCl_3$) δ -62.9. The spectral data were in agreement with those previously reported.**Error! Bookmark not defined.**

α,α-dichloro-4-phenyltoluene **4f**

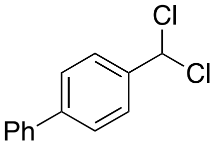

Following the general procedure with 4-phenylbenzaldehyde (455 mg, 2.5 mmol), the title compound **4f** was obtained as a white solid (415 mg, 70 %). ^{1}H NMR (300 MHz, $CDCl_3$) δ 7.65 – 7.58 (m, 6H, CH_{Ar}), 7.47 – 7.44 (m, 3H, CH_{Ar}), 6.77 (s, 1H, C*H*). ^{13}C NMR (75 MHz, $CDCl_3$) δ 143.0 (C_{Ar}), 140.1 (C_{Ar}), 139.3 (C_{Ar}), 128.9 (CH_{Ar}), 127.9 (C_{Ar}), 127.5 (CH_{Ar}), 127.2 (CH_{Ar}), 126.6 (CH_{Ar}), 71.6 (*CH*). The spectral data were in agreement with those previously reported [4].

α,α-dichloro-4-phenyltoluene **4g**

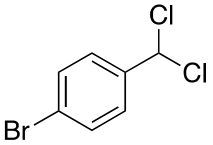

Following the general procedure with 3-methoxybenzaldehyde (460 mg, 2.5 mmol), the title compound **4g** was obtained as a colorless oil (510 mg, 85 %). ^{1}H NMR (300 MHz, $CDCl_3$) δ 7.56 – 7.53(m, 2H, CH_{Ar}), 7.46 – 7.44 (m, 2H, CH_{Ar}), 6.67 (s, 1H, C*H*). ^{13}C NMR (75 MHz, $CDCl_3$) δ 139.4 (C_{Ar}), 132.0 (CH_{Ar}), 127.8 (CH_{Ar}), 124.1 (C_{Ar}), 70.9 (*C*H). The spectral data were in agreement with those previously reported.**Error! Bookmark not defined.**

α,α-dichloro-3-methyltoluene **4h**

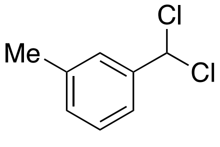

Following the general procedure with 3-methylbenzaldehyde (300 mg, 2.5 mmol), the title compound **4h** was obtained as a colorless oil (372 mg, 85 %). ^{1}H NMR (300 MHz, $CDCl_3$) δ 7.40 – 7.27 (m, 3H, CH_{Ar}), 7.21 (d, *J* = 7.4 Hz, 1H, CH_{Ar}), 6.69 (s, 1H, C*H*), 2.40 (s, 3H, CH_3). ^{13}C NMR (75 MHz, $CDCl_3$) δ 140.3 (C_{Ar}), 138.7 (C_{Ar}), 130.8 (CH_{Ar}), 128.7 (CH_{Ar}), 126.7 (CH_{Ar}), 123.2 (CH_{Ar}), 71.9 (*C*H), 21.4 (CH_3). The spectral data were in agreement with those previously reported.**Error! Bookmark not defined.**

α,α-dichloro-3-methoxytoluene **4i**

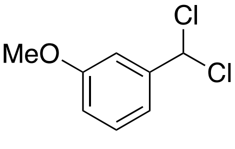

Following the general procedure with 3-methoxybenzaldehyde (340 mg, 2.5 mmol), the title compound **4i** was obtained as a colorless oil (439 mg, 92 %). ^{1}H NMR (300 MHz, $CDCl_3$) δ 7.30 – 7.22 (m, 1H, CH_{Ar}), 7.10 – 7.07 (m, 2H, CH_{Ar}), 6.91 – 6,87 (m, 1H, CH_{Ar}), 6.64 (s, 1H, C*H*), 5.81 (s, 3H, CH_3). ^{13}C NMR (75 MHz, $CDCl_3$) δ 159.8 (C_{Ar}), 141.7 (C_{Ar}), 129.8 (CH_{Ar}), 118.3 (CH_{Ar}), 115.8 (CH_{Ar}), 111.6 (CH_{Ar}), 71.7 (*C*H), 55.4 (OCH_3). The spectral data were in agreement with those previously reported.**Error! Bookmark not defined.**

α,α-dichloro-2-methyltoluene **4j**

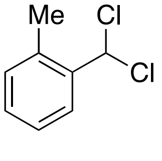

Following the general procedure with 2-methylbenzaldehyde (300 mg, 2.5 mmol), the title compound **4j** was obtained as a colorless oil (372 mg, 85 %). ^{1}H NMR (300 MHz; $CDCl_3$) δ 7.81 – 7.78 (m, 1H, CH_{Ar}), 7.31 – 7.28 (m, 2H, CH_{Ar}), 7.20 – 7.17 (m, 2H, CH_{Ar}), 6.95 (s, 1H, C*H*), 2.48 (s, 3H, CH_3). ^{13}C NMR (75 MHz; $CDCl_3$) δ 138.2 (C_{Ar}), 134.1 (C_{Ar}), 130.8 (CH_{Ar}), 129.9 (CH_{Ar}), 127.0 (CH_{Ar}), 126.8 (CH_{Ar}), 69.7 (C*H*), 18.8 (CH_3). The spectral data were in agreement with those previously reported.**Error! Bookmark not defined.**

α,α-dichloro-4-methoxytoluene **4l**

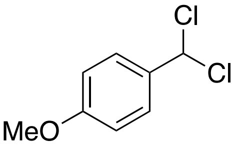

Following the general procedure with 4-methoxybenzaldehyde (340 mg, 2.5 mmol), the title compound **4l** was obtained as a colorless oil. The compound was very reactive and was thus used directly in the electrocarboxylation reaction. ^{1}H

NMR (300 MHz, $CDCl_3$) δ 7.51 (d, J = 8.7 Hz, 2H, CH_{Ar}), 6.91 (d, J = 8.7 Hz, 2H, CH_{Ar}), 6.70 (s, 1H, C*H*), 3.83 (s, 3H, CH_3). The spectral data were in agreement with those previously reported.**Error! Bookmark not defined.**

α,α-dichloro-4-methoxytoluene **4m**

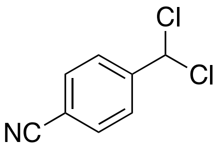

Following the general procedure with 4-cyanobenzaldehyde (328 mg, 2.5 mmol), the title compound **4m** was obtained as a colorless oil (437mg, 94%). ^{1}H NMR (300 MHz, $CDCl_3$) δ 7.74 – 7.67 (m, 4H, CH_{Ar}), 6.71 (1H, C*H*). ^{13}C NMR (75 MHz, $CDCl_3$) δ 144.7 (C_{Ar}), 132.8 (CH_{Ar}), 127.1 (CH_{Ar}), 118.0 (*C*N or C_{Ar}), 113.9 (*C*N or C_{Ar}), 70.3 (*C*H). The spectral data were in agreement with those previously reported [5].

VI. Synthesis of α-chloroarylacetic acid 1

General procedure A for the synthesis of α-chloroarylacetic acid derivatives 1

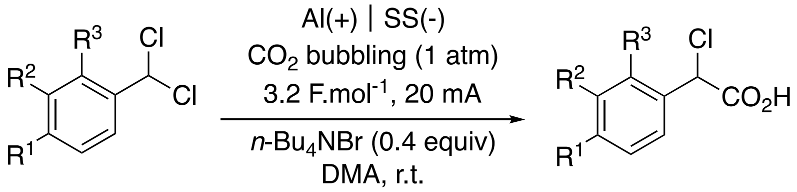

In a dry electrochemical cell (10 mL vial from IKA) containing n-Bu_4NBr (33 mg, 0.1 mmol, 0.4 equiv) was added DMA (7 mL) and α,α-dichloro aryl derivates **4** (0.25 mmol, 1 equiv) under nitrogen. The electrodes (aluminum electrode as anode and stainless steel as cathode) were installed, and carbon dioxide was bubbled through the solution for 5 minutes using a CO_2 balloon. The reaction was carried out at room temperature for 48 minutes, with a constant current of 20 mA and a charge of 3.2 F.mol^{-1}. After completion, the electrodes were rinsed subsequently with EtOAc, HCl 1M then water. The reaction mixture was diluted with 15 mL of EtOAc and washed with 15 mL of HCl 0.5 M. The aqueous phase was washed a second time with 10 mL of EtOAc then the combined organic extract was washed three time with 10 mL of HCl 0.5 M. The organic layer was dried over $MgSO_4$, filtered, and concentrated under reduced pressure. The NMR yield was measured by ^{1}H NMR of crude by means of dimethyl terephthalate as internal standard (0.25 equiv, 12.2 mg). The residue was purified by silica gel column chromatography (5/25/70 MeOH:CH_2Cl_2:PE, then 1/20/79 HCOOH:Et_2O:PE) to obtain the corresponding α-chloroarylacetic acid **1**.

1 mmol procedure

In a dry electrochemical cell (20 mL vial from IKA) containing *n*-Bu_4NBr (110 mg, 0.34 mmol, 0.34 equiv) was added DMA (16 mL) and α,α-dichlorotoluene **4a** (126 μL, 1.00 mmol, 1 equiv) under nitrogen. The electrodes (aluminum electrode as anode and stainless steel as cathode) were installed, and carbon dioxide was bubbled through the solution for 5 minutes using a CO_2 balloon. The reaction was carried out at room temperature for 48 minutes, with a constant current of 20 mA and a charge of 3.2 $F.mol^{-1}$. After completion, the electrodes were rinsed subsequently with EtOAc, HCl 1M then water. The reaction mixture was diluted with 30 mL of EtOAc and washed with 30 mL of HCl 0.5 M. The aqueous phase was washed a second time with 20 mL of EtOAc then the combined organic extract was washed three time with 20 mL of HCl 0.5 M. The organic layer was dried over $MgSO_4$, filtered, and concentrated under reduced pressure. The residue was purified by silica gel column chromatography (5/25/70 MeOH:CH_2Cl_2:PE, then 1/20/79 HCOOH:Et_2O:PE) to obtain the corresponding 2-chloro-2-phenylacetic acid **1a** as a white solid (75 mg, 44%).

2-chloro-2-phenylacetic acid **1a**

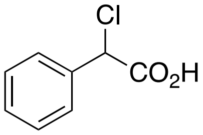

Following the general procedure A with **4a** (40 mg, 0.25 mmol), the title compound **1a** was obtained as a white solid (20 mg, 47%). ^1H NMR (300 MHz, $CDCl_3$) δ 11.02 (bs, 1H, CO_2H), 7.48 – 7.45 (m, 2H, CH_{Ar}), 7.36 – 7.33 (m, 3H, CH_{Ar}), 5.33 (s, 1H, C*H*). ^{13}C NMR (75 MHz, $CDCl_3$) δ 174.4 (CO_2H), 135.0 (C_{Ar}), 129.6 (CH_{Ar}), 129.0 (CH_{Ar}), 128.0 (CH_{Ar}), 58.8 (*C*H). The spectral data were in agreement with those previously reported [6].

2-chloro-2-(4-methylphenyl)acetic acid **1c**

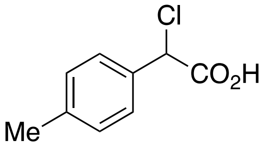

Following the general procedure A with **4c** (46 mg, 0.26 mmol), the title compound **1c** was obtained as a white solid (26 mg, 54%). mp = 89-90 °C. ^1H NMR (300 MHz, $CDCl_3$) δ 10.55 (bs, 1H, CO_2H), 7.40 – 7.38 (d, J = 8.2 Hz, 2H, CH_{Ar}), 7.21 – 7.19 (d, J = 8.2 Hz, 2H, CH_{Ar}), 5.35 (s, 1H, C*H*), 2.36 (s, 3H, CH_3). ^{13}C NMR (75 MHz, $CDCl_3$) δ 174.3 (CO_2H), 139.8 (C_{Ar}), 132.0 (C_{Ar}), 129.7 (CH_{Ar}), 127.9 (CH_{Ar}), 58.6 (*C*H), 21.3 (CH_3). IR (neat) ν 2323, 1721, 1512, 1412, 1286, 1201, 912, 804, 787 cm^{-1}. HRMS (TOF-ESI^-): calcd for $C_9H_8Cl_2O_2$ [$(M-H)^-$]: 183.0218; found: 183.0206.

2-chloro-2-(4-fluorophenyl)acetic acid **1d**

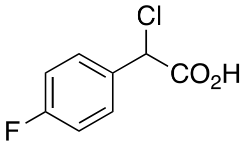

Following the general procedure A with **4d** (47 mg, 0.25 mmol), the title compound **1d** was obtained as a white solid (25 mg, 53%). mp = 83-84 °C. ^{1}H NMR (300 MHz, $CDCl_3$) δ 8.60 – 7.90 (bs, 1H, CO_2*H*), 7.54-7.47 (m, 2H, C*H*$_{Ar}$), 7.12 – 7.04 (m, 2H, C*H*$_{Ar}$), 5.36 (s, 1H, C*H*). ^{13}C NMR (75 MHz, $CDCl_3$) δ 173.4 (*C*O_2H), 163.3 (d, ^{1}J = 248.3 Hz, *C*$_{Ar}$), 130.9 (d, ^{4}J = 3.8 Hz, *C*$_{Ar}$), 130.0 (d, ^{3}J = 9.0 Hz, *C*H$_{Ar}$), 116.1 (d, ^{2}J = 21.8 Hz, *C*H$_{Ar}$), 57.8 (*C*H). ^{19}F NMR (282 MHz, $CDCl_3$) δ -111.1. IR (neat) ν 3072, 1733, 1601, 1508, 1422, 1286, 1230, 1203, 1161, 806, 839, 690, 525 cm^{-1}. HRMS (TOF-ESI^{-}): calcd for $C_8H_5ClFO_2$ [(M-H)$^{-}$]: 186.9968; found: 186.9962.

2-chloro-2-(4-trifluoromethylphenyl)acetic acid **1e**

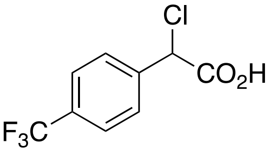

Following the general procedure A with **4e** (57 mg, 0.25 mmol), the title compound **1e** was obtained as an oil (22 mg, 37%). ^{1}H NMR (300 MHz, $CDCl_3$) δ 8.54 (bs, 1H, CO_2*H*), 7.63 – 7.57 (m, 4H, C*H*$_{Ar}$), 5.37 (s, 1H, C*H*). ^{13}C NMR (75 MHz, $CDCl_3$) δ 173.2 (*C*O_2H), 139.0 (*C*$_{Ar}$), 131.7 (q, ^{1}J = 32.3 Hz, *C*F_3), 128.4 (*C*H$_{Ar}$), 125.9 (*C*H$_{Ar}$), 121.8 (*C*H$_{Ar}$), 58.4 (*C*H). ^{19}F NMR (282 MHz, $CDCl_3$) δ -62.91. HRMS (TOF-ESI^{-}): calcd for $C_8H_5ClF_3$ [(M-CO_2-H)$^{-}$]: 193.0037; found: 193.0024.

2-chloro-2-(4-phenylphenyl)acetic acid **1f**

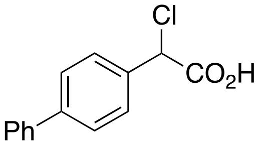

Following the general procedure A with **4f** (59 mg, 0.25 mmol), the title compound **1f** was obtained as an offwhite solid (20 mg, 32%). mp = 143-144 °C. ^{1}H NMR (300 MHz, $CDCl_3$) δ 7.64 – 7.56 (m, 6H, C*H*$_{Ar}$), 7.48 – 7.26 (m, 3H, C*H*$_{Ar}$), 6.43 (bs, 1H, CO_2*H*) 5.44 (s, 1H, C*H*). ^{13}C NMR (75 MHz, $CDCl_3$) δ 173.5 (*C*O_2H), 142.8 (*C*$_{Ar}$), 140.3 (*C*$_{Ar}$), 134.0 (*C*$_{Ar}$), 129.0 (*C*H$_{Ar}$), 128.6 (*C*H$_{Ar}$), 128.0 (*C*$_{Ar}$), 127.9 (*C*H$_{Ar}$), 127.3 (*C*H$_{Ar}$), 58.6 (*C*H). IR (neat) ν 1717, 1488, 1408, 1278, 1197, 833, 753, 692 cm^{-1}. HRMS (TOF-ESI^{-}): calcd for $C_{14}H_{10}Cl_2O_2$ [(M-H)$^{-}$]: 245.0375; found: 245.0369.

2-chloro-2-(4-bromophenyl)acetic acid **1g**

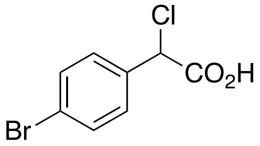

Following the general procedure A with **4g** (60 mg, 0.25 mmol), the title compound **1g** was obtained as a white solid (30 mg, 48 %). mp = 82-83 °C. ^{1}H NMR (300 MHz, $CDCl_3$) δ 9,35 (br s, 1H, CO_2*H*), 7.54 – 7.51 (dt, *J* = 8.6, 2.5 Hz, 2H, C*H*$_{Ar}$), 7.41 – 7.35 (dt, *J* = 8.6, 2.5 Hz, 2H, C*H*$_{Ar}$), 5.32 (s, 1H, C*H*). ^{13}C NMR (75 MHz, $CDCl_3$) δ 173.6 (*C*O_2H), 133.9 (*C*$_{Ar}$), 132.2 (*C*H$_{Ar}$), 129.7 (*C*H$_{Ar}$), 124.0 (*C*$_{Ar}$), 57.9 (*C*H). IR (neat) ν 1718, 1490, 1417, 1276, 1202, 1013, 802, 149, 670, 511 cm^{-1}. HRMS (TOF-ESI^{-}): calcd for $C_8H_5ClBrO_2$ [(M-H)$^{-}$]: 246.9167; found: 246.9157.

2-chloro-2-(3-methylphenyl)acetic acid **1h**

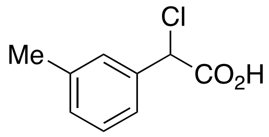

Following the general procedure A with **4h** (46 mg, 0.26 mmol), the title compound **1h** was obtained as a white solid (18 mg, 36%). mp = 77-78 °C. ^{1}H NMR (300 MHz, CDCl$_3$) *δ* 9.69 (bs, 1H, CO$_2$*H*), 7.35 – 7.27 (m, 3H, C*H*$_{Ar}$), 7.21 – 7.16 (m, 1H, C*H*$_{Ar}$), 5.34 (s, 1H, C*H*), 2.37 (s, 3H, C*H*$_3$). ^{13}C NMR (75 MHz, CDCl$_3$) *δ* 173.9 (*C*O$_2$H), 138.9 (*C*$_{Ar}$), 134.9 (*C*$_{Ar}$), 130.5 (*C*H$_{Ar}$), 128.9 (*C*H$_{Ar}$), 128.6 (*C*H$_{Ar}$), 125.1 (*C*H$_{Ar}$), 58.8 (*C*H), 21.4 (*C*H$_3$). IR (neat) ν 3051, 2811, 1724, 1490, 1402, 1199, 885, 776, 715, 645 cm^{-1}. HRMS (TOF-ESI$^-$): calcd for $C_9H_8ClO_2$ [(M-H)$^-$]: 183.0218; found: 183.0215.

2-chloro-2-(3-methoxyphenyl)acetic acid **1i**

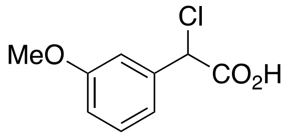

Following the general procedure A with **4i** (48 mg, 0.25 mmol), the title compound **1i** was obtained as an oil (28 mg, 55%). ^{1}H NMR (300 MHz, CDCl$_3$) *δ* 7.47 (bs, 1H, CO$_2$*H*), 7.32 – 7.27 (m, 1H, C*H*$_{Ar}$), 7.09 – 7.06 (m, 2H, C*H*$_{Ar}$), 6.94 – 6.90 (m, 1H, C*H*$_{Ar}$), 5.34 (s, 1H, C*H*), 3.82 (s, 3H, C*H*$_3$). ^{13}C NMR (75 MHz, CDCl$_3$): *δ* 173.4 (*C*O$_2$H), 159.9 (*C*$_{Ar}$), 136.4 (*C*$_{Ar}$), 130.0 (*C*H$_{Ar}$), 120.3 (*C*H$_{Ar}$), 115.3 (*C*H$_{Ar}$), 113.4 (*C*H$_{Ar}$), 58.8 (*C*H), 55.4 (O*C*H$_3$). IR (neat) ν 3065, 1731, 1598, 1492, 1465, 1265, 1203, 1161, 925, 780, 719 cm^{-1}. HRMS (TOF-ESI$^-$): calcd for $C_9H_8ClO_3$ [(M-H)$^-$]: 199.0167; found: 199.0162.

2-chloro-2-(2-methylphenyl)acetic acid **1j**

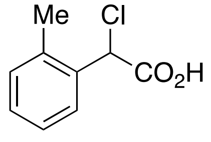

Following the general procedure A with **4j** (46 mg, 0.26mmol), the title compound **1j** was obtained as a white solid (23 mg, 48 %). mp = 72-73 °C. ^{1}H NMR (300 MHz, CDCl$_3$) *δ* 7.54 – 7.51 (m, 1H, C*H*$_{Ar}$), 7.28 – 7.19 (m, 3H, C*H*$_{Ar}$), 5.66 (s, 1H, C*H*), 2.45 (s, 3H, C*H*$_3$). ^{13}C NMR (75 MHz, CDCl$_3$) *δ* 173.6 (*C*O$_2$H), 136.3 (*C*$_{Ar}$), 133.7 (*C*$_{Ar}$), 130.9 (*C*H$_{Ar}$), 129.5 (*C*H$_{Ar}$), 128.1 (*C*H$_{Ar}$), 126.9 (*C*H$_{Ar}$), 56.0 (*C*H), 19.3 (*C*H$_3$). IR (neat) ν 3070, 1731, 1287, 12, 726 cm^{-1}. HRMS (TOF-ESI$^-$): calcd for $C_9H_8ClO_2$ [(M-H)$^-$]: 183.0218; found: 183.0213.

2-chloro-2-(4-methoxyphenyl)acetic acid **1l**

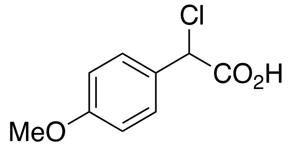

Following the general procedure A with **4l** (51 mg, 0.27 mmol), a crude mixture of the title compound **1l** was obtained and the yield (39%) was determined by ^{1}H NMR analysis using dimethyl terephthalate (0.25 equiv) as internal standard. Compound **1l** was found to be sensitive to the flash chromatography conditions affording product **10** (*vide infra*).

2-chloro-2-(4-cyanophenyl)acetic acid **1m**

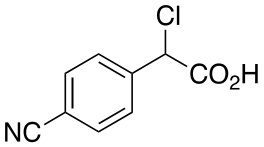

Following the general procedure A with **4m** (47 mg, 0.25 mmol), a crude mixture of the title compound **1m** was obtained and the yield (25%) was determined by ^{1}H NMR analysis using dimethyl terephthalate (0.25 equiv) as internal standard. After purification, **1m** was obtained as an inseparable mixture. ^{1}H NMR (300 MHz, $CDCl_3$) δ 7.71 – 7.62 (m, 4H, CH_{Ar}), 5.39 (s, 1H, C*H*).

Synthesis of methyl 2-chloro-2-phenylacetate 6a

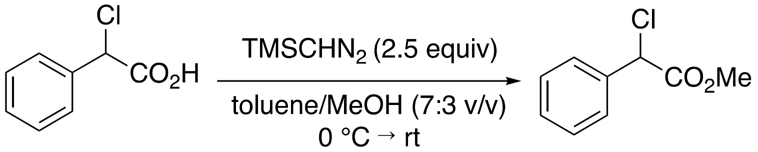

The 2-chloro-2-phenylacetic acid **1a** (1.00 g, 5.86 mmol, 1 equiv) was then dissolved in toluene/methanol mixture (10 mL, 7:3 v/v) and cooled to 0 °C. TMS diazomethane (2M in hexanes, 2.17 mL, 14.7 mmol, 2.5 equiv) was then added until a yellow coloration persists in the solution. The solution was then stirred for 30 min at 0 °C then 30 min at r.t. The solution was then quenched with acetic acid (0.1 mL), MeOH was evaporated, and the residual toluene solution was diluted with EtOAc. The organic phase was washed with HCl 1M, saturated aqueous $NaHCO_3$ solution then water. The organic layer was dried over $MgSO_4$, filtered, and concentrated under reduced pressure. Methyl 2-chloro-2-phenylacetate derivatives **6** (1.06 g, 5.74 mmol, 98%) were obtained as a transparent oil.

Methyl 2-chloro-2-phenylacetate **6a**

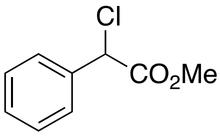

^{1}H NMR (300 MHz, $CDCl_3$) δ 7.51 – 7.48 (m, 2H, CH_{Ar}), 7.40 – 7.36 (m, 3H, CH_{Ar}), 5.37 (s, 1H, C*H*), 3.78 (s, 3H, CH_3). Spectroscopic data were in agreement with those previously reported [7].

General procedure B for the synthesis of methyl 2-chloro-2-phenylacetate derivatives 6b,k

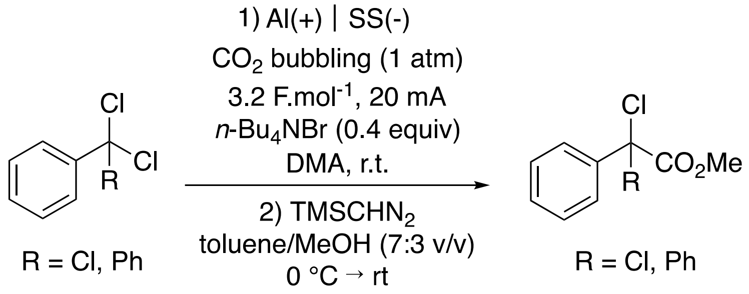

In a dry electrochemical cell (10 mL vial from IKA) containing *n*-Bu_4NBr (33 mg, 0.1 mmol, 0.4 equiv) was added DMA (7 mL) and α,α-dichloro aryl derivates **4** (0.25 mmol, 1 equiv)) under nitrogen. The electrodes (aluminum electrode as anode and stainless steel as cathode) were installed, and carbon dioxide was bubbled through the solution for 5 minutes using a CO_2 balloon. The reaction was carried out at room temperature for 48 minutes, with a constant current of 20 mA and a charge of 3.2 F.mol^{-1}. After completion, the electrodes were rinsed subsequently with EtOAc, HCl 1M then water. The reaction mixture was diluted with 15 mL of EtOAc and washed with 15 mL of HCl 0.5 M. The aqueous phase was washed a second time with 10 mL of EtOAc then the combined organic extract was washed three time with 10 mL of HCl 0.5 M. The organic layer was dried over $MgSO_4$, filtered, and concentrated under reduced pressure. The crude mixture was then dissolved in toluene/methanol mixture (7:3 v/v) and cooled to 0 °C. TMS diazomethane was then added until a yellow coloration persists in the solution. The solution was then stirred for 15 min at 0 °C then 15 min at r.t. The solution was then quenched with acetic acid and extracted with EtOAc and washed with saturated aqueous $NaHCO_3$ solution, HCl 1M then water. The NMR yield was measured by ^{1}H NMR of crude by means of dimethyl terephthalate as internal standard (0.25 equiv, 12.2 mg). The residue was purified by silica gel column chromatography (5/25/70 MeOH:CH_2Cl_2:PE, then 1/20/79 HCOOH:Et_2O:PE) to obtain the corresponding methyl 2-chloro-2-phenylacetate derivatives **6**.

Methyl 2-chloro-2,2-diphenylacetate **6b**

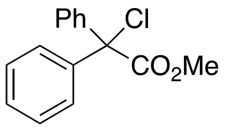

Following the general procedure B with **4b** (59 mg, 0.25 mmol), the title compound **6b** was obtained as colorless oil (31 mg, 48 %). ^{1}H NMR (300 MHz, $CDCl_3$) δ 7.42 – 7.26 (m, 10H, CH_{Ar}), 3.78 (s, 3H, CH_3). Spectroscopic data were in agreement with those previously reported [8].

Methyl 2,2-dichloro-2-phenylacetate **6k**

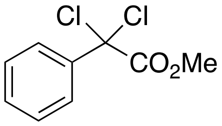

Following the general procedure B with **4k** (48.9 mg, 0.25 mmol), the title compound **6k** was obtained as a colorless oil (12.5 mg, 23 %). ^{1}H NMR (300 MHz, $CDCl_3$) δ 7.73 – 7.68 (m, 2H, CH_{Ar}), 7.45 – 7.39 (m, 3H, CH_{Ar}), 3.78 (s, 3H, CH_3). Spectroscopic data were in agreement with those previously reported [9].

Procedure for the synthesis of 2-methoxy-2-(4-methoxyphenyl)acetic acid 10

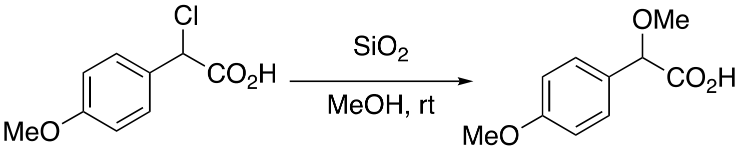

Compound **10** was obtained after a slight modification of the general procedure A. The crude product was dissolved in MeOH, SiO_2 was added, and the resulting mixture was stirred overnight at room temperature. After filtration and evaporation of MeOH, the resulting mixture was purified according to general procedure A. Starting from **1l** (48 mg, 0.25 mmol), the title compound **10** was obtained as a white solid (12 mg, 24 %).

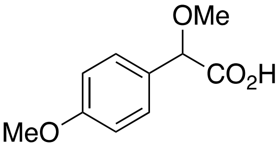

^{1}H NMR (300 MHz, $CDCl_3$) δ 7.37 – 7.32 (m, 2H, CH_{Ar}), 6,93 – 6.88 (m, 2H, CH_{Ar}), 4.73 (s, 1H, CH), 3.81 (s, 3H, CH_3), 3.39 (s, 3H, CH_3). Spectroscopic data were in agreement with those previously reported [10].

Procedure for the synthesis of *N*-benzyl-2-chloro-2-phenylacetamide 11

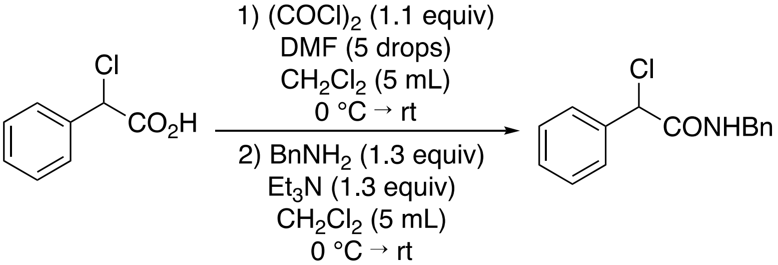

Product **11** was synthesized following the procedure reported in the literature [11] as following: oxalyl chloride (273 μL, 3.22 mmol, 1.1 equiv) and DMF (5 drops) were added to a 0 °C solution of 2-chloro-2-phenylacetic acid **1a** (500 mg, 2.93 mmol, 1 equiv) in anhydrous CH_2Cl_2 (5 mL) in an oven-dried Schlenk tube. The reaction mixture was allowed to warm to room temperature with stirring for 1 h. The solution was then transferred by cannula to a solution of benzyl amine (417 μL, 3.81 mmol, 1.3 equiv) and triethylamine (514 μL, 3.81 mmol, 1.3 equiv) in anhydrous CH_2Cl_2 (5 mL) at 0 °C. The

suspension was stirred for 4 h, and then the reaction was quenched by the addition of HCl (1 M). The reaction mixture was extracted with CH_2Cl_2 (25 mL × 3), and the combined organic layers were washed with brine, dried over Na_2SO_4, and concentrated. The residue was purified by flash chromatography (hexane/ dichloromethane = 2/1) which furnished the desired product (450 mg, 59%) as white solid.

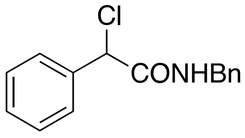

^{1}H NMR (300 MHz, $CDCl_3$) *δ* *δ* 7.46 – 7.27 (m, 10H, CH_{Ar}), 7,00 (br s, 1H, N*H*) 5.43 (s, 1H, C*H*), 4.52 (d, ^3J = 5.8 Hz, 2H, CH_2). Spectroscopic data were in agreement with those previously reported [12].

Procedure for the synthesis of methyl 2-azido-2-phenylacetate 12

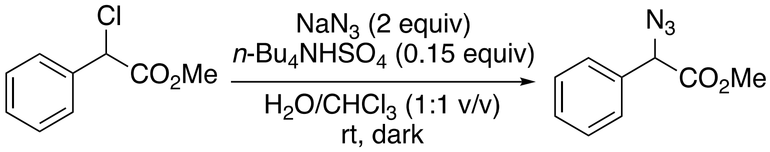

The title product was synthesized according to a reported procedure [13] as followed: Methyl 2-chloro-2-phenylacetate **6a** (194 mg, 1 mmol, 1 equiv) was mixed with NaN_3 (130 mg, 2 mmol, 2 equiv) and tetrabutylammonium hydrogensulfate (50 mg, 0.15 mmol, 0,15 equiv) in a mixture of water (1 mL) and chloroform (1 mL). The reaction was allowed to stir at room temperature for 24 h in the dark, after which time the aqueous layer was removed and the organic layer was washed with water (3 x 10 mL) and dried over sodium sulfate. The solvent was removed carefully by rotary evaporation under reduced pressure without heating. The title compound **12** was obtained as a colorless oil (191 mg, 99 %).

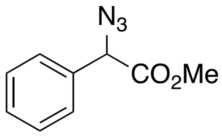

^{1}H NMR (300 MHz, $CDCl_3$) *δ* 7.41 – 7.37 (m, 5H, CH_{Ar}), 4,99 (s, 1H, C*H*), 3.76 (s, 3H, CH_3). Spectroscopic data were in agreement with those previously reported [14].

VII. Copies of NMR spectra

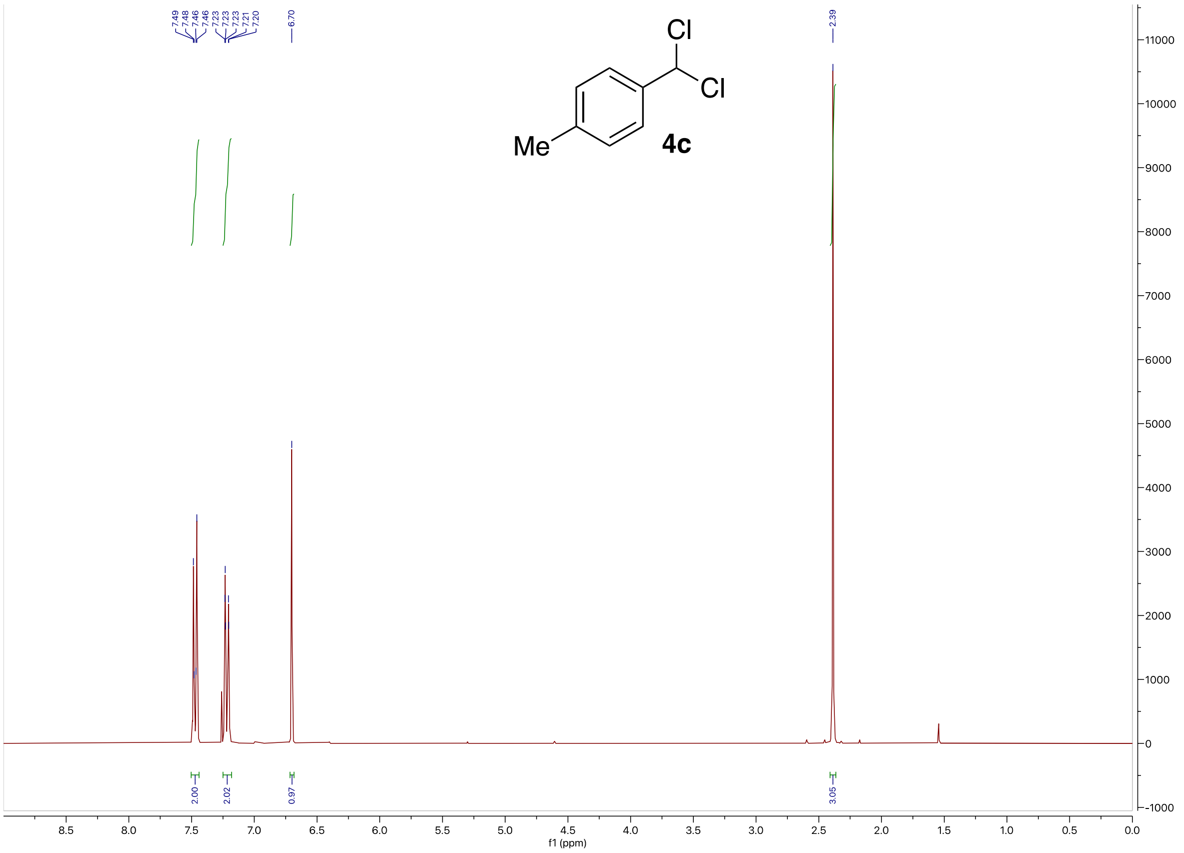

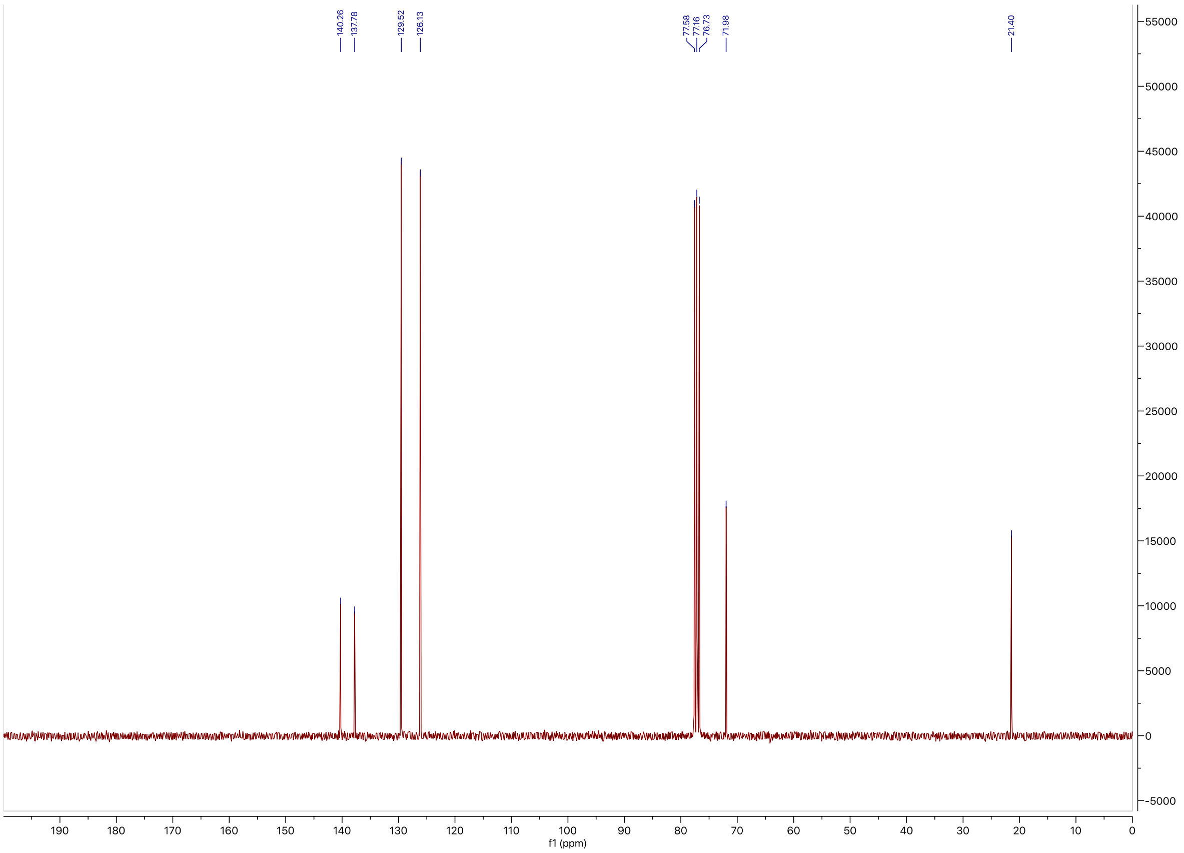

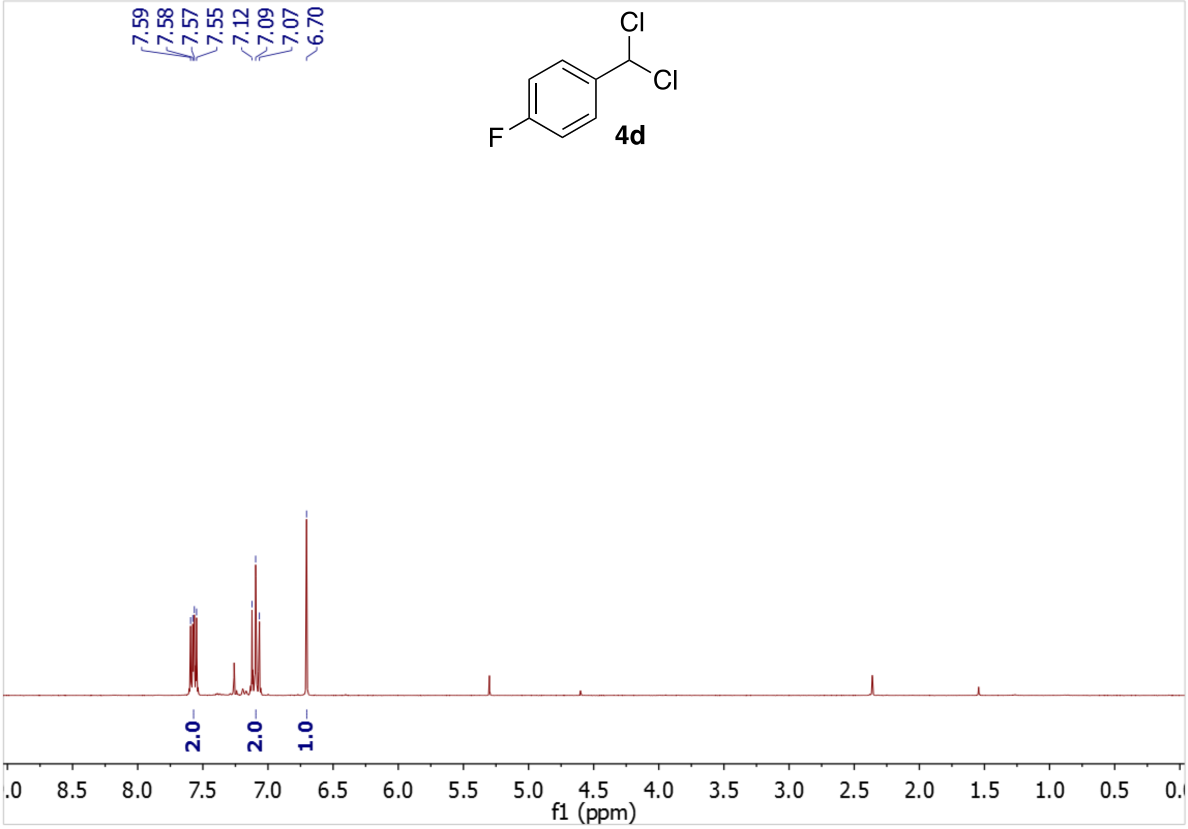
7.59
7.58
7.57
7.55
7.12
7.09
7.07
6.70
Cl
Cl
F
4d
2.0
2.0
1.0
f1 (ppm)

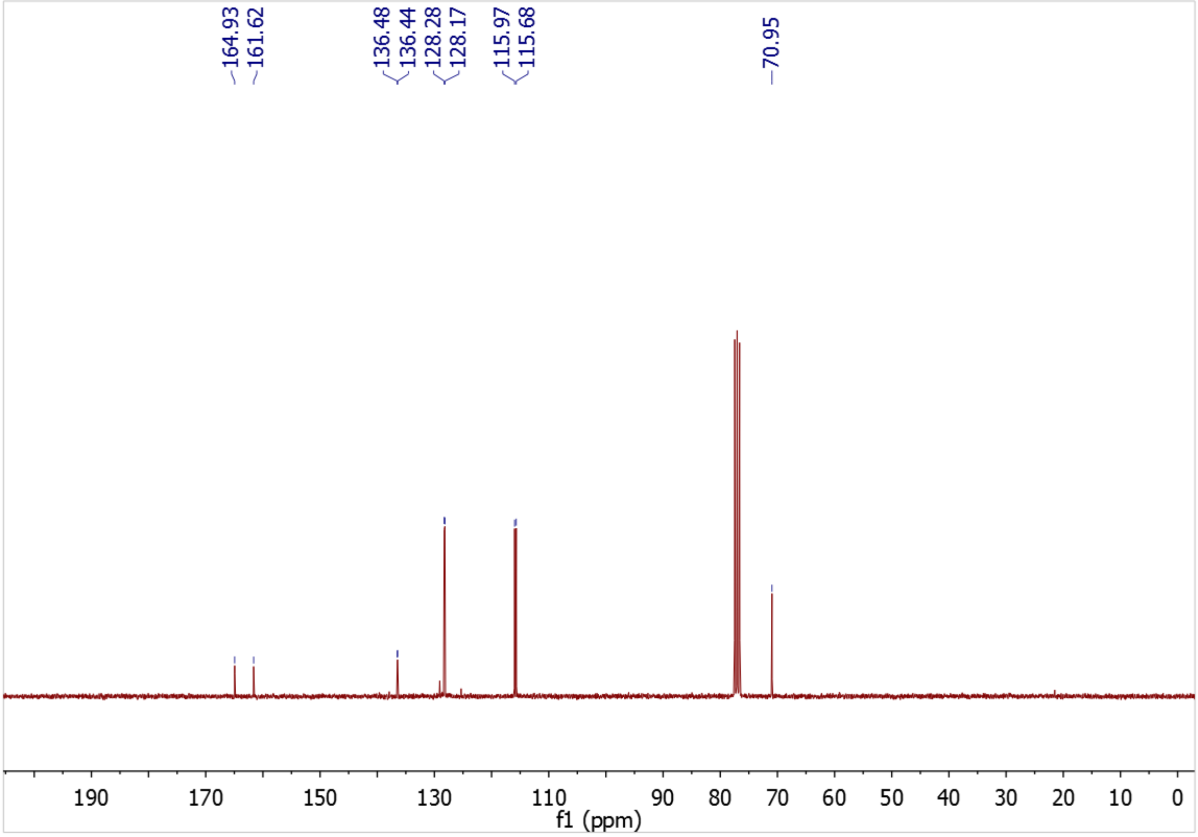
164.93
161.62
136.48
136.44
128.28
128.17
115.97
115.68
70.95
f1 (ppm)

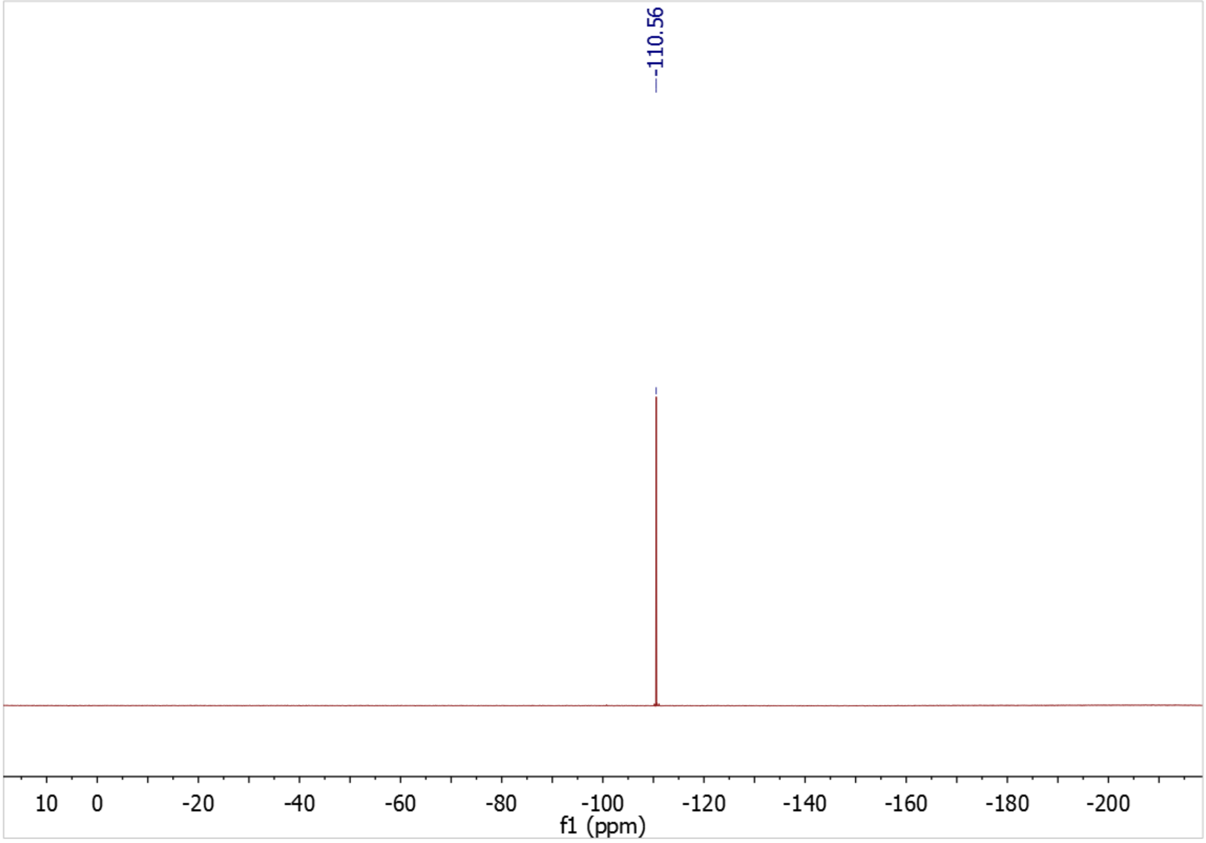

-110.56
10
0
-20
-40
-60
-80
-100
-120
-140
-160
-180
-200
f1 (ppm)

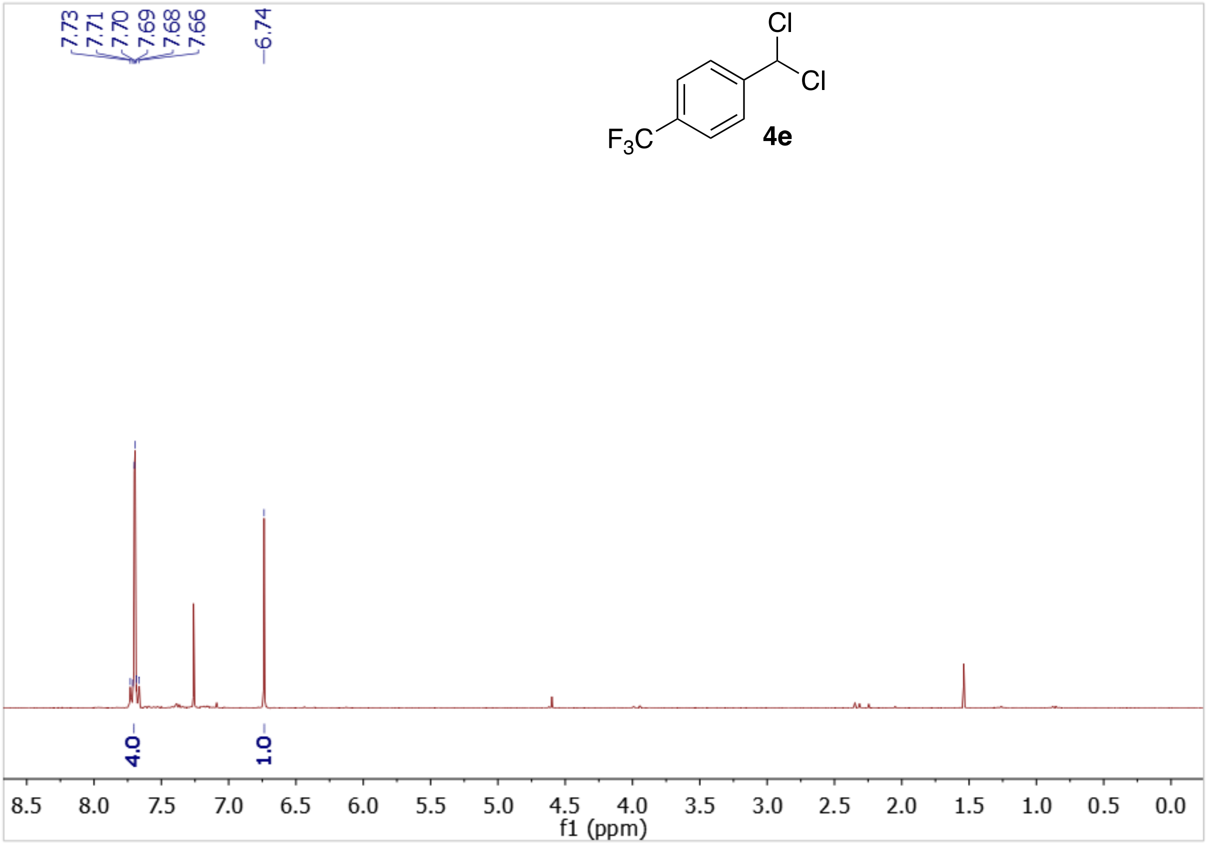
7.73
7.71
7.70
7.69
7.68
7.66
6.74
Cl
Cl
F3C
4e
4.0
1.0
8.5 8.0 7.5 7.0 6.5 6.0 5.5 5.0 4.5 4.0 3.5 3.0 2.5 2.0 1.5 1.0 0.5 0.0
f1 (ppm)

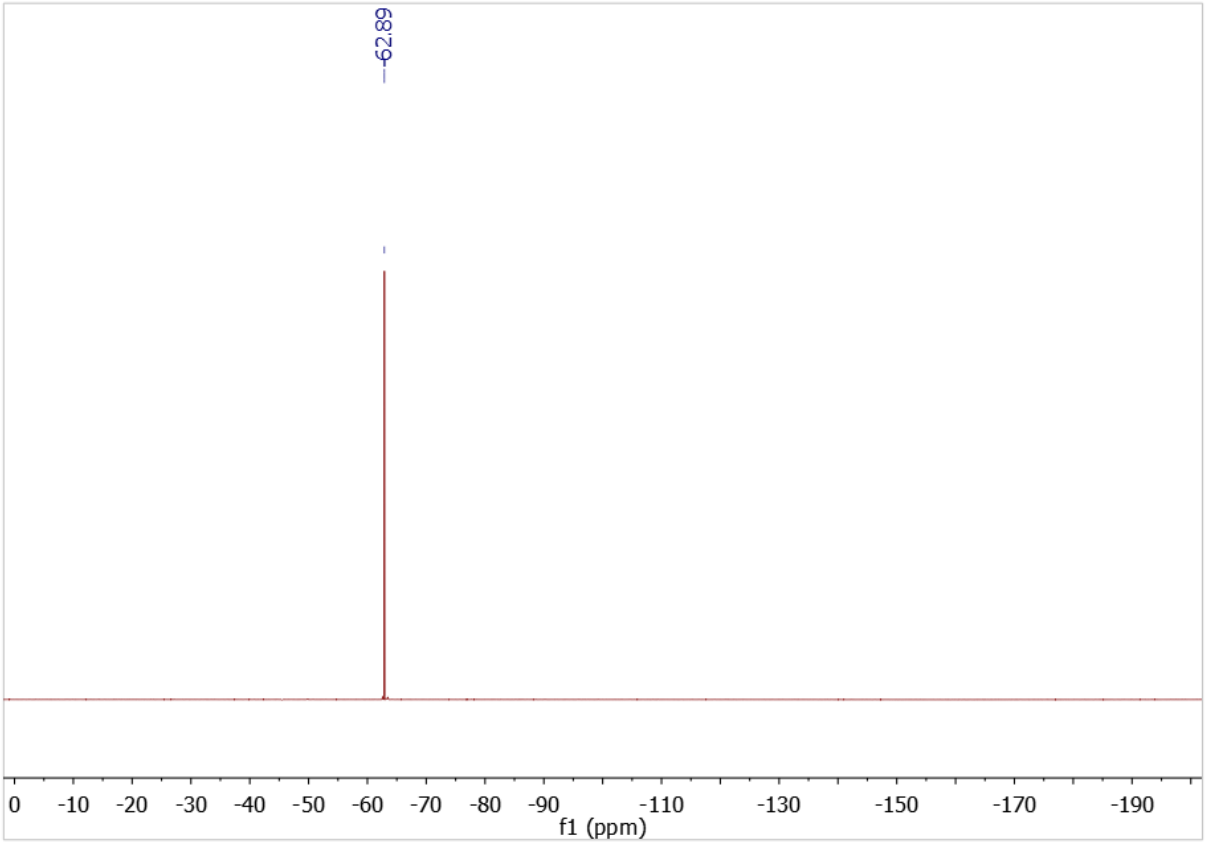
-62.89
0 -10 -20 -30 -40 -50 -60 -70 -80 -90 -110 -130 -150 -170 -190
f1 (ppm)

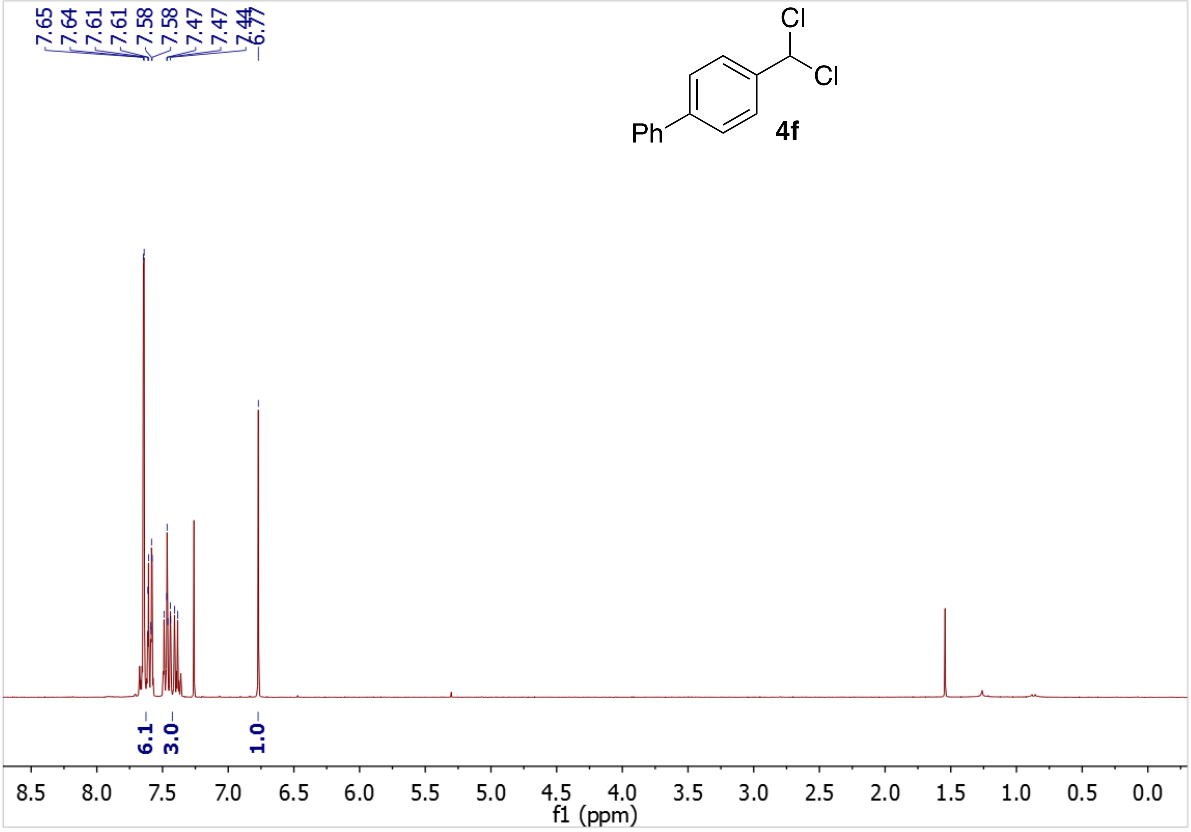
Cl
Cl
Ph
4f
7.65
7.64
7.61
7.61
7.58
7.58
7.47
7.47
7.44
6.77
6.1
3.0
1.0
8.5 8.0 7.5 7.0 6.5 6.0 5.5 5.0 4.5 4.0 3.5 3.0 2.5 2.0 1.5 1.0 0.5 0.0
f1 (ppm)

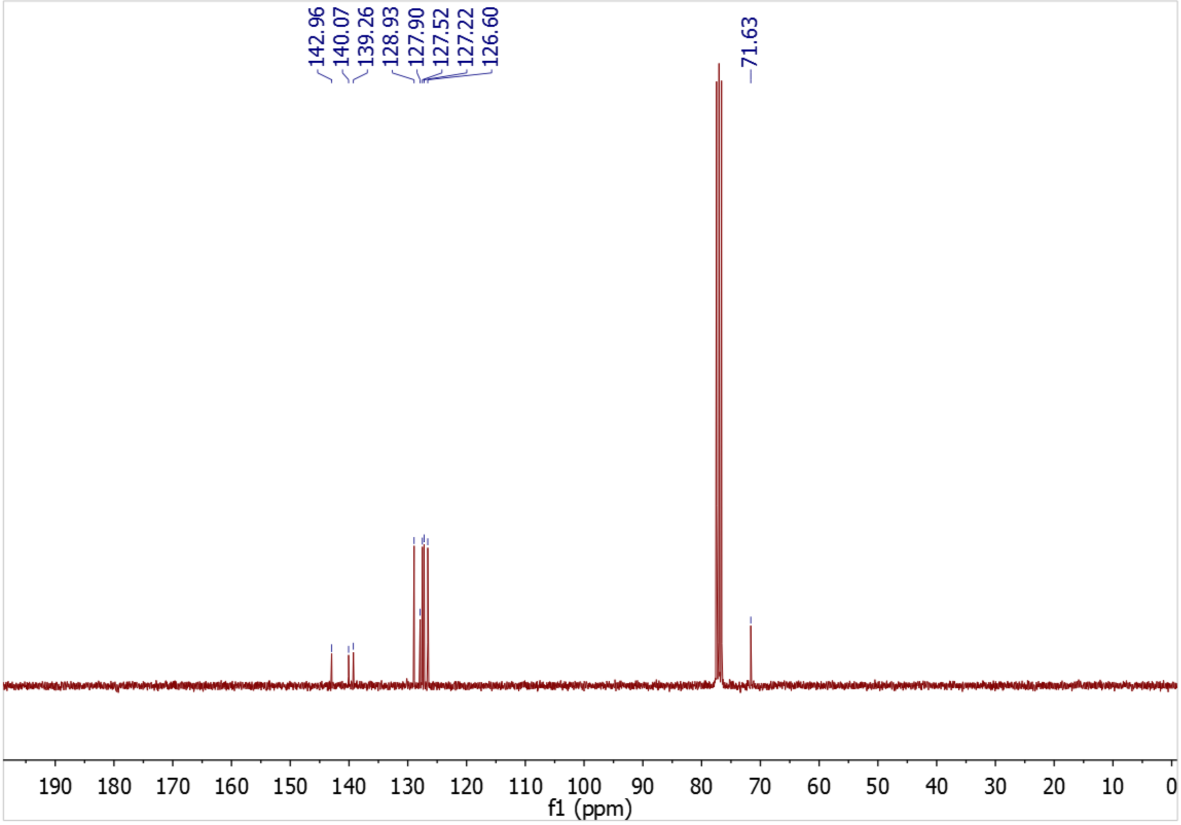
142.96
140.07
139.26
128.93
127.90
127.52
127.22
126.60
71.63
190 180 170 160 150 140 130 120 110 100 90 80 70 60 50 40 30 20 10 0
f1 (ppm)

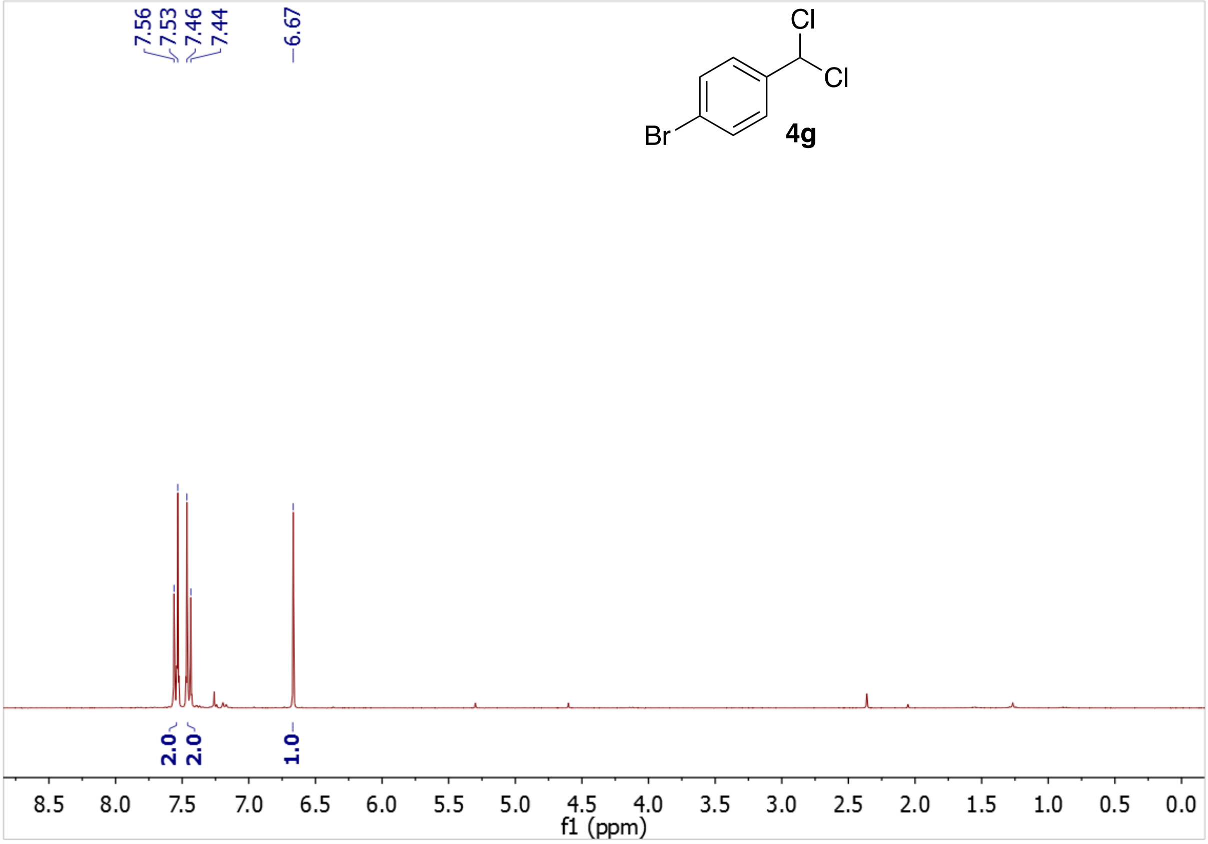
7.56
7.53
7.46
7.44
6.67
Cl
Cl
Br
4g
2.0
2.0
1.0
8.5 8.0 7.5 7.0 6.5 6.0 5.5 5.0 4.5 4.0 3.5 3.0 2.5 2.0 1.5 1.0 0.5 0.0
f1 (ppm)

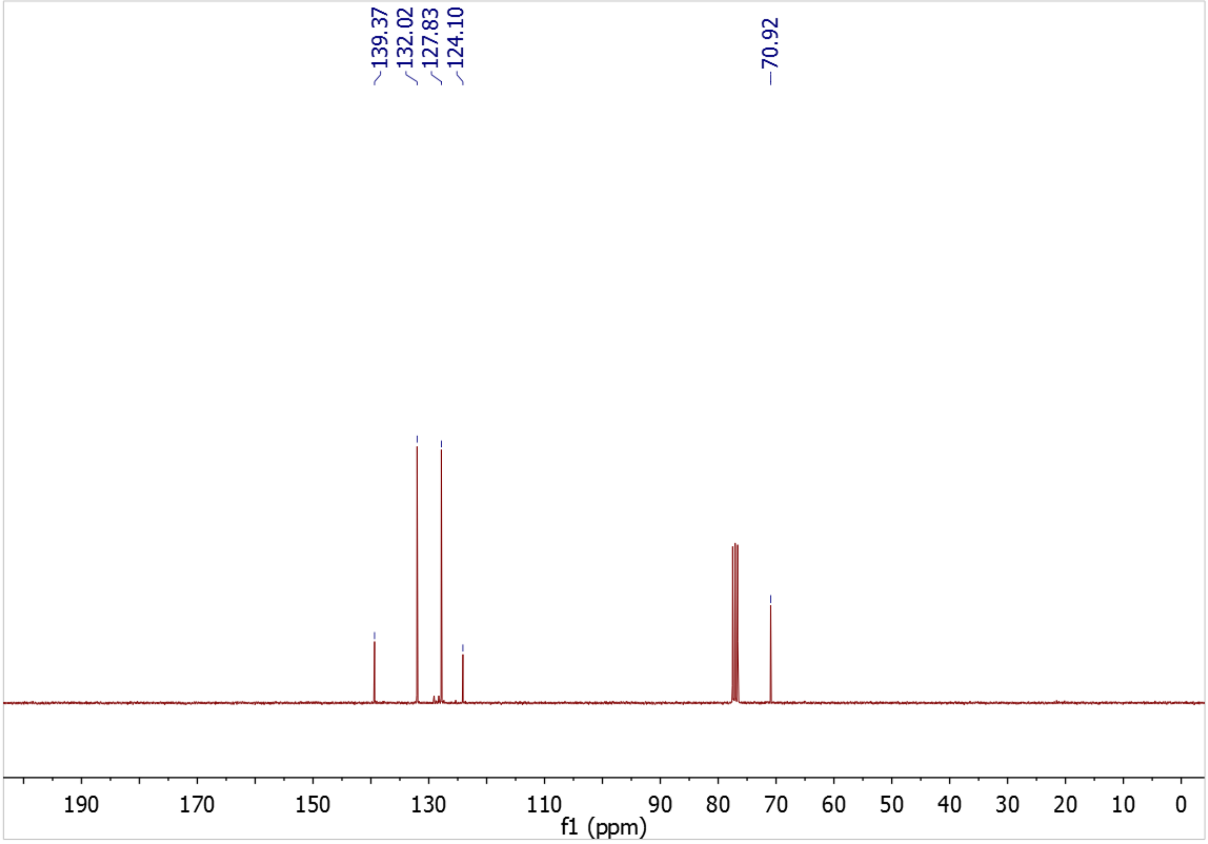
139.37
132.02
127.83
124.10
70.92
190 170 150 130 110 90 80 70 60 50 40 30 20 10 0
f1 (ppm)

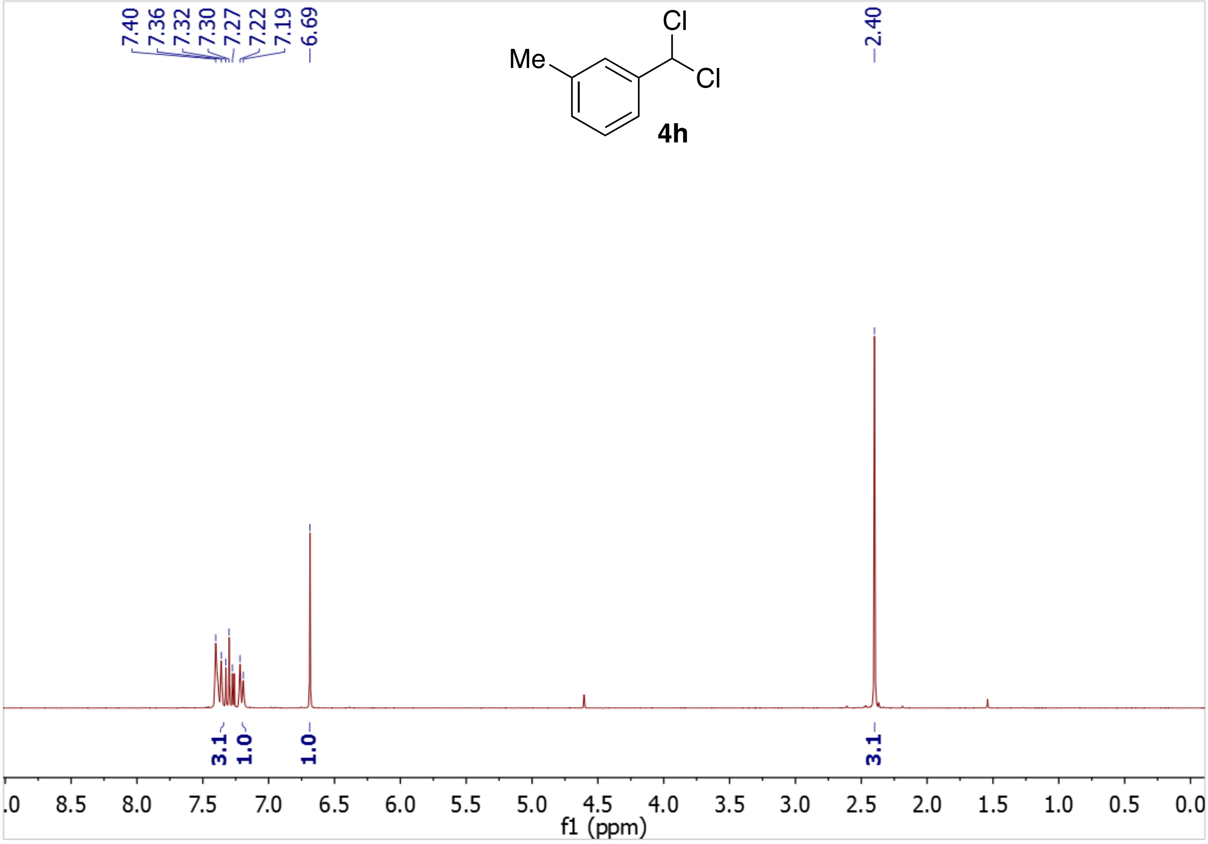
7.40
7.36
7.32
7.30
7.27
7.22
7.19
6.69
Me
Cl
Cl
4h
2.40
3.1
1.0
1.0
3.1
f1 (ppm)

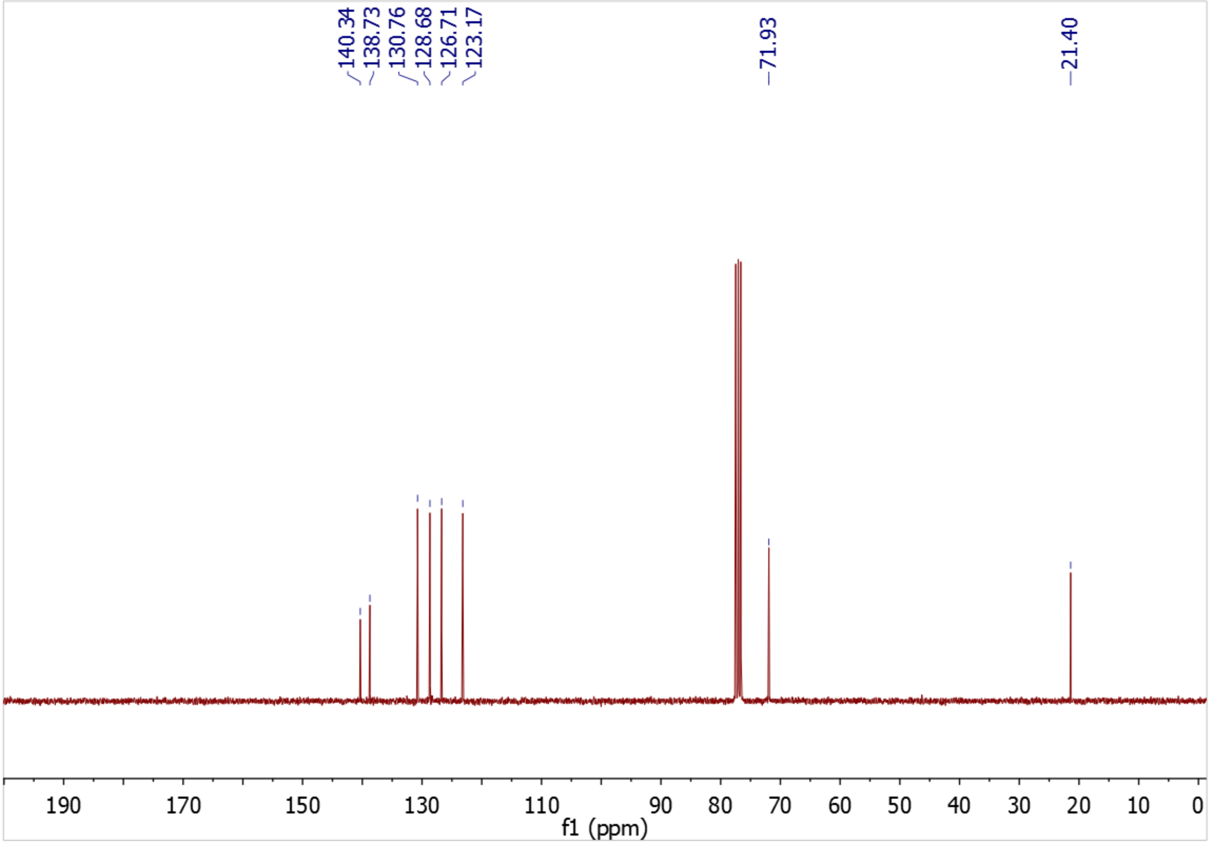
140.34
138.73
130.76
128.68
126.71
123.17
71.93
21.40
f1 (ppm)

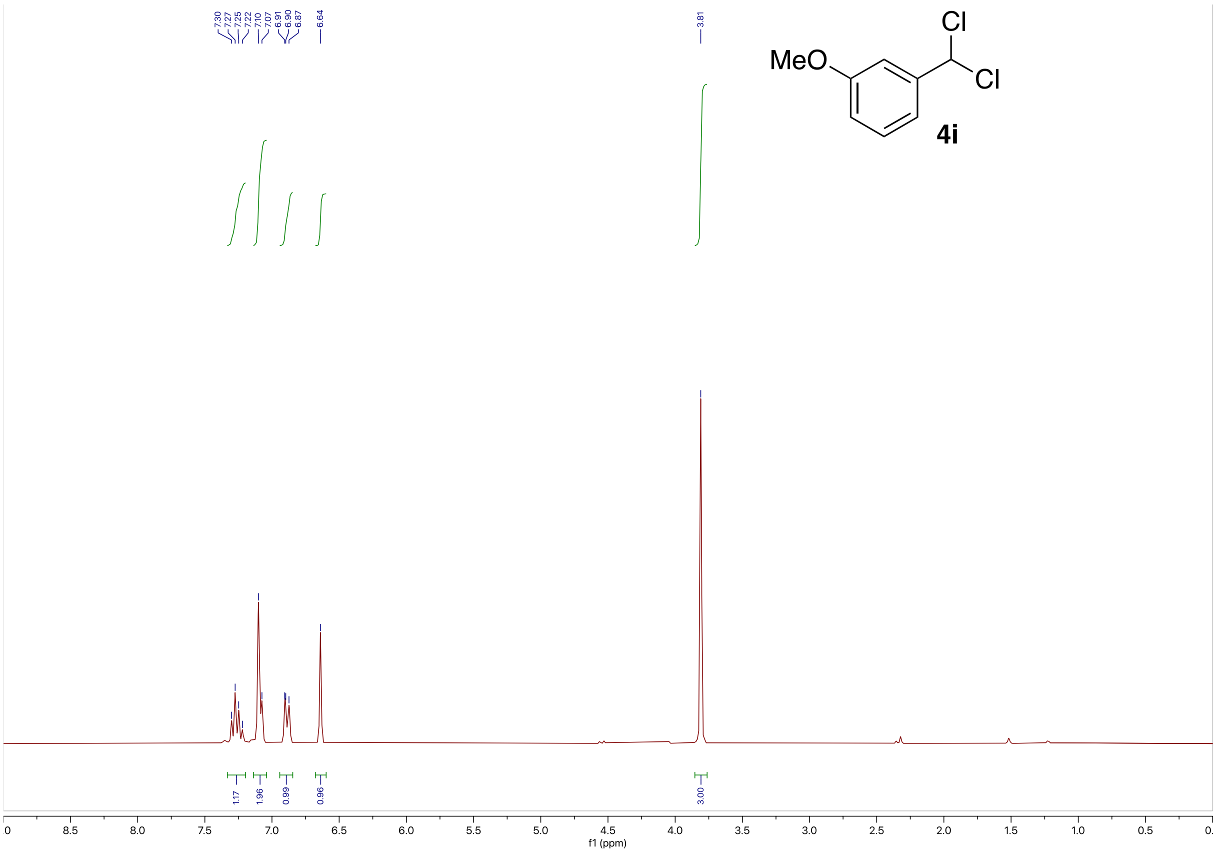

7.30
7.27
7.25
7.22
7.10
7.07
6.91
6.90
6.87
6.64
3.81
MeO
Cl
Cl
4i
1.17
1.96
0.99
0.96
3.00
f1 (ppm)

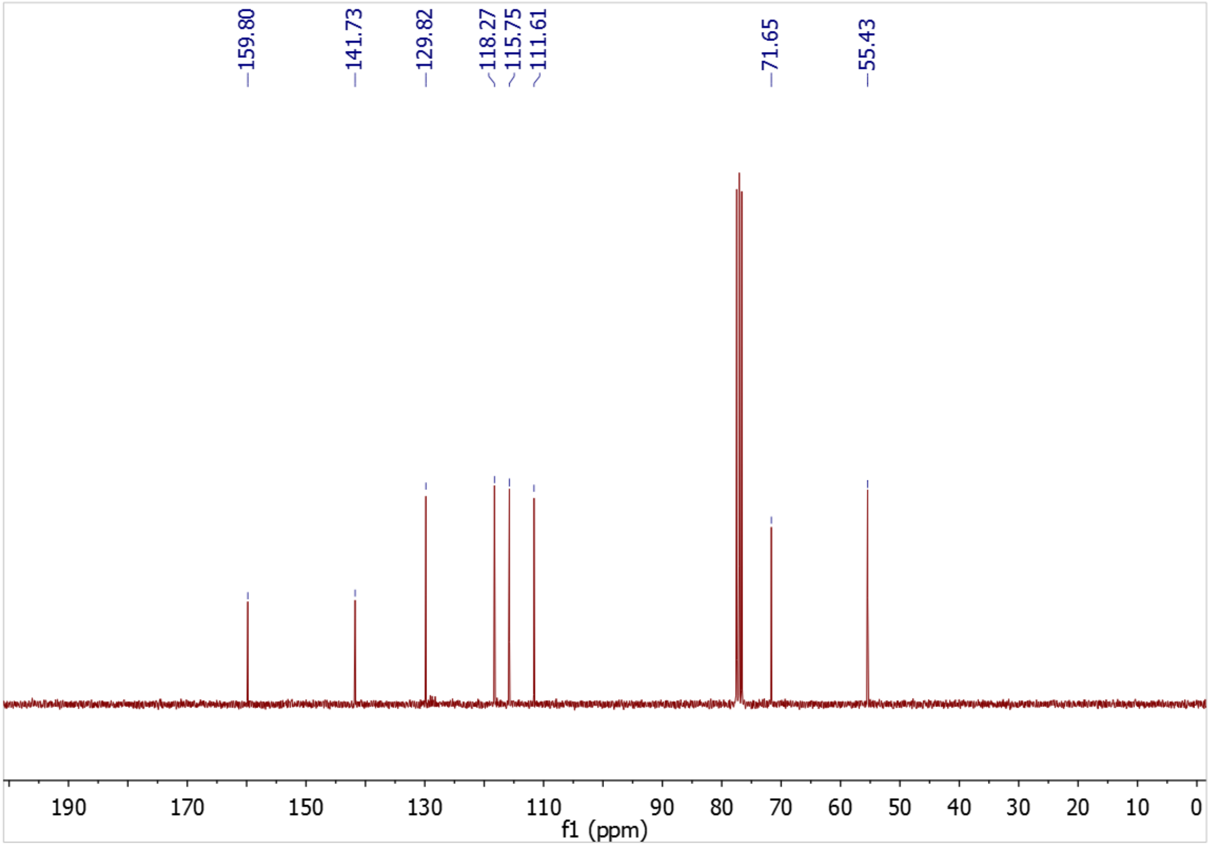

159.80
141.73
129.82
118.27
115.75
111.61
71.65
55.43
f1 (ppm)

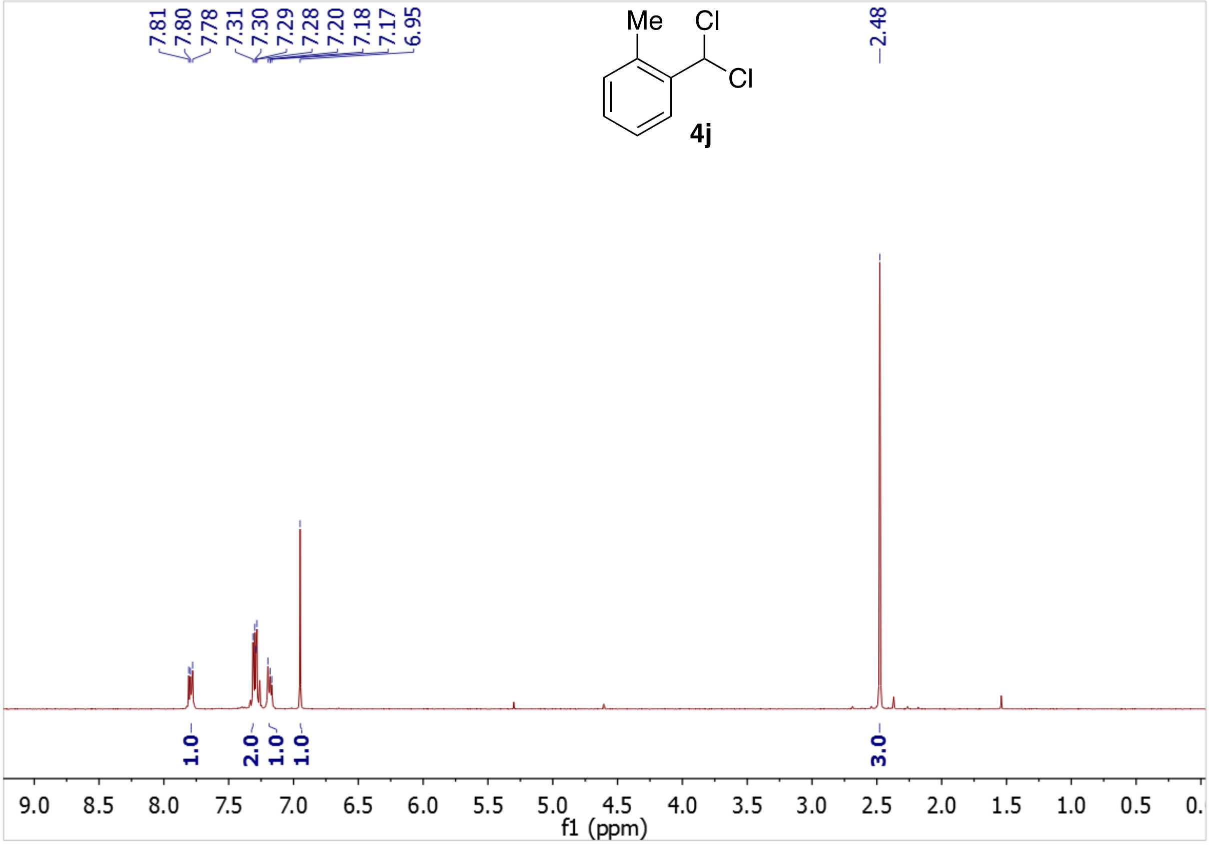
7.81
7.80
7.78
7.31
7.30
7.29
7.28
7.20
7.18
7.17
6.95
2.48
Me
Cl
Cl
4j
1.0
2.0
1.0
1.0
3.0
9.0 8.5 8.0 7.5 7.0 6.5 6.0 5.5 5.0 4.5 4.0 3.5 3.0 2.5 2.0 1.5 1.0 0.5 0.
f1 (ppm)

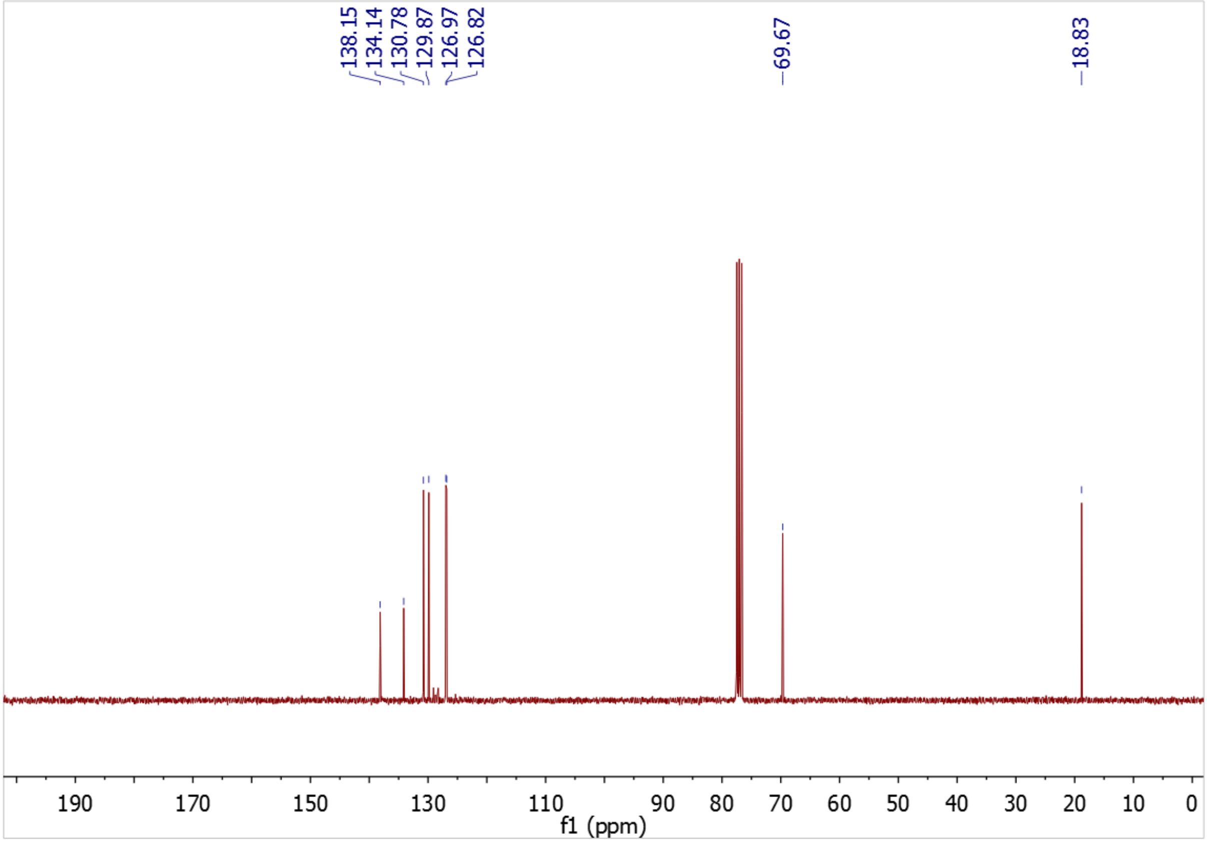
138.15
134.14
130.78
129.87
126.97
126.82
69.67
18.83
190 170 150 130 110 90 80 70 60 50 40 30 20 10 0
f1 (ppm)

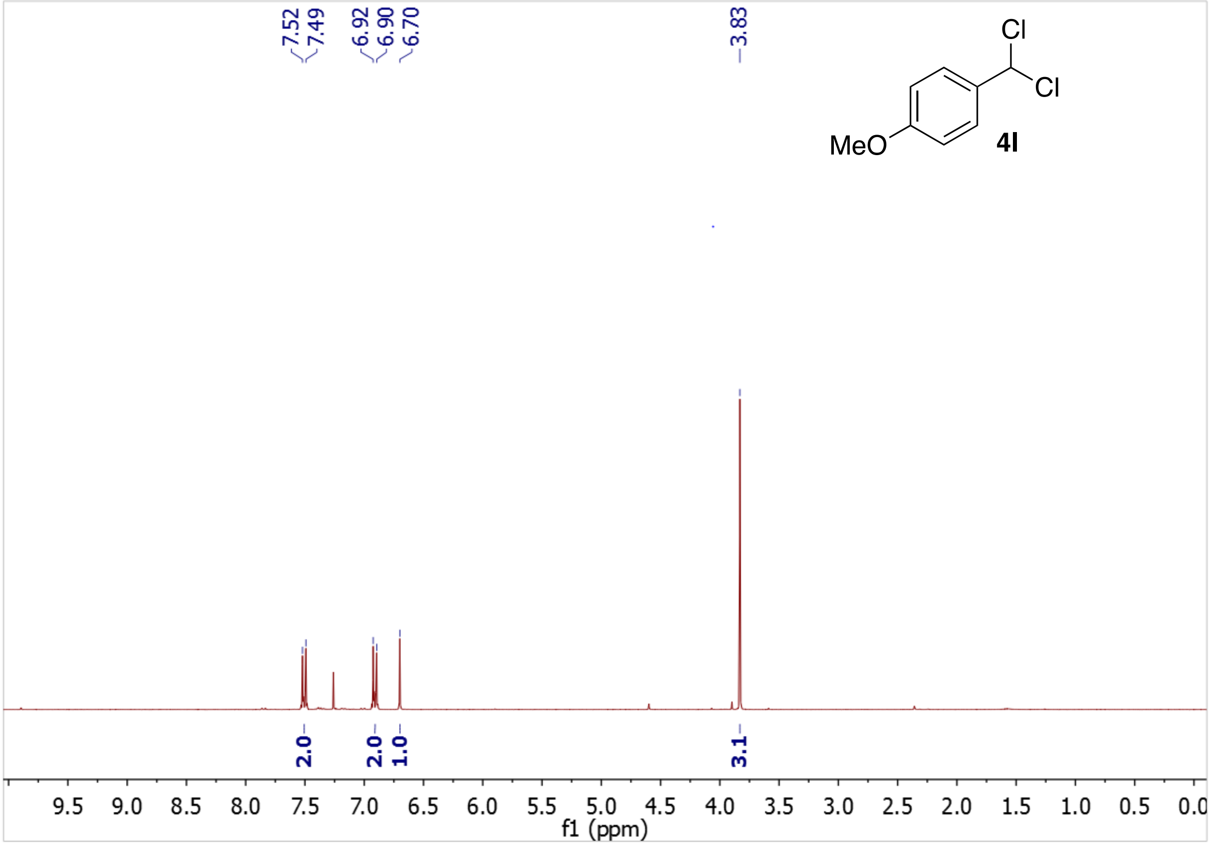
7.52
7.49
6.92
6.90
6.70
3.83
Cl
Cl
MeO
4l
2.0
2.0
1.0
3.1
9.5 9.0 8.5 8.0 7.5 7.0 6.5 6.0 5.5 5.0 4.5 4.0 3.5 3.0 2.5 2.0 1.5 1.0 0.5 0.0
f1 (ppm)

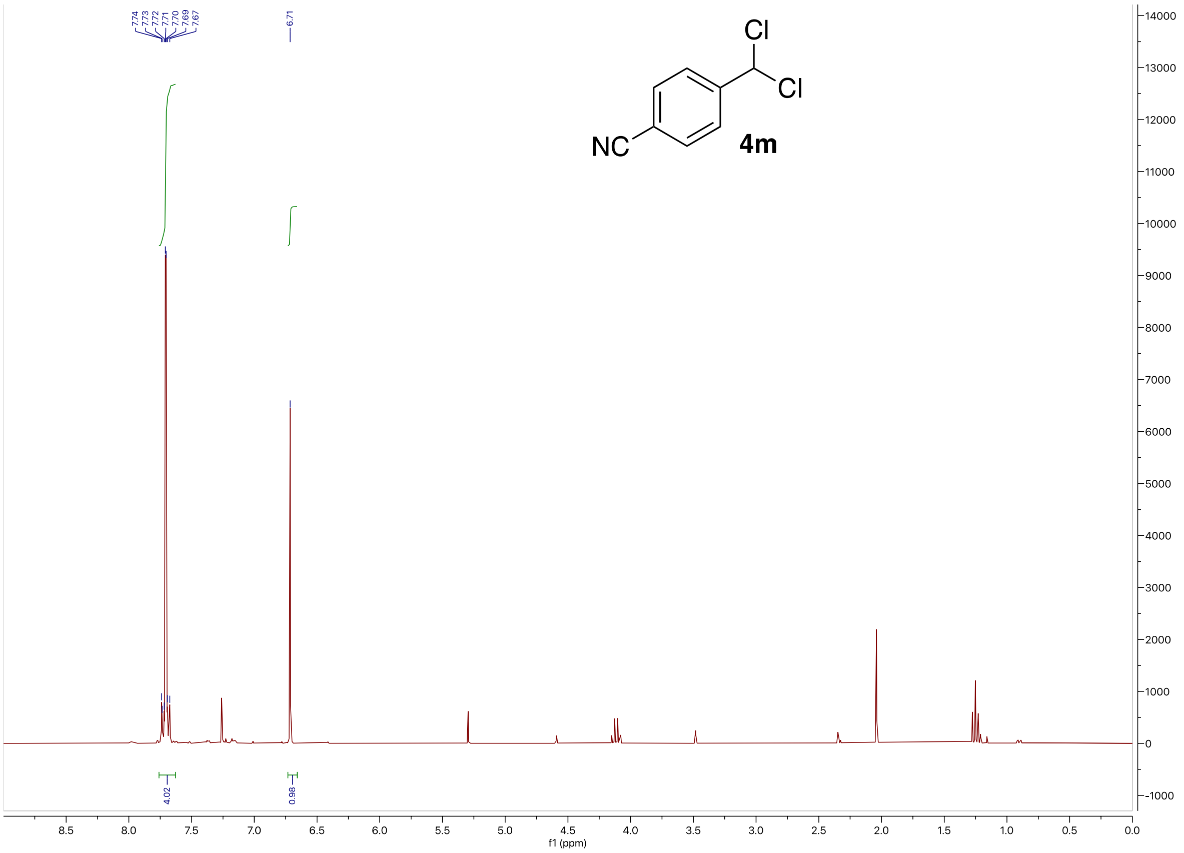
7.74
7.73
7.72
7.71
7.70
7.69
7.67
6.71
Cl
Cl
NC
4m
4.02
0.98
f1 (ppm)

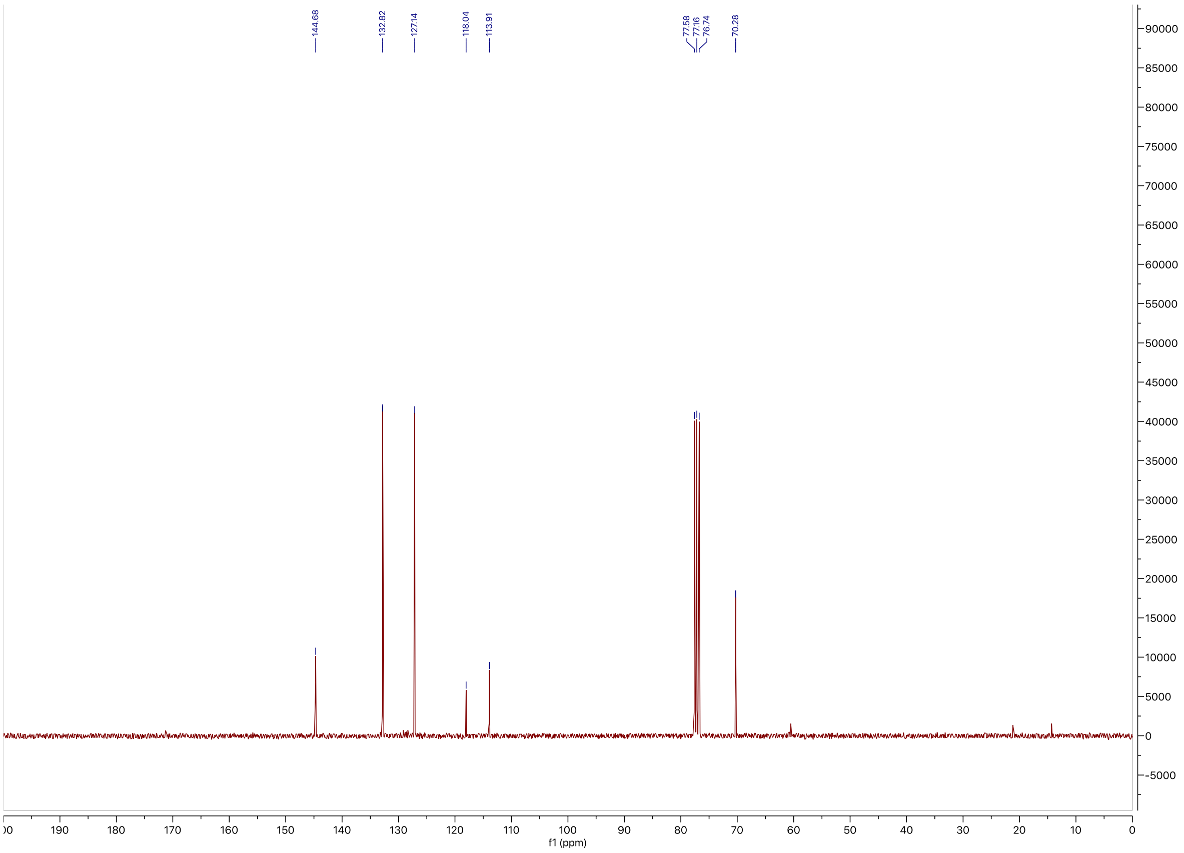
144.68
132.82
127.14
118.04
113.91
77.58
77.16
76.74
70.28
f1 (ppm)

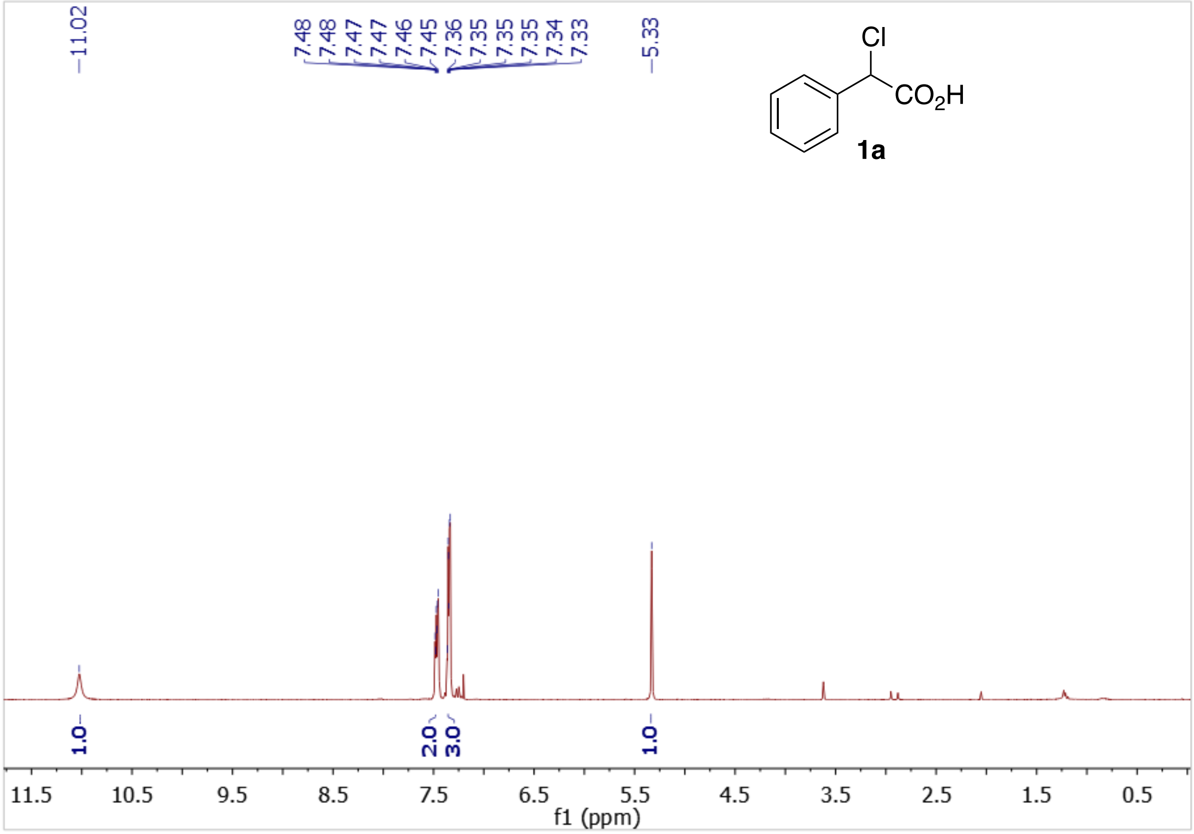
11.02
7.48
7.48
7.47
7.47
7.46
7.45
7.36
7.35
7.35
7.35
7.34
7.33
5.33
Cl
CO2H
1a
1.0
2.0
3.0
1.0
11.5
10.5
9.5
8.5
7.5
6.5
5.5
4.5
3.5
2.5
1.5
0.5
f1 (ppm)

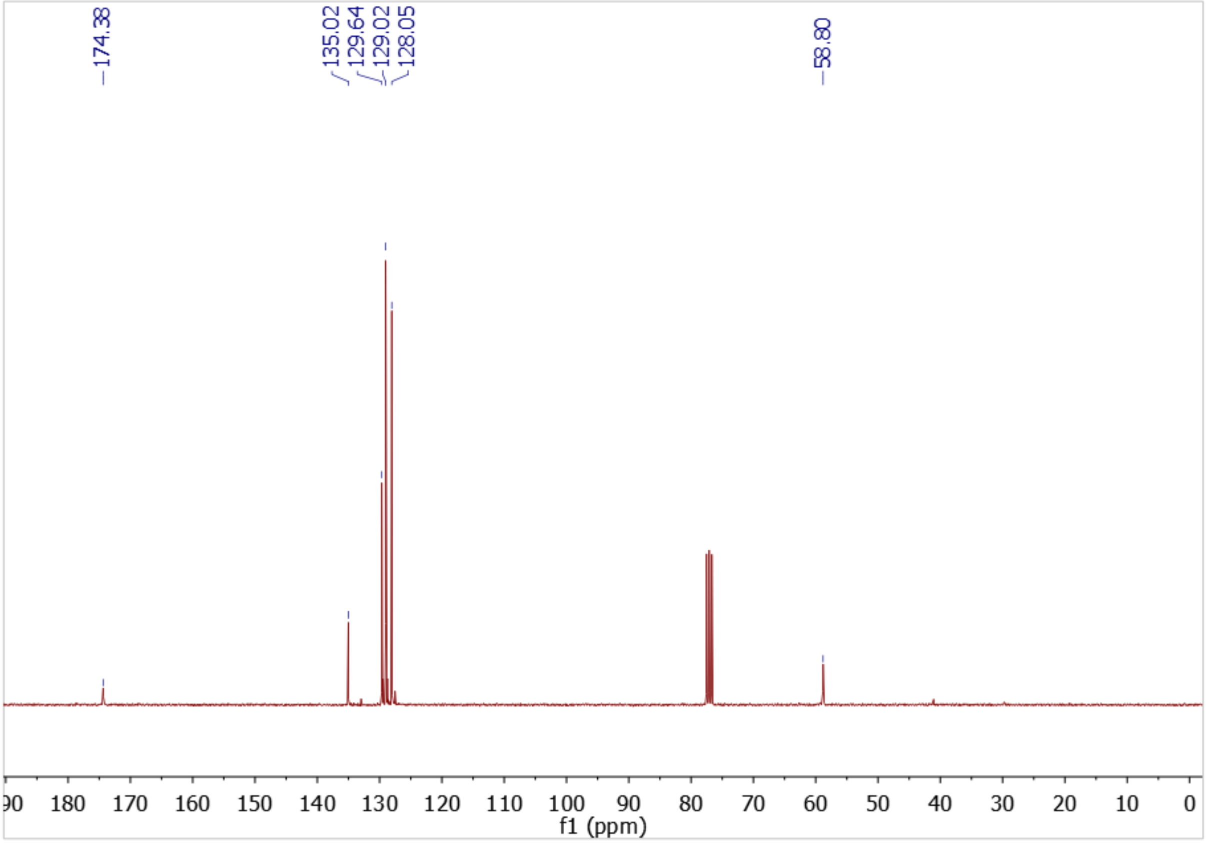
174.38
135.02
129.64
129.02
128.05
58.80
180
170
160
150
140
130
120
110
100
90
80
70
60
50
40
30
20
10
0
f1 (ppm)

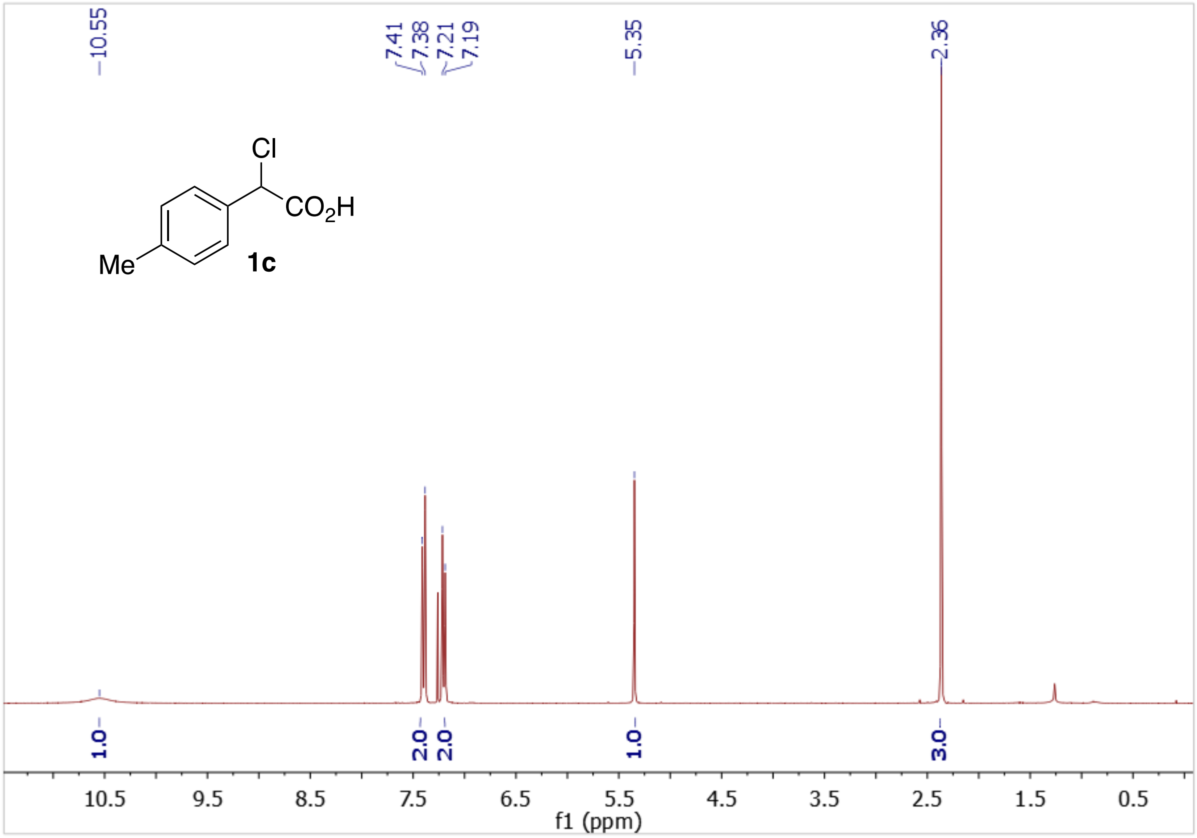

10.55
7.41
7.38
7.21
7.19
5.35
2.36
Cl
CO2H
Me
1c
1.0
2.0
2.0
1.0
3.0
10.5
9.5
8.5
7.5
6.5
5.5
4.5
3.5
2.5
1.5
0.5
f1 (ppm)

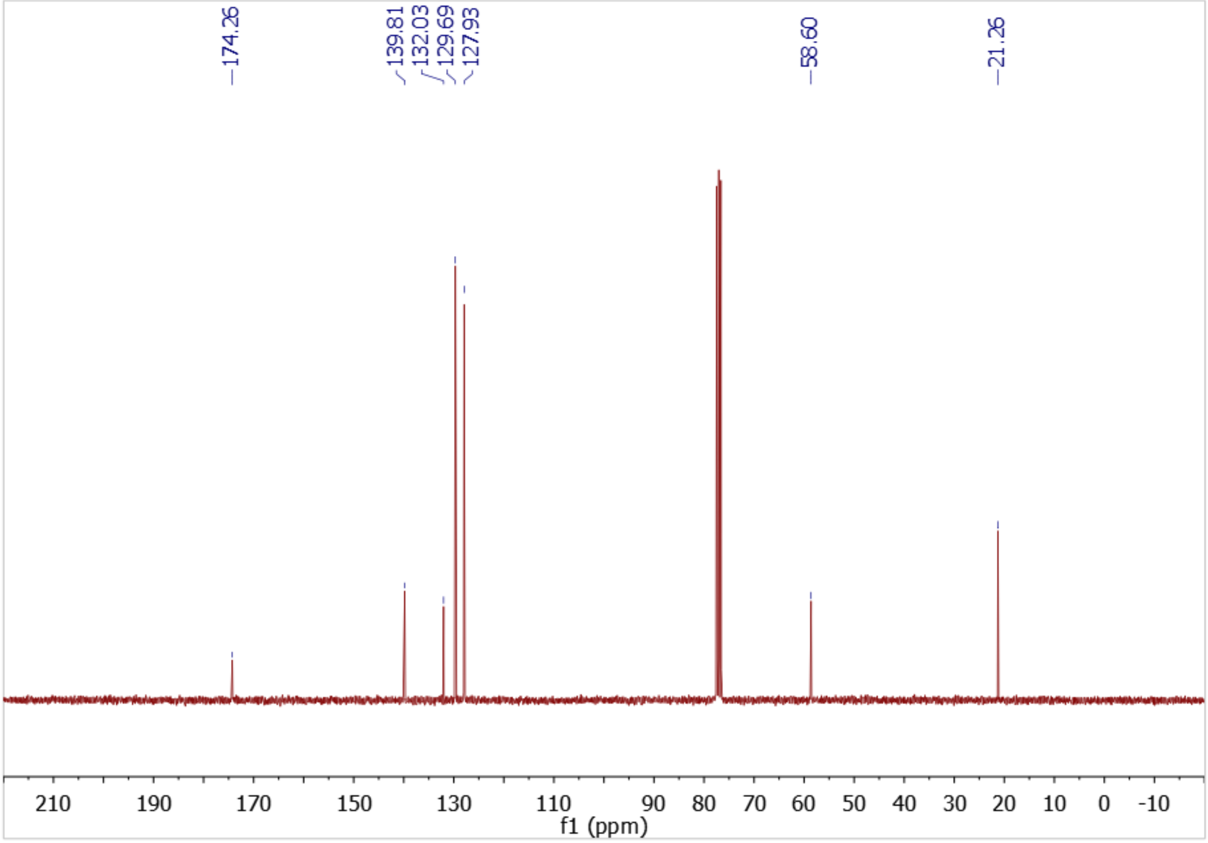

174.26
139.81
132.03
129.69
127.93
58.60
21.26
210
190
170
150
130
110
90
80
70
60
50
40
30
20
10
0
-10
f1 (ppm)

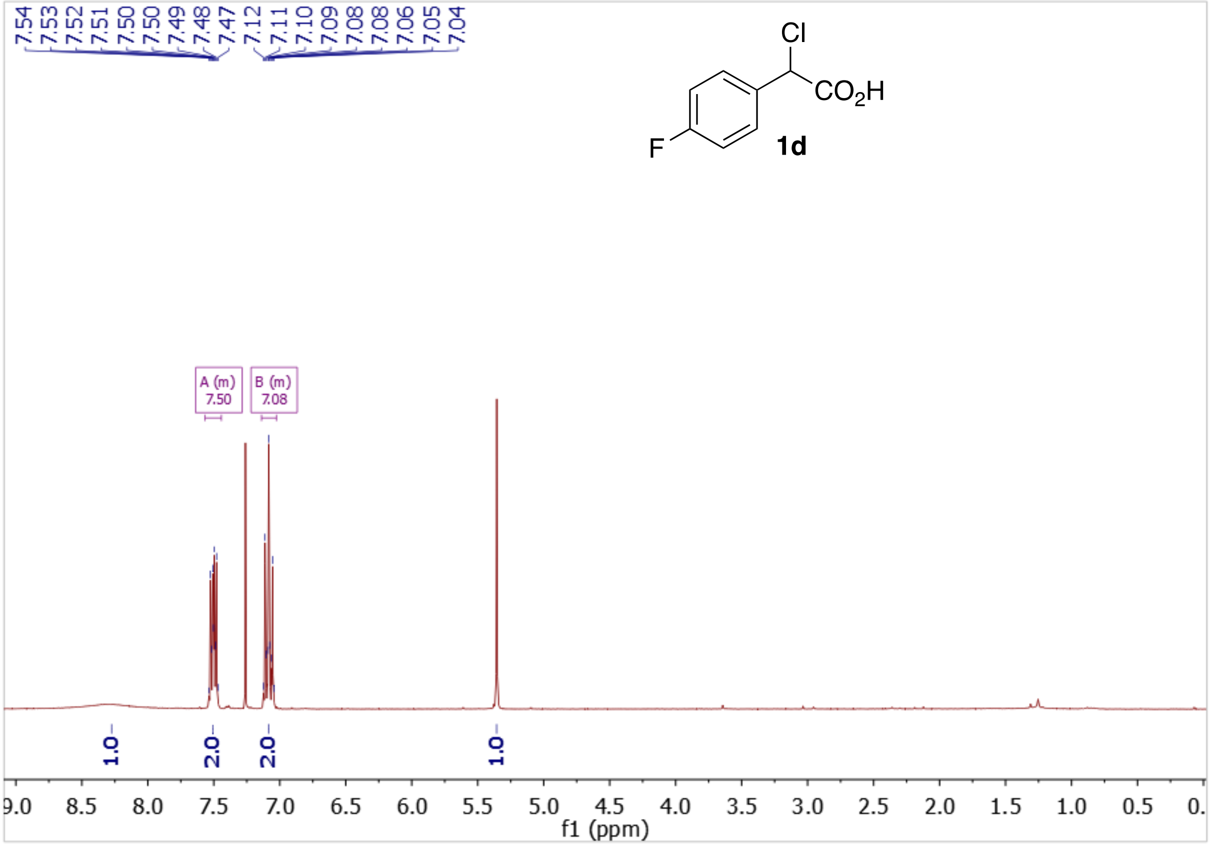
7.54 7.53 7.52 7.51 7.50 7.50 7.49 7.48 7.47 7.12 7.11 7.10 7.09 7.08 7.08 7.06 7.05 7.04
Cl
CO2H
F
1d
A (m) 7.50
B (m) 7.08
1.0
2.0
2.0
1.0
9.0 8.5 8.0 7.5 7.0 6.5 6.0 5.5 5.0 4.5 4.0 3.5 3.0 2.5 2.0 1.5 1.0 0.5 0.
f1 (ppm)

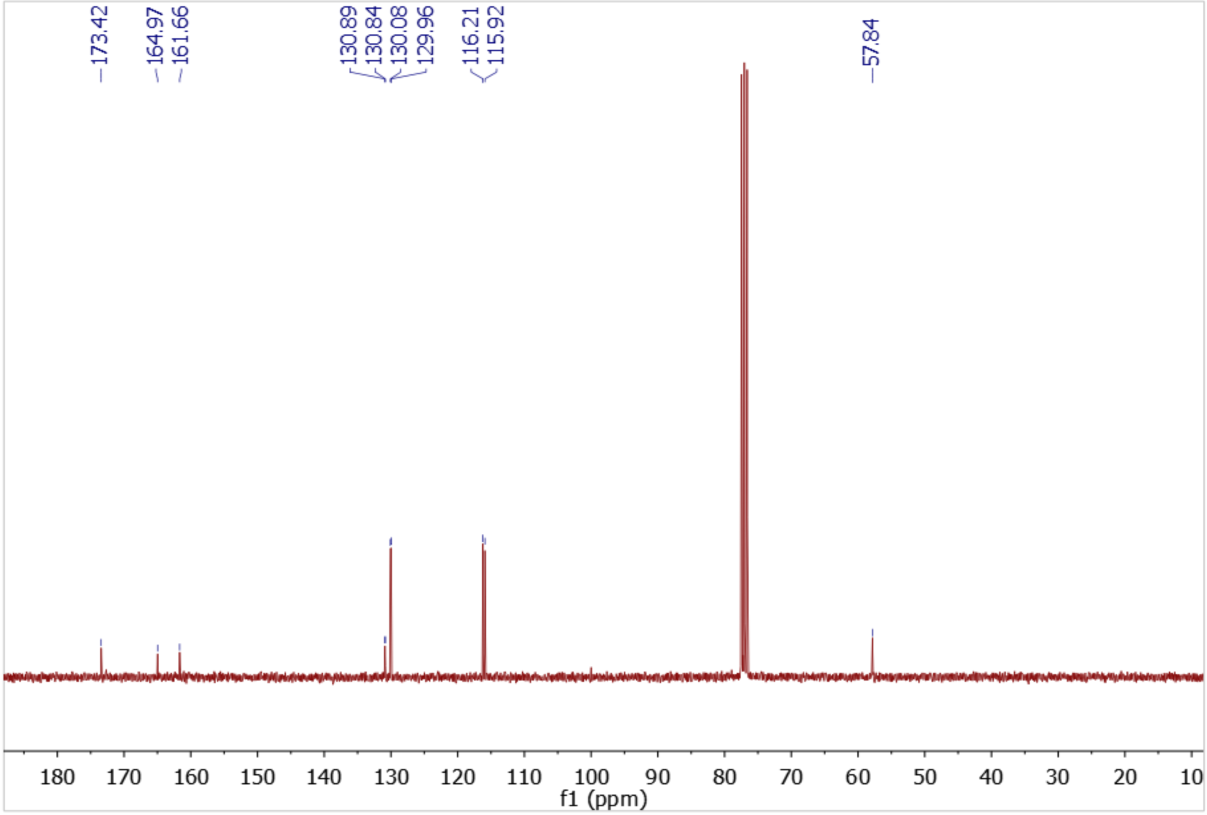
173.42
164.97
161.66
130.89 130.84 130.08 129.96
116.21 115.92
57.84
180 170 160 150 140 130 120 110 100 90 80 70 60 50 40 30 20 10
f1 (ppm)

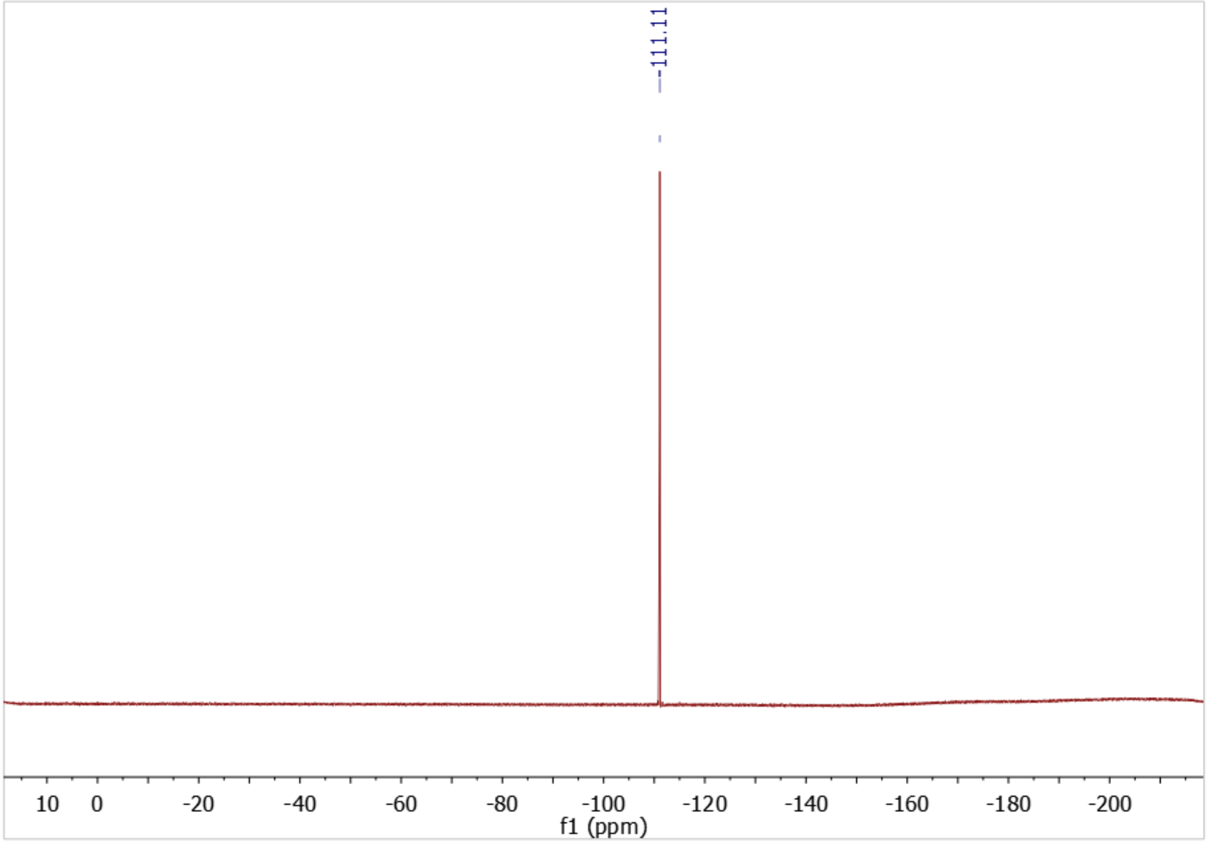

-111.11
10
0
-20
-40
-60
-80
-100
-120
-140
-160
-180
-200
f1 (ppm)

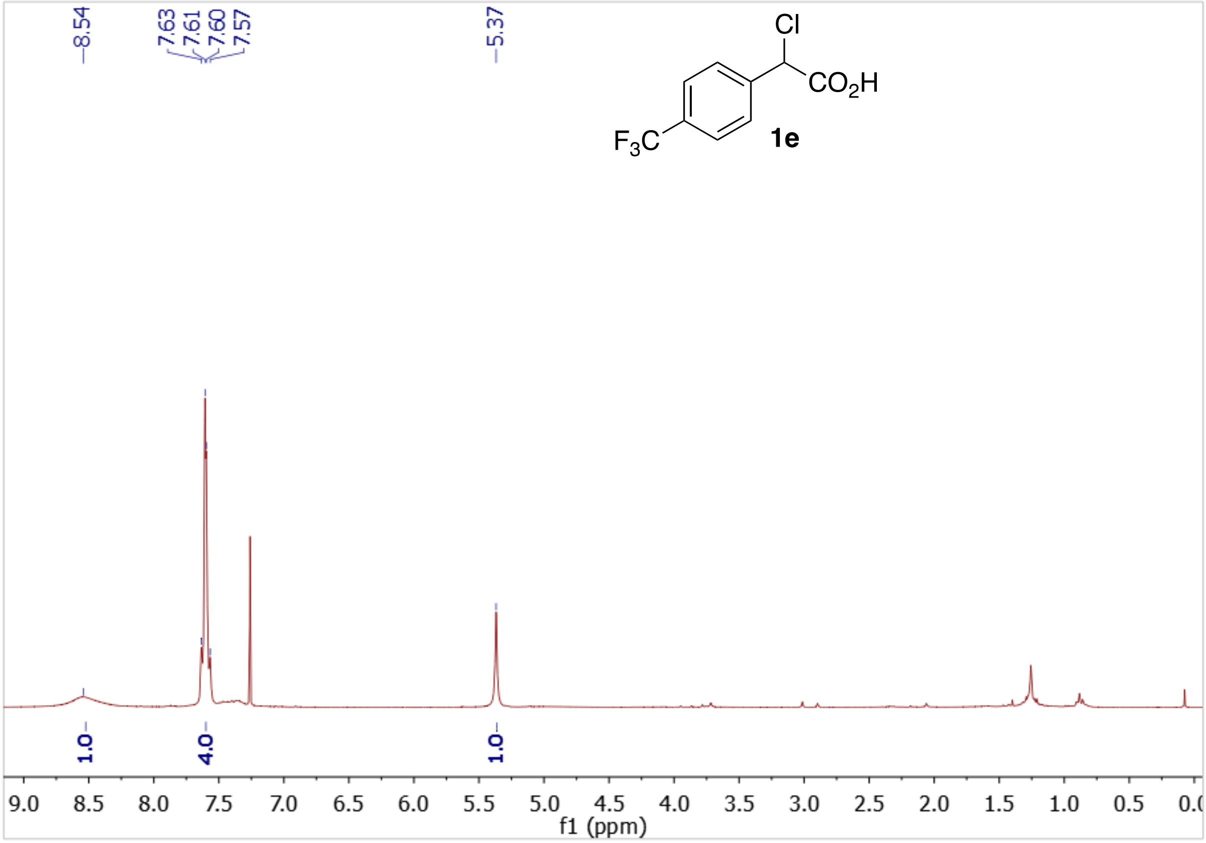
8.54
7.63
7.61
7.60
7.57
5.37
Cl
CO2H
F3C
1e
1.0
4.0
1.0
9.0 8.5 8.0 7.5 7.0 6.5 6.0 5.5 5.0 4.5 4.0 3.5 3.0 2.5 2.0 1.5 1.0 0.5
f1 (ppm)

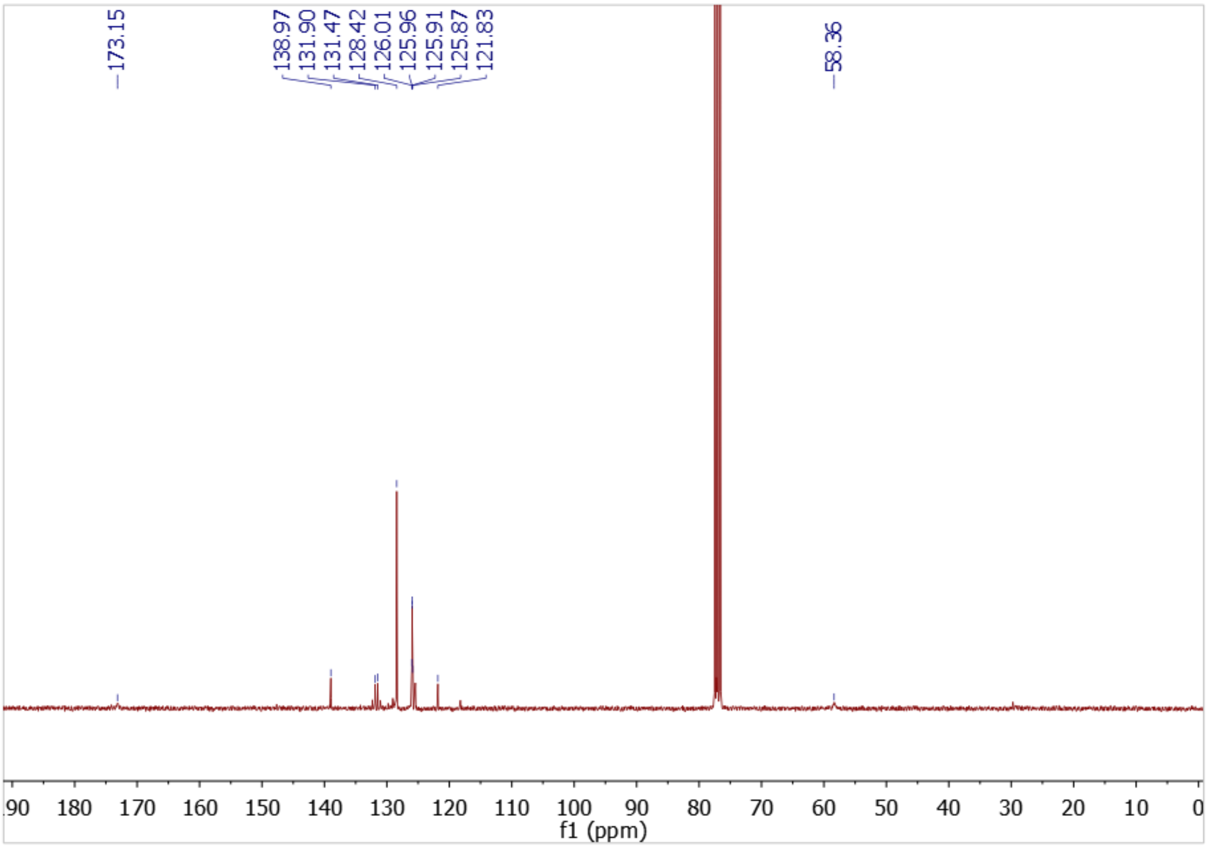
173.15
138.97
131.90
131.47
128.42
126.01
125.96
125.91
125.87
121.83
58.36
180 170 160 150 140 130 120 110 100 90 80 70 60 50 40 30 20 10 0
f1 (ppm)

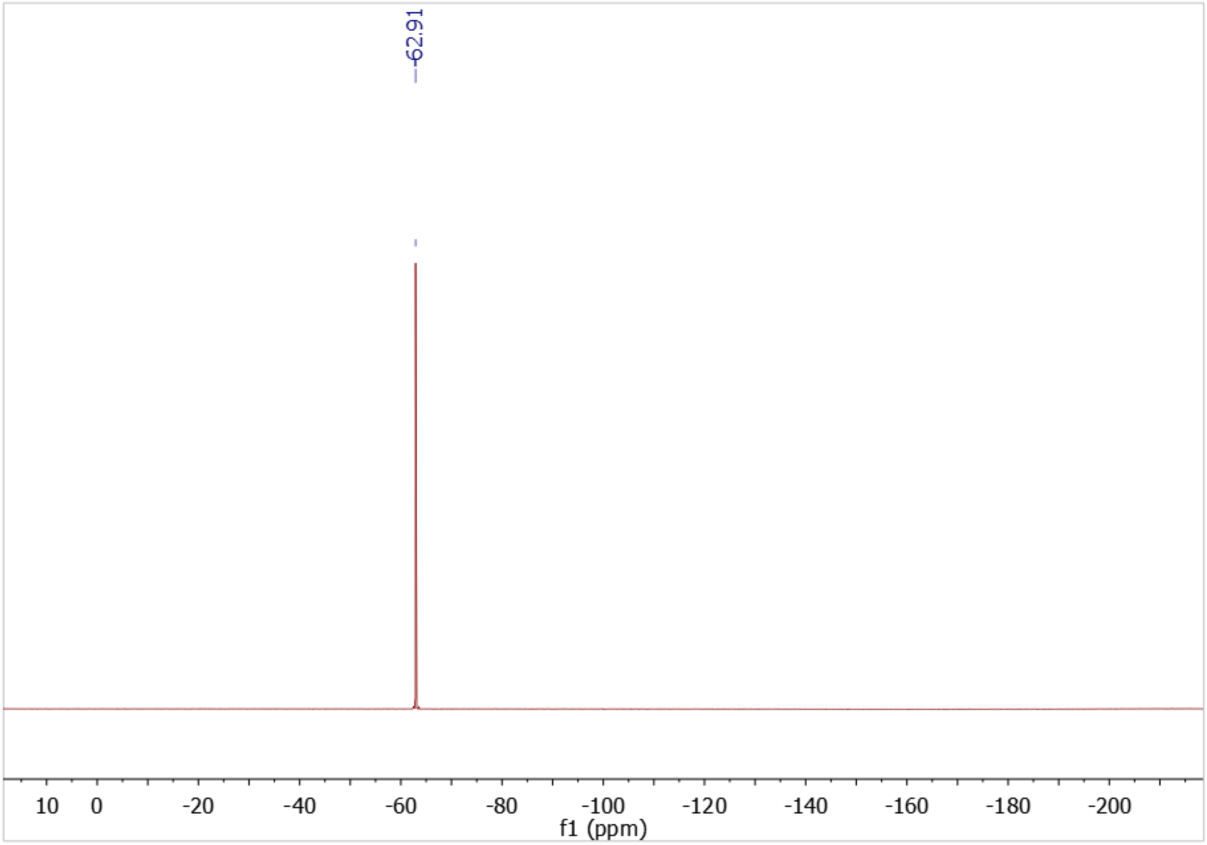

-62.91
10 0 -20 -40 -60 -80 -100 -120 -140 -160 -180 -200
f1 (ppm)

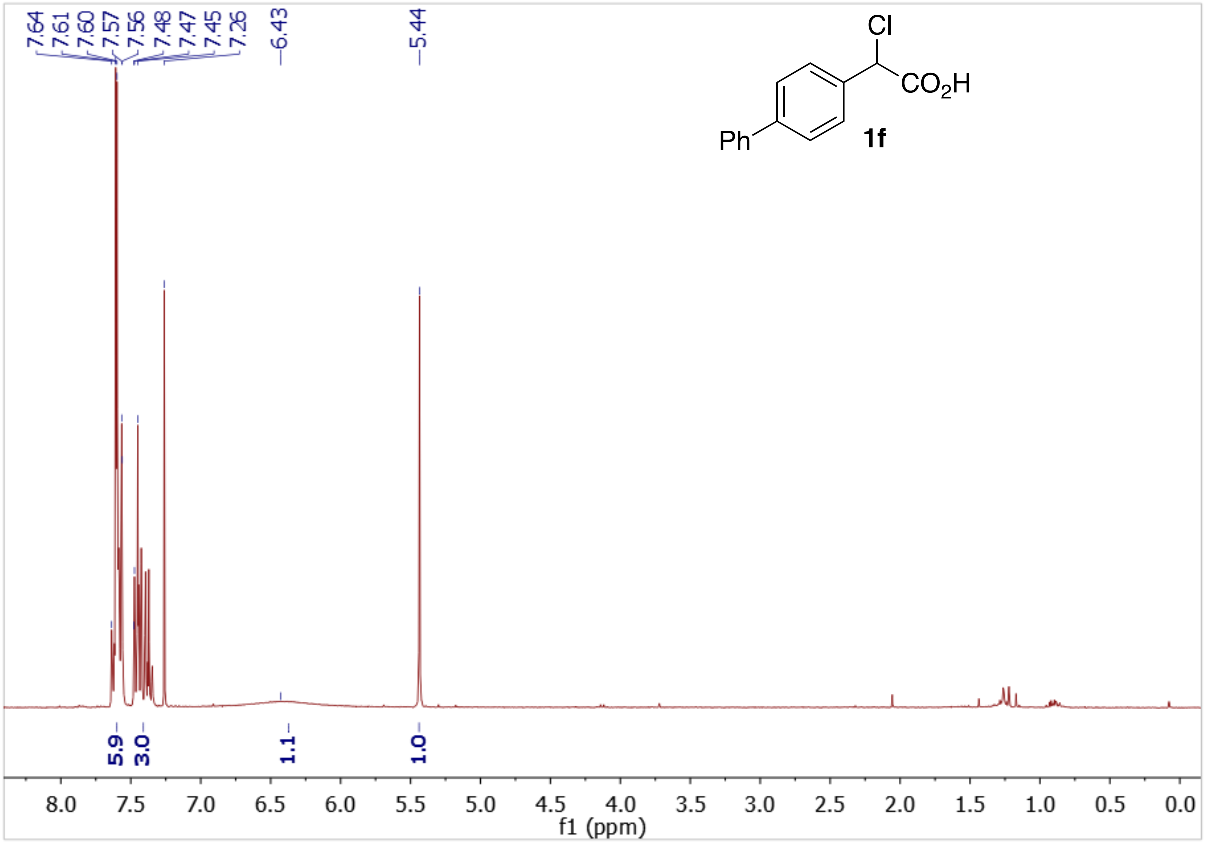
7.64
7.61
7.60
7.57
7.56
7.48
7.47
7.45
7.26
6.43
5.44
Cl
CO_2H
Ph
1f
5.9
3.0
1.1
1.0
8.0 7.5 7.0 6.5 6.0 5.5 5.0 4.5 4.0 3.5 3.0 2.5 2.0 1.5 1.0 0.5 0.0
f1 (ppm)

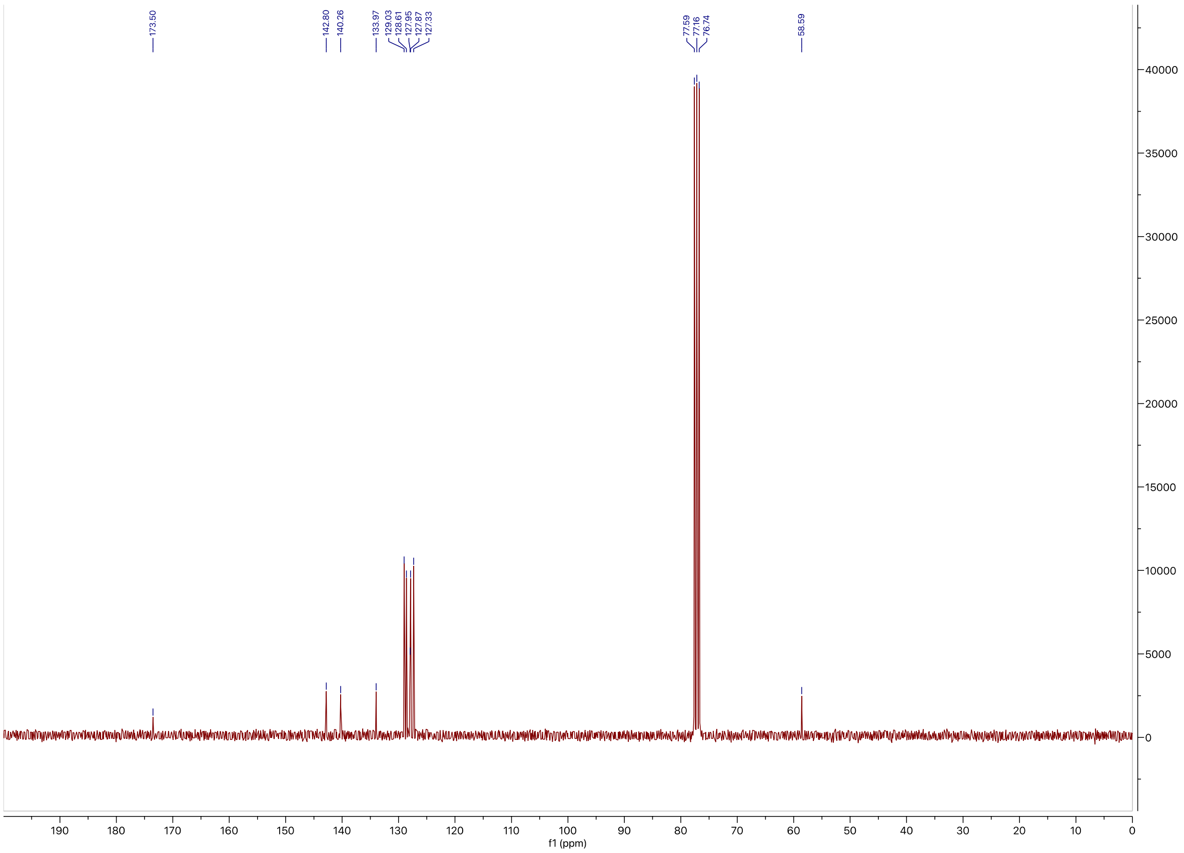
173.50
142.80
140.26
133.97
129.03
128.61
127.95
127.87
127.33
77.59
77.16
76.74
58.59
40000
35000
30000
25000
20000
15000
10000
5000
0
190 180 170 160 150 140 130 120 110 100 90 80 70 60 50 40 30 20 10 0
f1 (ppm)

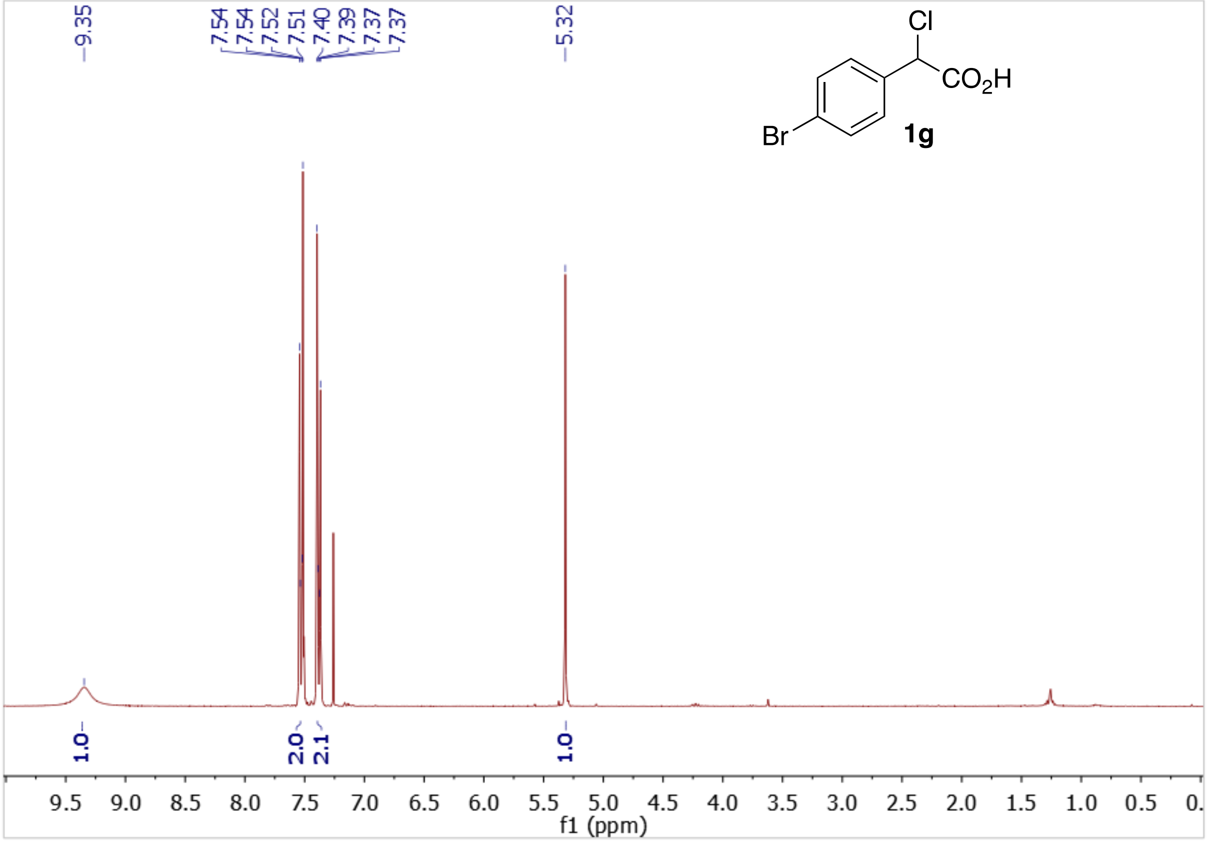
9.35
7.54
7.54
7.52
7.51
7.40
7.39
7.37
7.37
5.32
Cl
CO2H
Br
1g
1.0
2.0
2.1
1.0
9.5 9.0 8.5 8.0 7.5 7.0 6.5 6.0 5.5 5.0 4.5 4.0 3.5 3.0 2.5 2.0 1.5 1.0 0.5 0.
f1 (ppm)

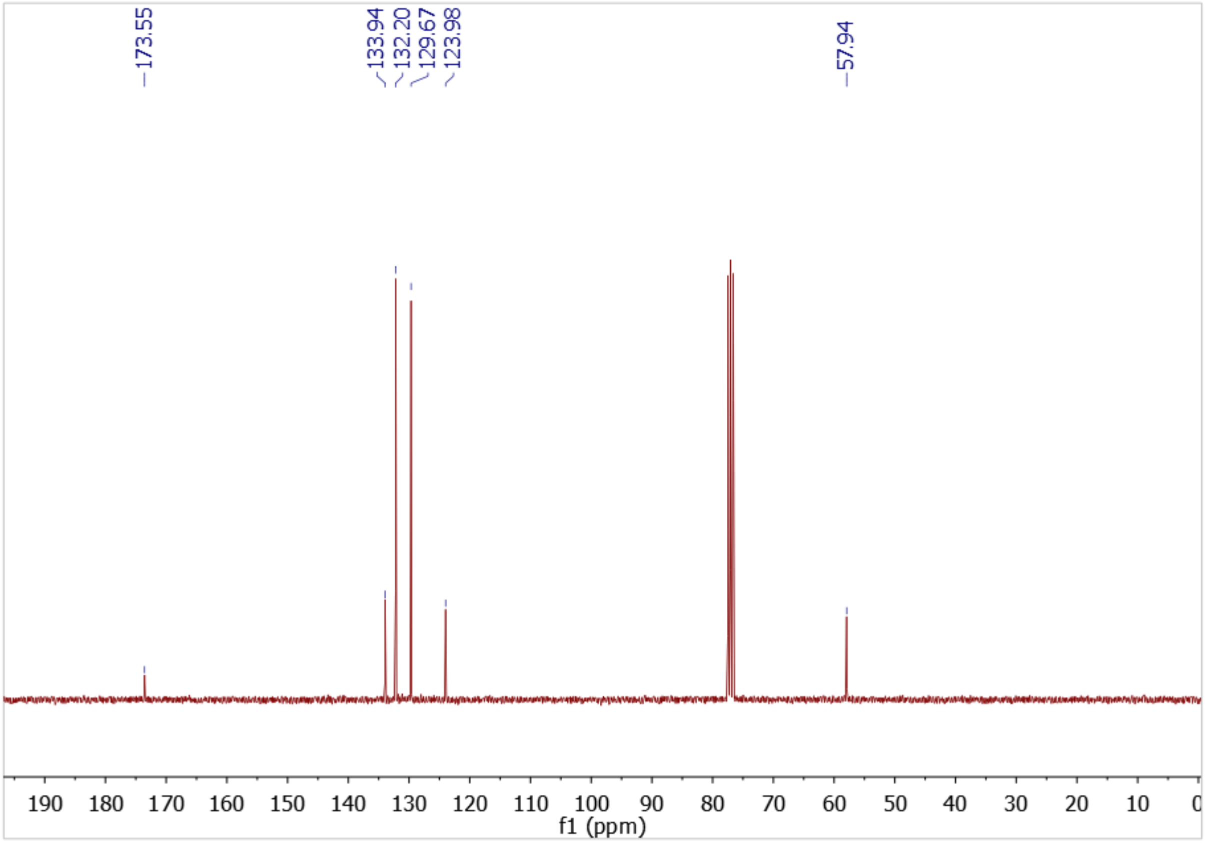
173.55
133.94
132.20
129.67
123.98
57.94
190 180 170 160 150 140 130 120 110 100 90 80 70 60 50 40 30 20 10 0
f1 (ppm)

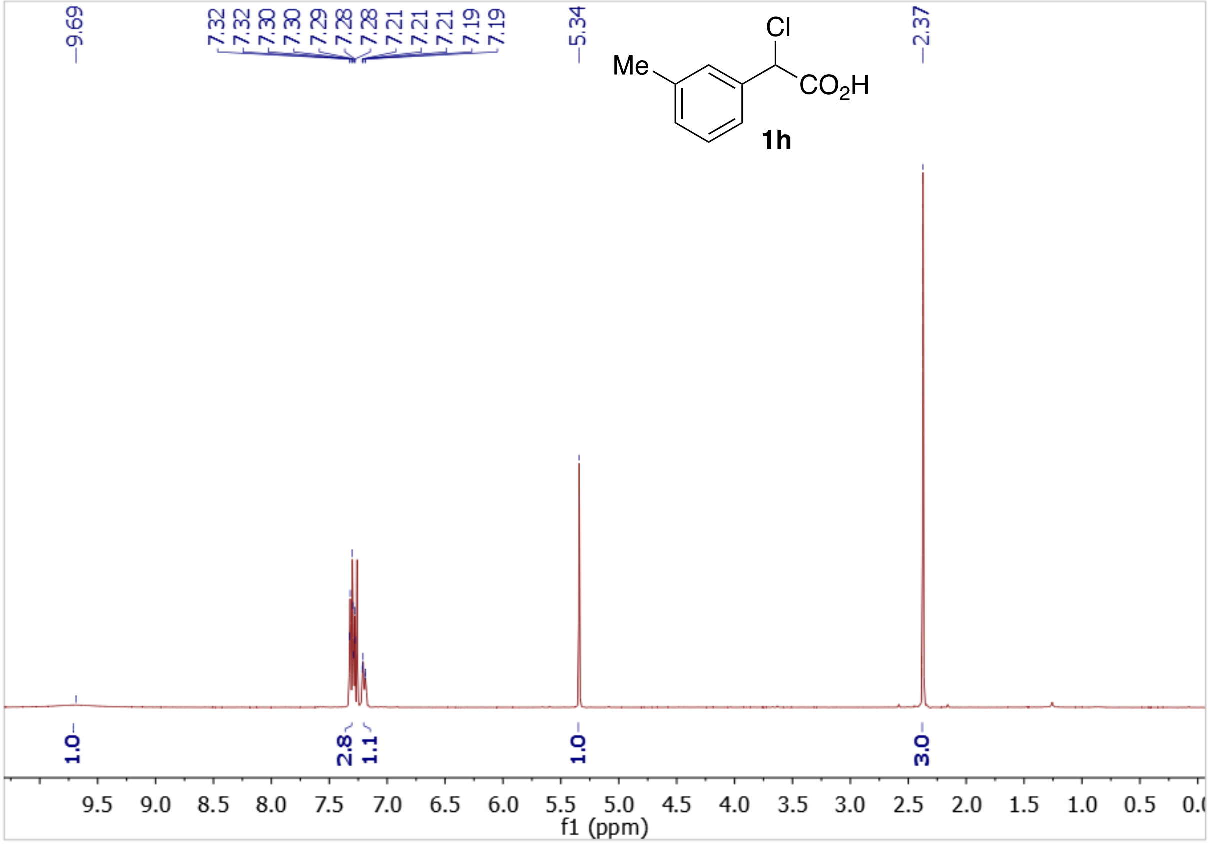
9.69
7.32 7.32 7.30 7.30 7.29 7.28 7.28 7.21 7.21 7.21 7.19 7.19
5.34
2.37
Cl
Me
CO2H
1h
1.0
2.8
1.1
1.0
3.0
9.5 9.0 8.5 8.0 7.5 7.0 6.5 6.0 5.5 5.0 4.5 4.0 3.5 3.0 2.5 2.0 1.5 1.0 0.5
f1 (ppm)

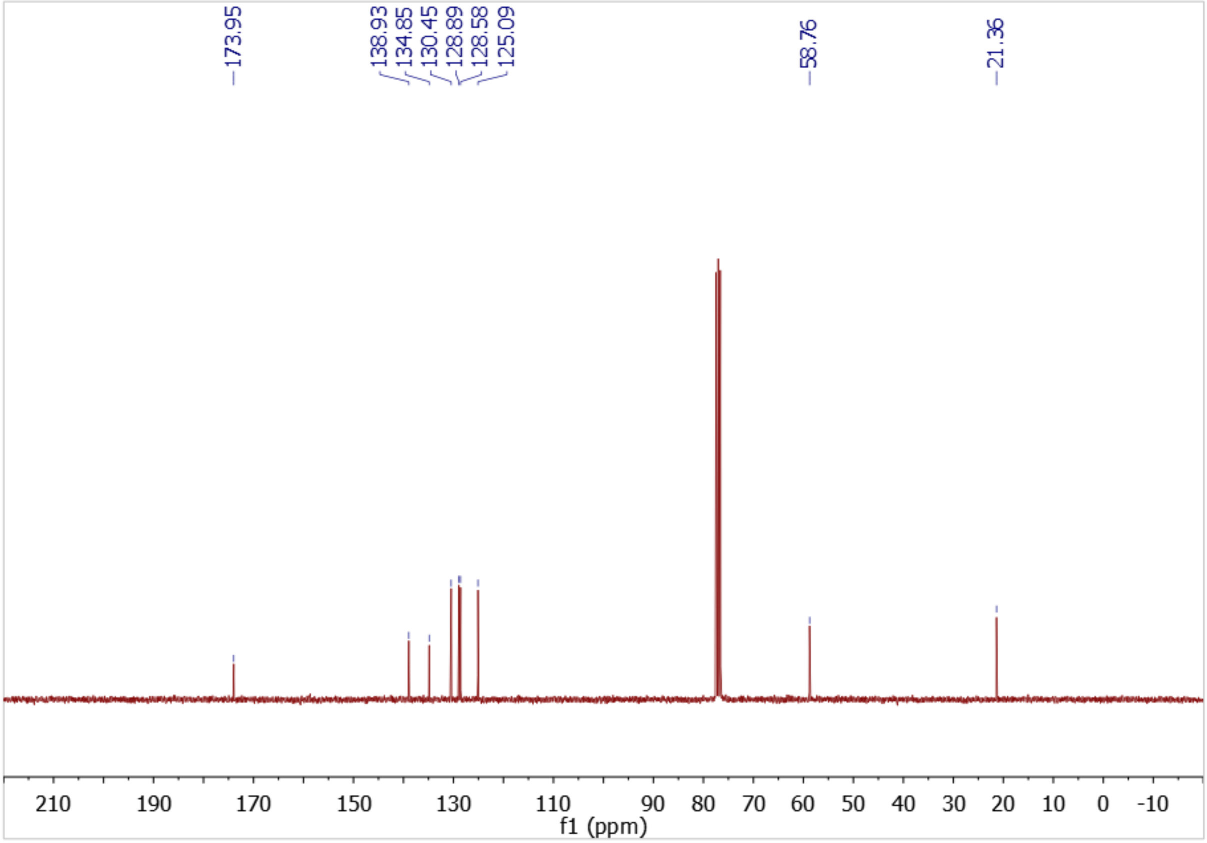
173.95
138.93 134.85 130.45 128.89 128.58 125.09
58.76
21.36
210 190 170 150 130 110 90 80 70 60 50 40 30 20 10 0 -10
f1 (ppm)

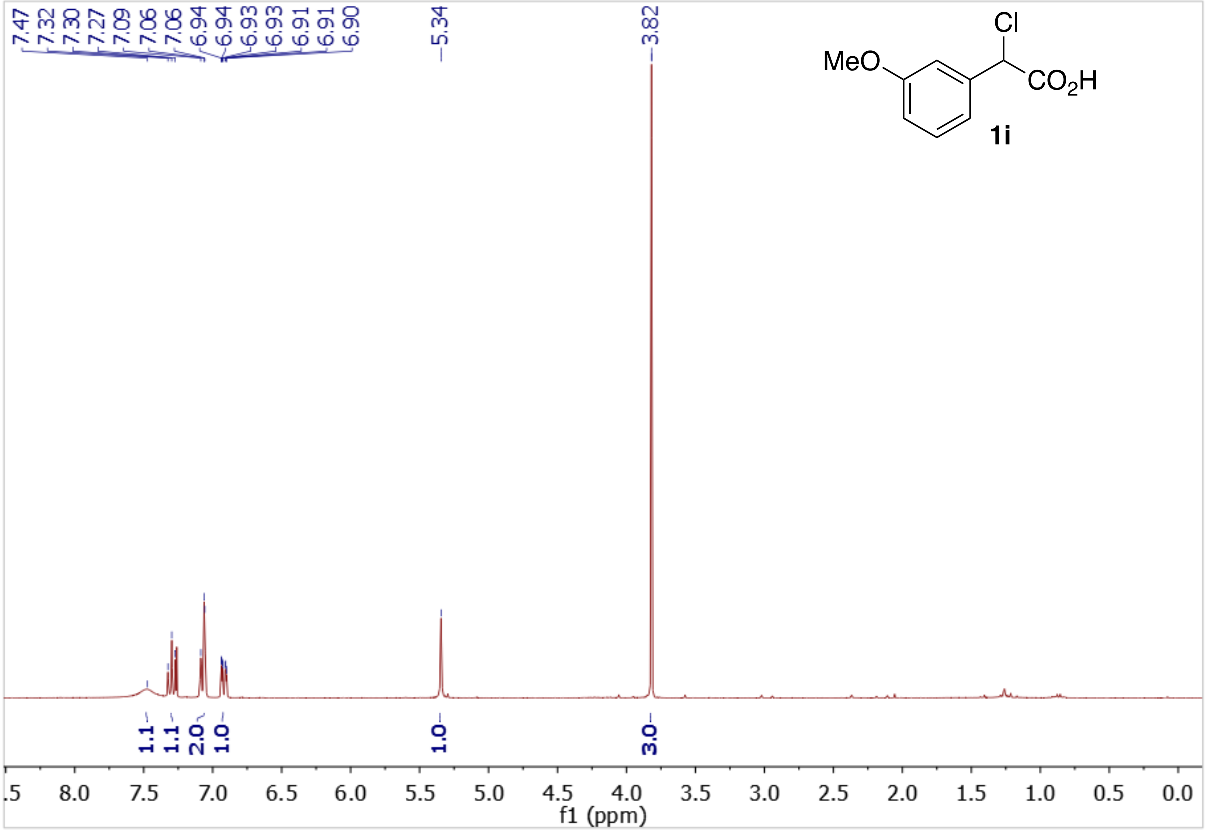
7.47
7.32
7.30
7.27
7.09
7.06
7.06
6.94
6.94
6.93
6.93
6.91
6.91
6.90
5.34
3.82
MeO
Cl
CO2H
1i
1.1
1.1
2.0
1.0
1.0
3.0
f1 (ppm)

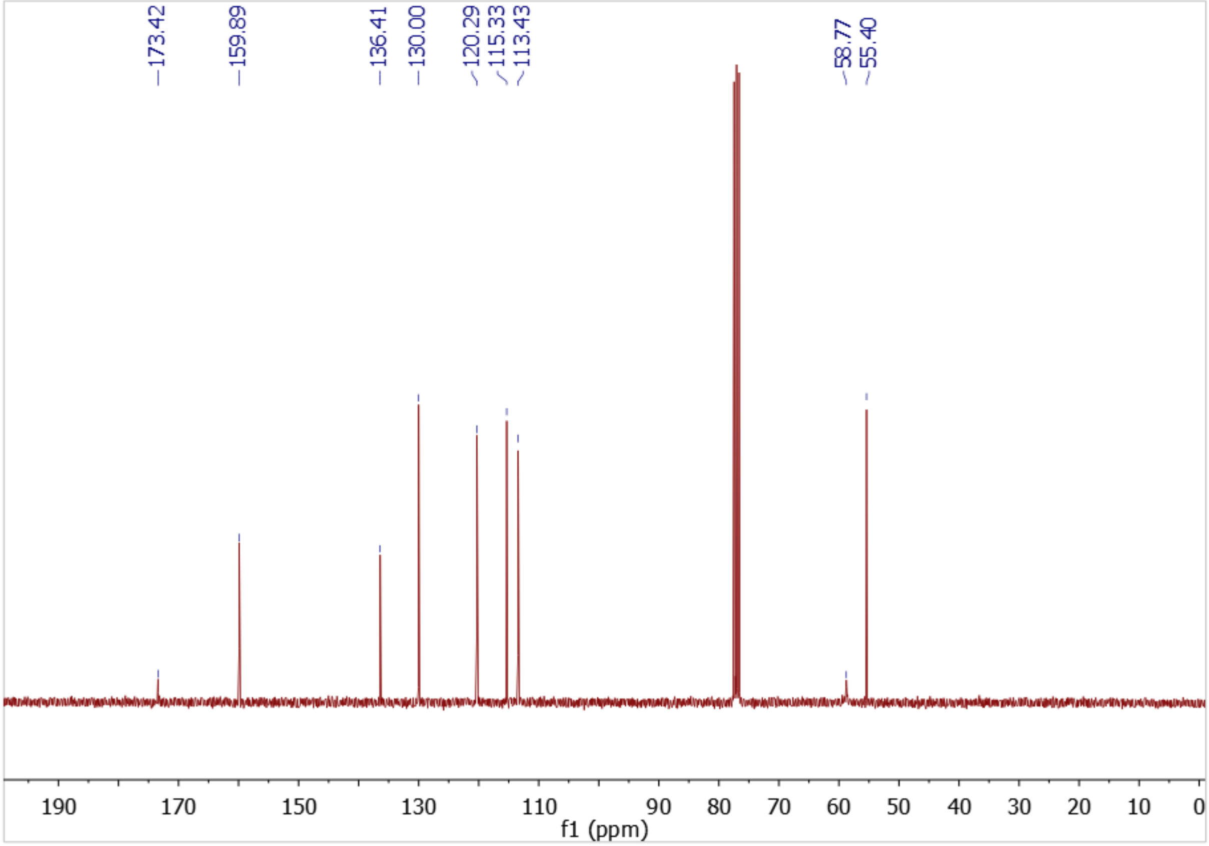
173.42
159.89
136.41
130.00
120.29
115.33
113.43
58.77
55.40
f1 (ppm)

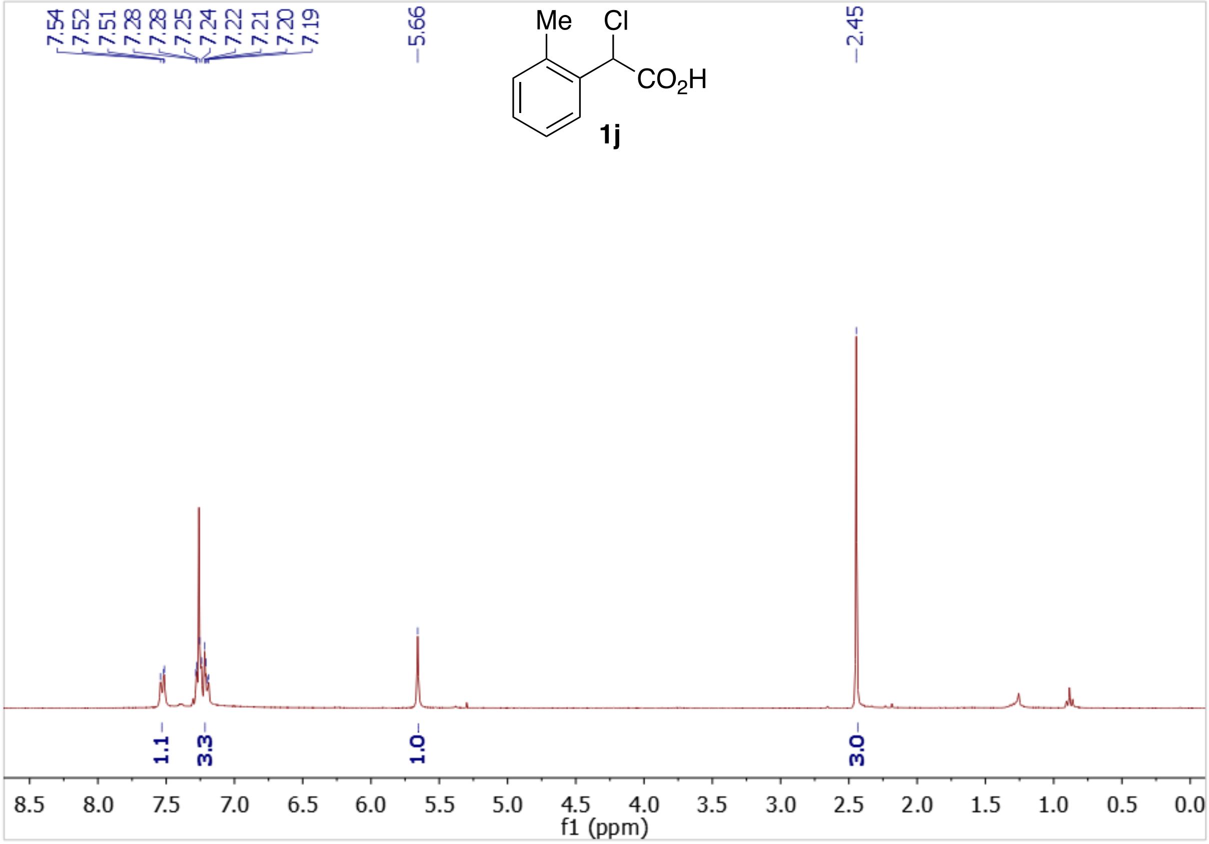
7.54 7.52 7.51 7.28 7.28 7.25 7.24 7.22 7.21 7.20 7.19
5.66
2.45
Me
Cl
CO_2H
1j
1.1
3.3
1.0
3.0
8.5 8.0 7.5 7.0 6.5 6.0 5.5 5.0 4.5 4.0 3.5 3.0 2.5 2.0 1.5 1.0 0.5 0.0
f1 (ppm)

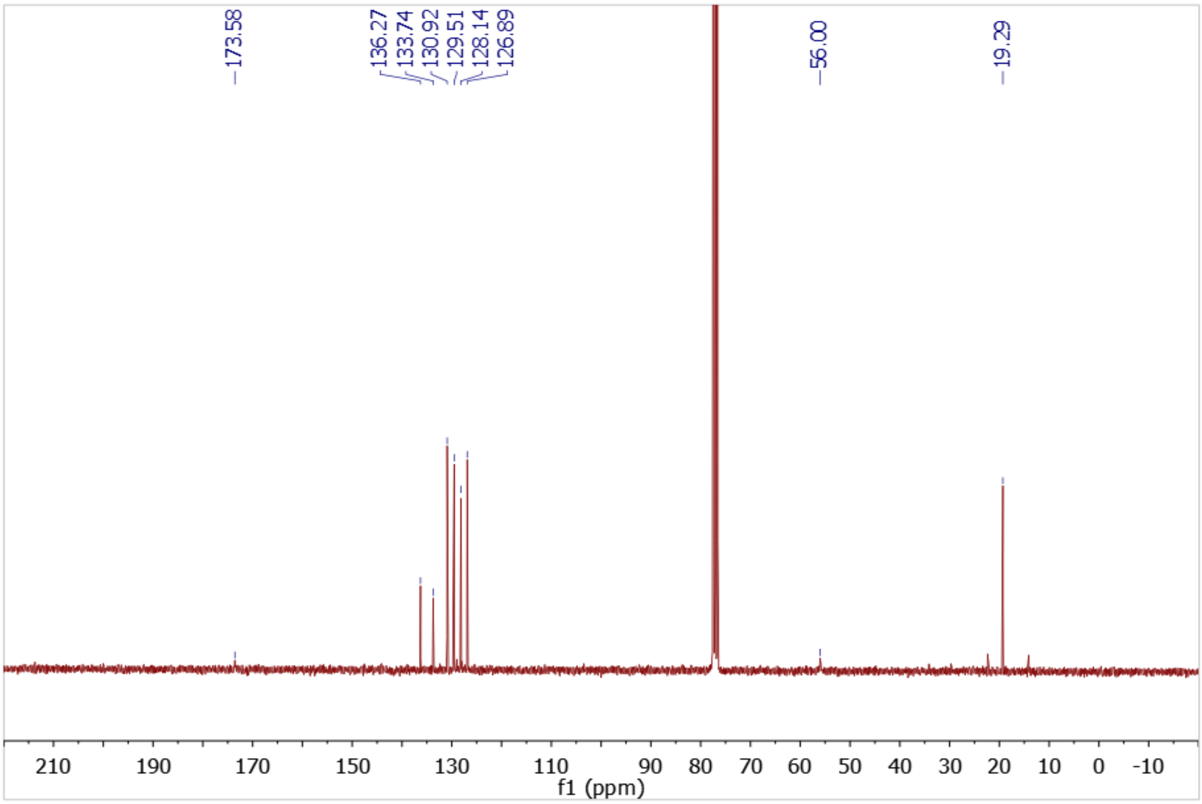
173.58
136.27 133.74 130.92 129.51 128.14 126.89
56.00
19.29
210 190 170 150 130 110 90 80 70 60 50 40 30 20 10 0 -10
f1 (ppm)

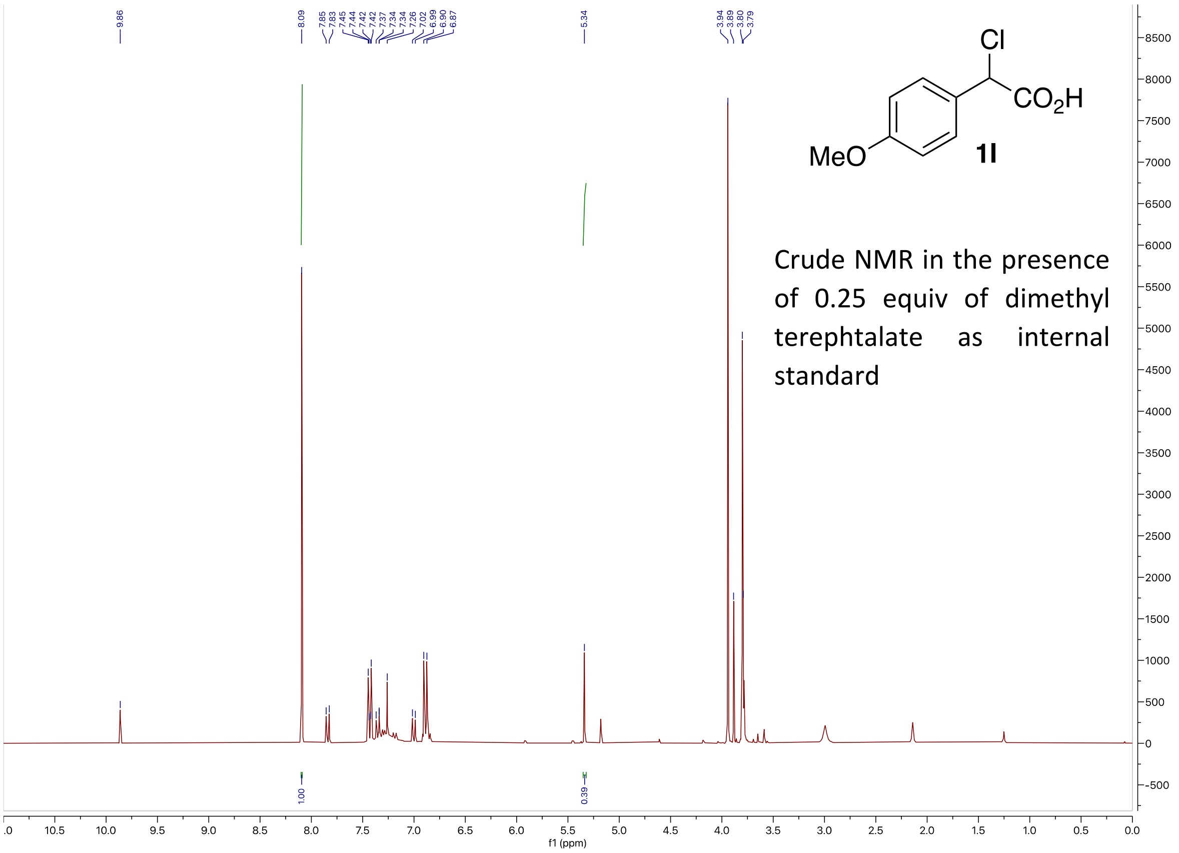
9.86
8.09
7.85
7.83
7.45
7.44
7.42
7.42
7.37
7.34
7.34
7.26
7.02
6.99
6.90
6.87
5.34
3.94
3.89
3.80
3.79
Cl
CO_2H
MeO
1l
Crude NMR in the presence of 0.25 equiv of dimethyl terephtalate as internal standard
1.00
0.39
f1 (ppm)

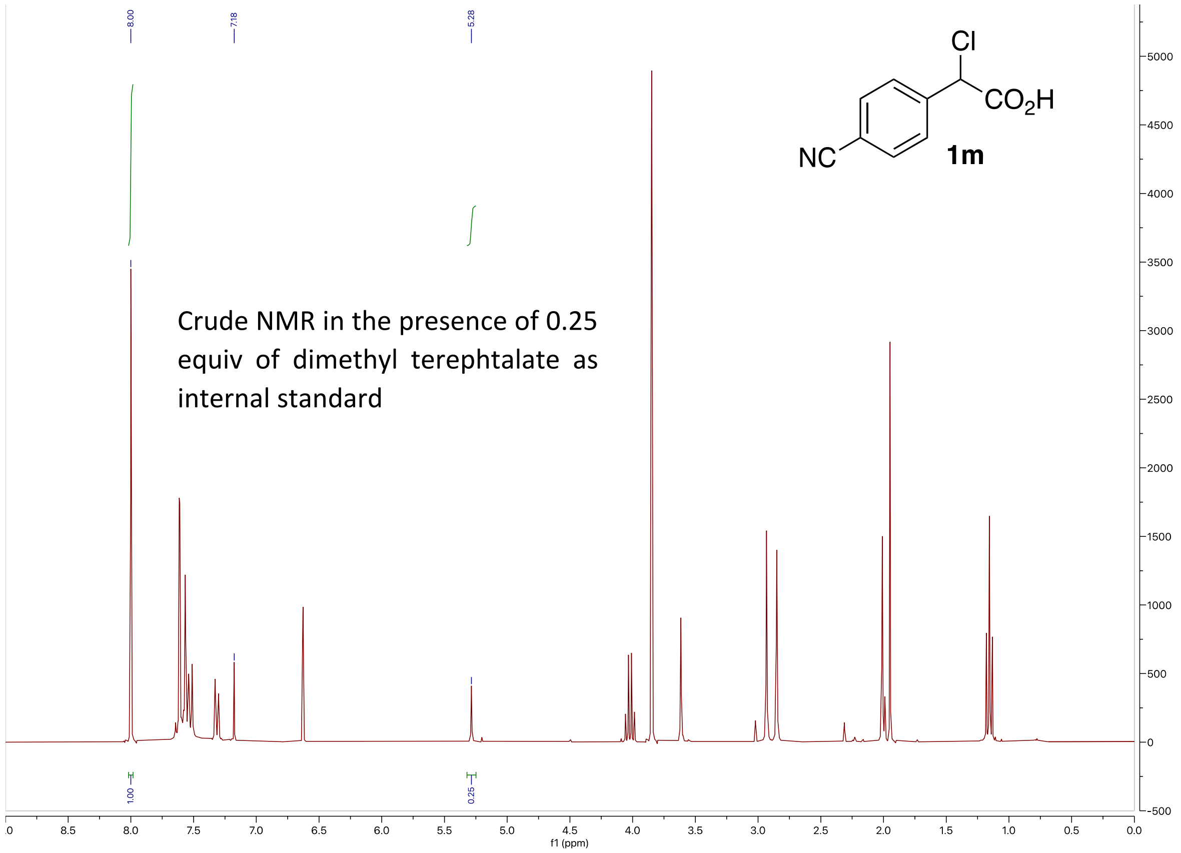
Cl
CO2H
NC
1m
Crude NMR in the presence of 0.25 equiv of dimethyl terephtalate as internal standard
f1 (ppm)

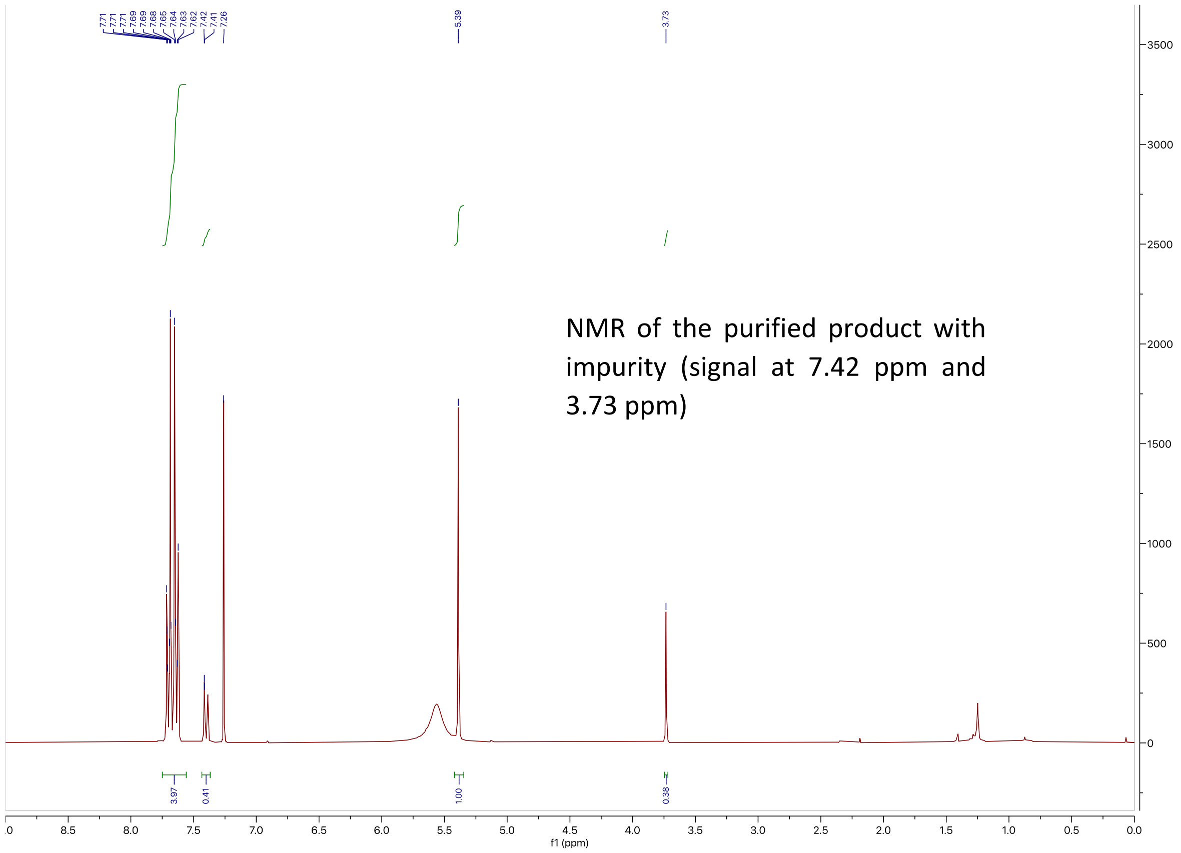
NMR of the purified product with impurity (signal at 7.42 ppm and 3.73 ppm)
f1 (ppm)

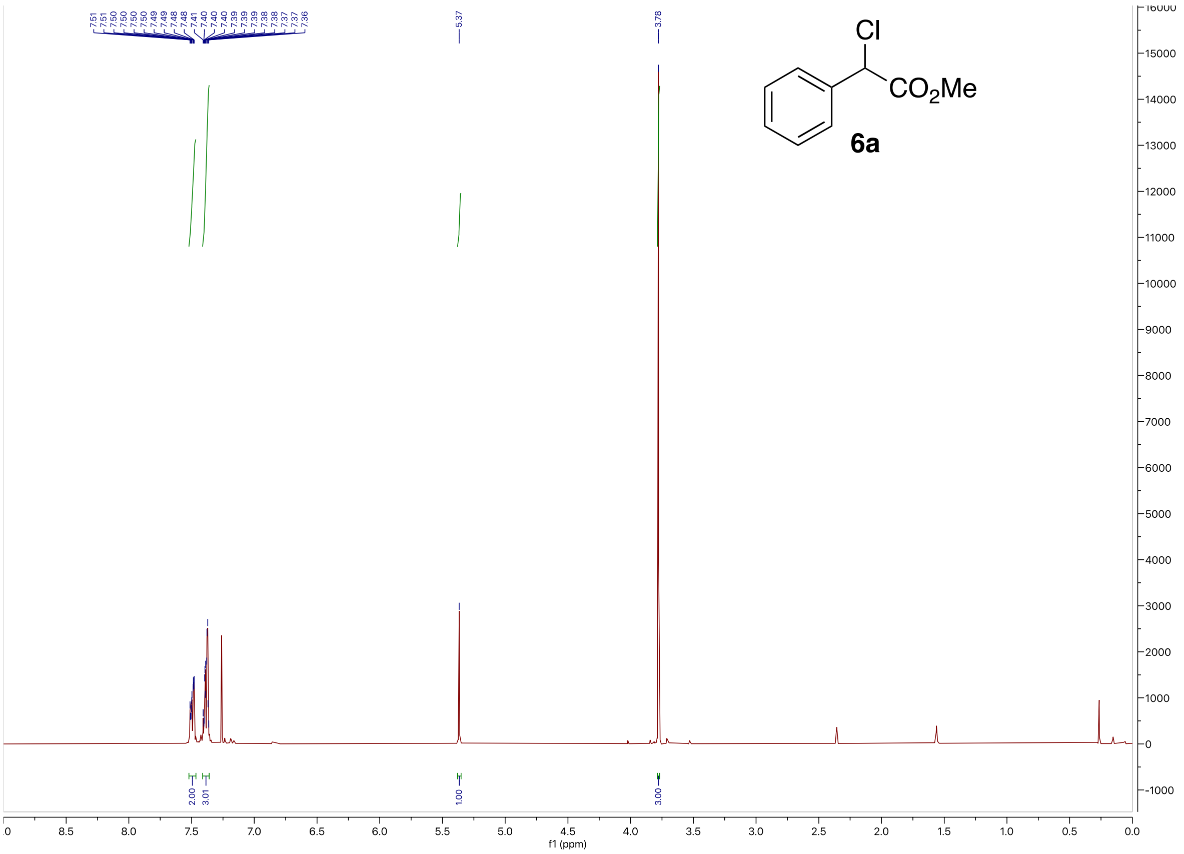
Cl
CO2Me
6a
f1 (ppm)

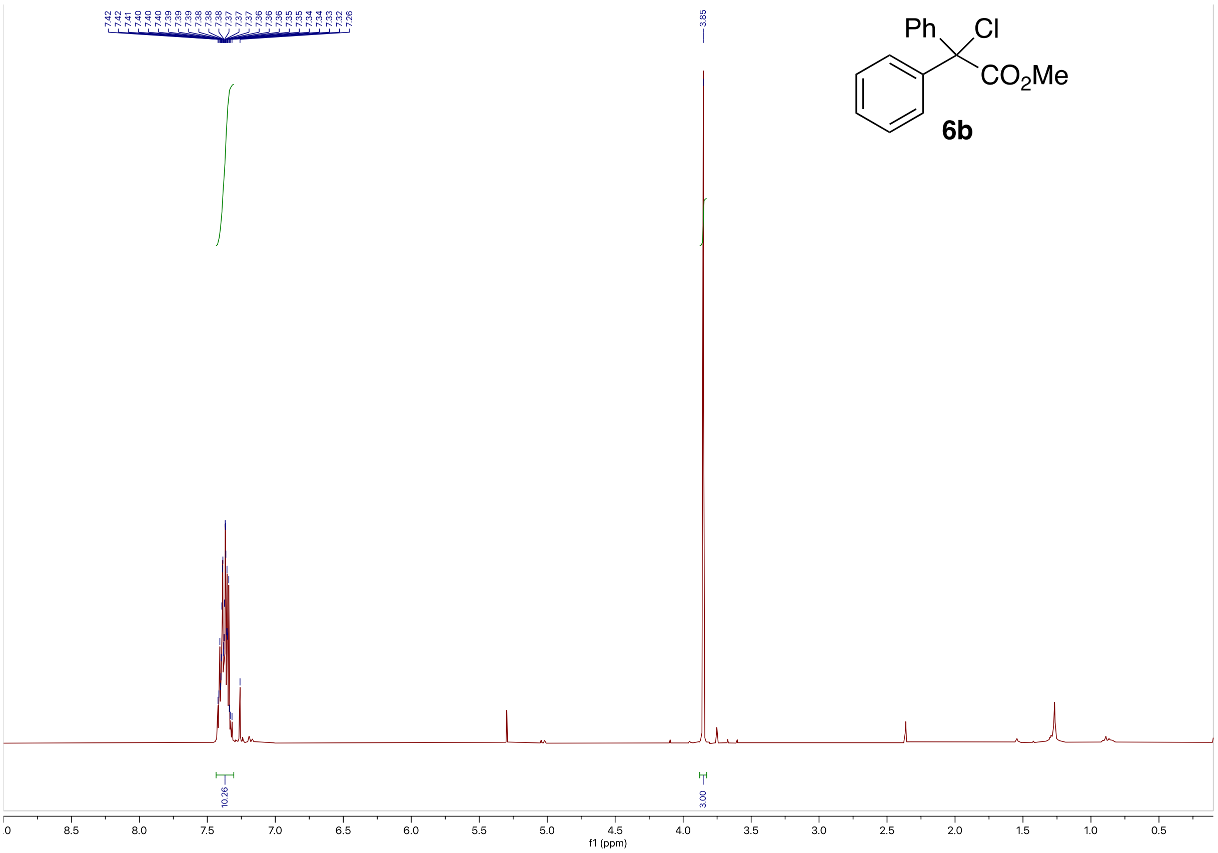
7.42
7.42
7.41
7.40
7.40
7.40
7.39
7.39
7.39
7.38
7.38
7.38
7.37
7.37
7.37
7.36
7.36
7.36
7.35
7.35
7.34
7.34
7.33
7.32
7.26
3.85
Ph
Cl
CO2Me
6b
10.26
3.00
9.0
8.5
8.0
7.5
7.0
6.5
6.0
5.5
5.0
4.5
4.0
3.5
3.0
2.5
2.0
1.5
1.0
0.5
f1 (ppm)

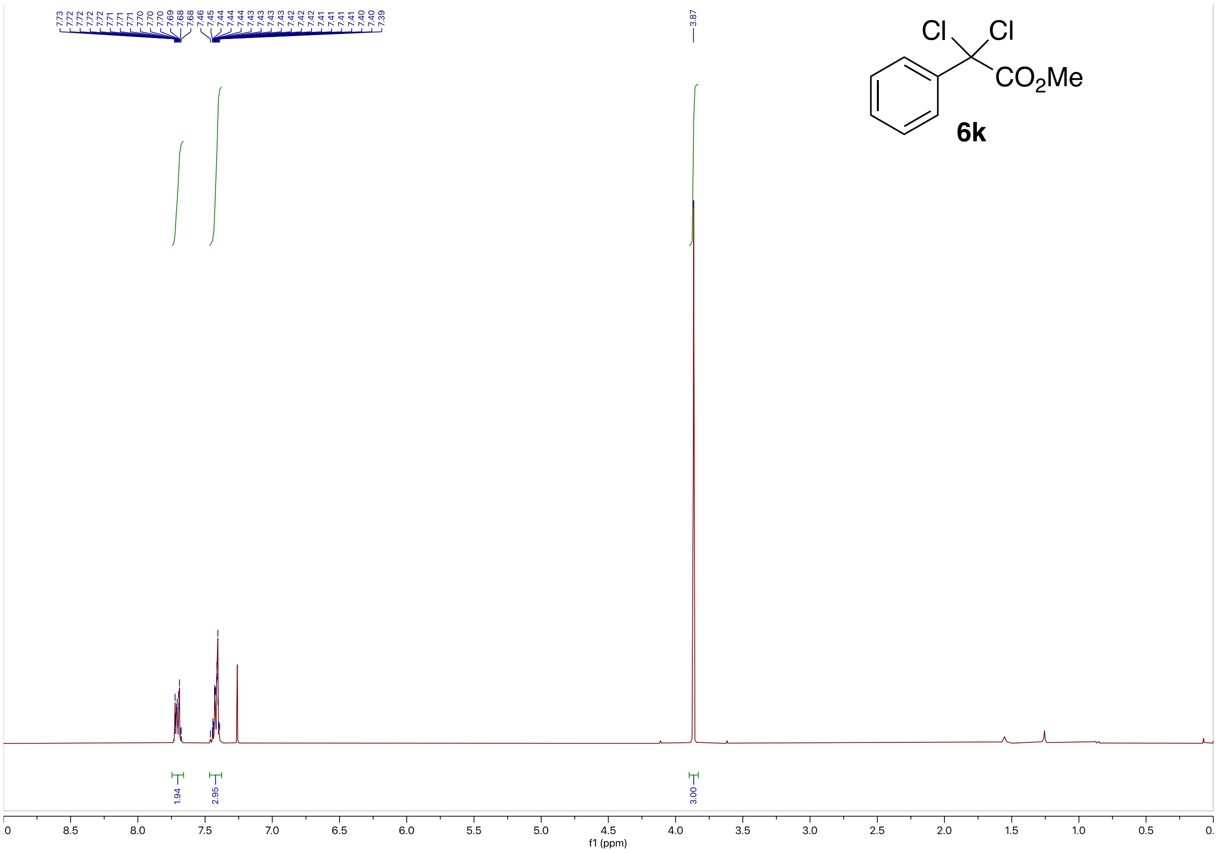

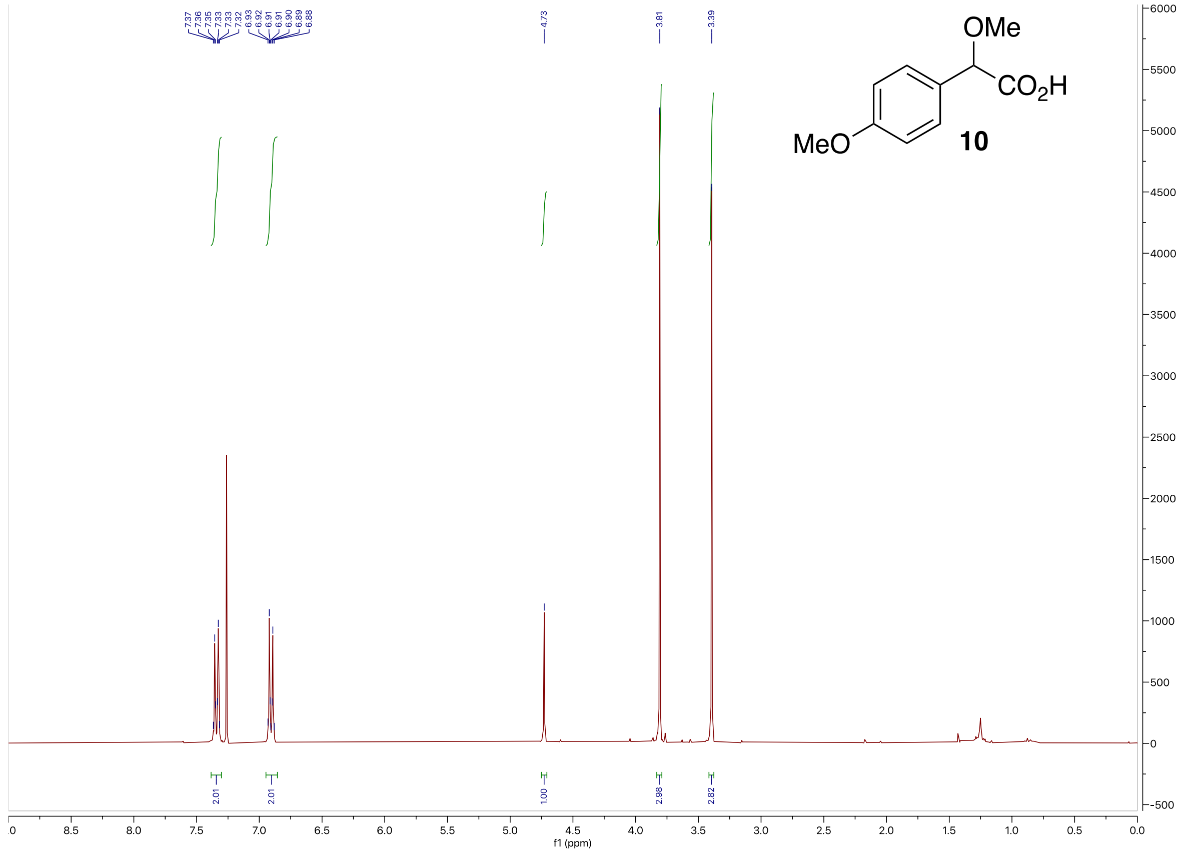
OMe
CO2H
MeO
10
f1 (ppm)

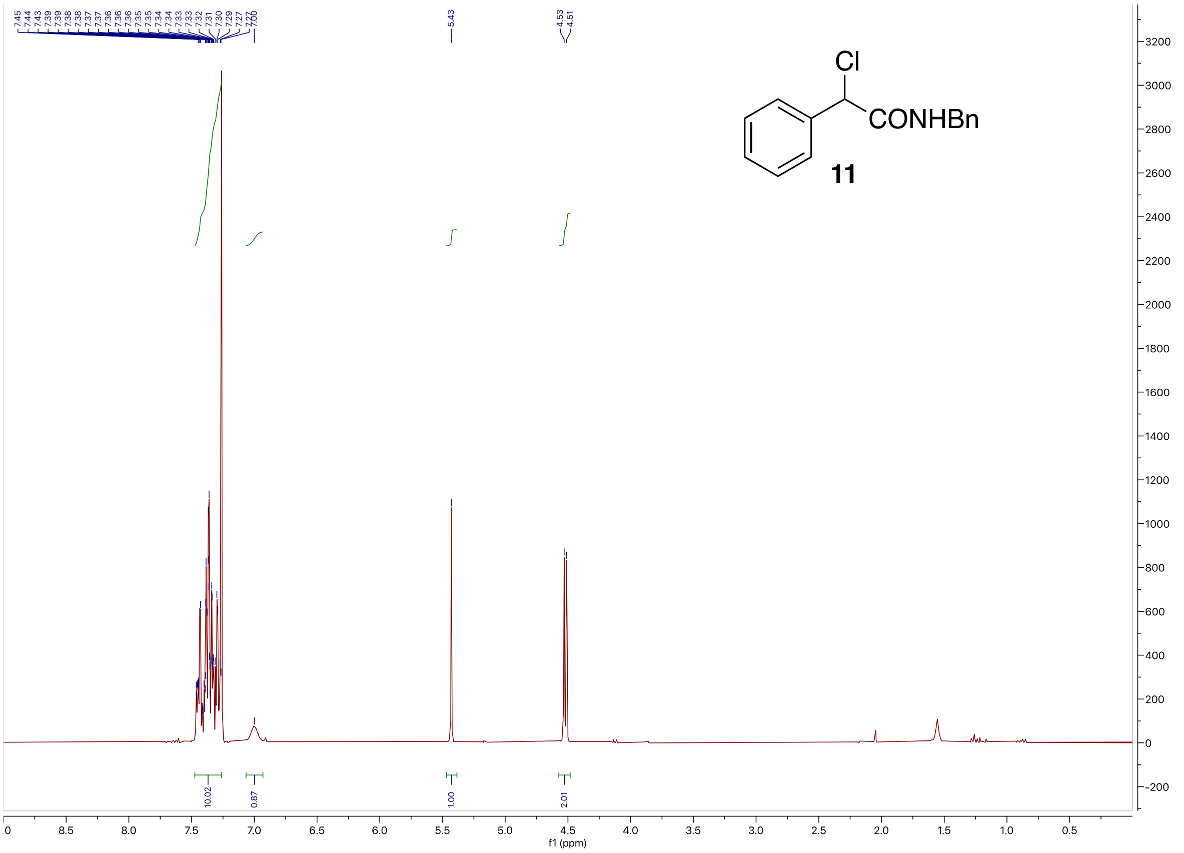
Cl
CONHBn
11
f1 (ppm)

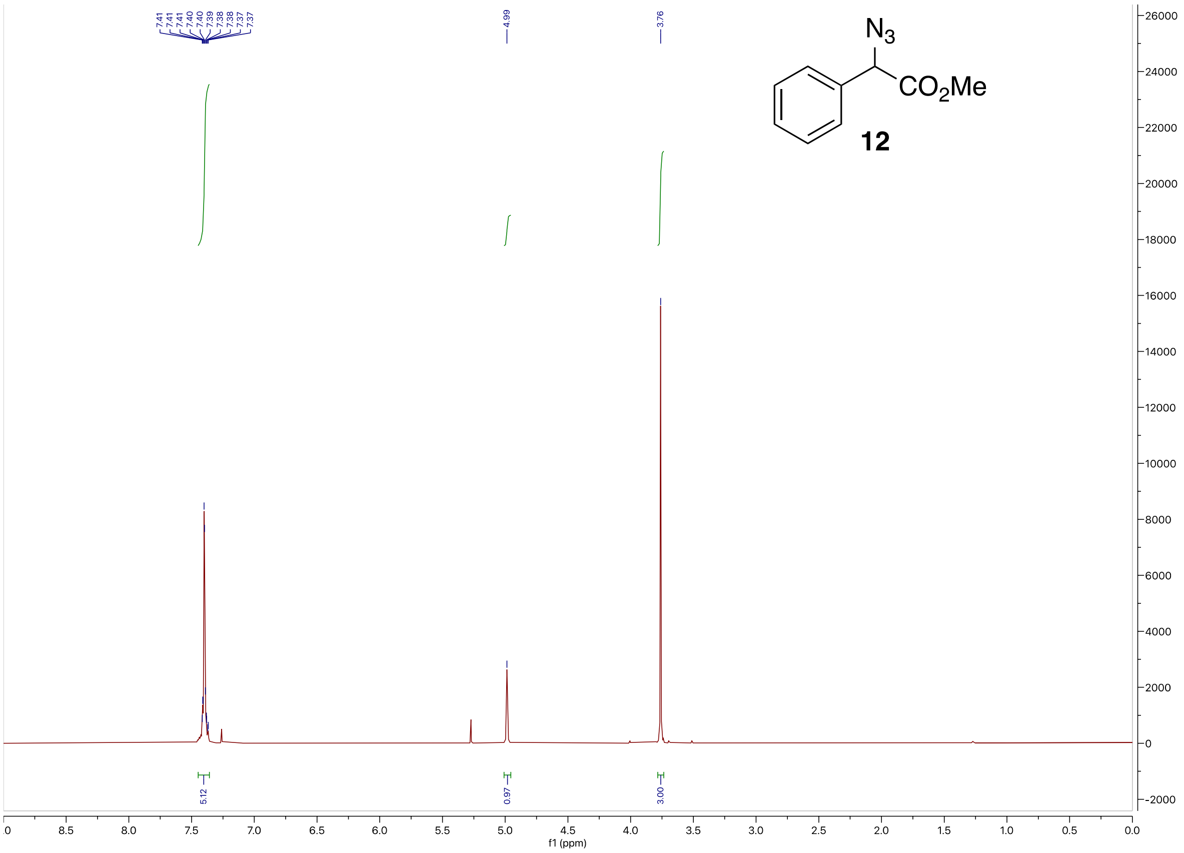

References

1. Fergus, S.; Eustace, S.J.; Hegarty, A.F. Nitrile ylide dimerization: Investigation of the carbene reactivity of nitrile ylides. *J. Org. Chem.* **2004**, *69*, 4663–4669. https://doi.org/10.1021/jo049748g
2. An, J.; Tang, X.; Moore, J.; Lewis, W.; Denton, R.M. Phosphorus(V)-catalyzed deoxydichlorination reactions of aldehydes. *Tetrahedron* **2013**, *69*, 8769–8776. https://doi.org/10.1016/j.tet.2013.07.100.
3. Léonel, E.; Paugam, J.-P.; Heintz, M.; Nédélec, J.-Y. A Simple and Efficient Procedure for the Preparation of Benzal Chlorides and Benzal Bromides. *Synth. Commun.* **1999**, *29*, 4015–4024.
4. Polat, E.; Cakici, M. Deoxygenative Chlorination of Aldehydes and Alcohols with Dichloromethyl Methyl Ether and $TiCl_4$. *Eur. J. Org. Chem.* **2022**, *2022*, e202201106.
5. Zhao, Z.; Kulkarni, K.G.; Murphy, G.K. Synthesis of Aryldihalomethanes by Denitrogenative Dihalogenation of Benzaldehyde Hydrazones. *Adv. Synth. Catal.* **2017**, *359*, 2222–2228.
6. Paraskevas, S.M.; Paraskevas, M.S. Chlorination and oxidation of some aldehydes by H_2O_2 and diphenic acid · $CuCl_2$ complex. *Catal. Commun.* **2004**, *5*, 687–690. https://doi.org/10.1016/j.catcom.2004.07.017.
7. Kelly, B.D.; Lambert, T.H. Aromatic cation activation of alcohols: Conversion to alkyl chlorides using dichlorodiphenylcyclopropene. *J. Am. Chem. Soc.* **2009**, *131*, 13930–13931. https://doi.org/10.1021/ja906520p
8. Timperley, C.M.; Bird, M.; Gore, S.J.; Lindsay, C.D.; Rice, H.; Tattersall, J.E.H.; Whitmore, C.L.; Green, A.C. 3-Quinuclidinyl-alpha-methoxydiphenylacetate: A multi-targeted ligand with antimuscarinic and antinicotinic effects designed for the treatment of anticholinesterase poisoning. *Toxicol. Lett.* **2020**, *325*, 67–76. https://doi.org/10.1016/j.toxlet.2020.01.027.
9. Tao, J.; Tran, R.; Murphy, G.K. Dihaloiodoarenes: α,α-dihalogenation of phenylacetate derivatives. *J. Am. Chem. Soc.* **2013**, *135*, 16312–16315.
10. Li, G.; Dong, H.; Ma, Y.; Shao, K.; Li, Y.; Wu, X.; Wang, S.; Shao, Y.; Zhao, W. Structure-activity relationships study of neolamellarin A and its analogues as hypoxia inducible factor-1 (HIF-1) inhibitors. *Bioorg. Med. Chem. Lett.* **2019**, *29*, 2327–2331. https://doi.org/10.1016/j.bmcl.2019.06.017.
11. Li, B.; Aliyu, M.A.; Gao, Z.; Li, T.; Dong, W.; Li, J.; Shi, E.; Tang, W. General Synthesis of Chiral alpha,alpha-Diaryl Carboxamides by Enantioselective Palladium-Catalyzed Cross-Coupling. *Org. Lett.* **2020**, *22*, 4974–4978. https://doi.org/10.1021/acs.orglett.0c01489.
12. Stockhammer, L.; Weinzierl, D.; Bogl, T.; Waser, M. Enantioselective alpha-Chlorination Reactions of in Situ Generated C1 Ammonium Enolates under Base-Free Conditions. *Org. Lett.* **2021**, *23*, 6143–6147. https://doi.org/10.1021/acs.orglett.1c02256.

13. Golas, P.L.; Tsarevsky, N.V.; Matyjaszewski, K. Structure–Reactivity Correlation in “Click” Chemistry: Substituent Effect on Azide Reactivity. *Macromol. Rapid Commun.* **2008**, *29*, 1167–1171. https://doi.org/10.1002/marc.200800118.
14. Munaretto, L.S.; Dos Santos, C.Y.; Gallo, R.D.C.; Okada, C.Y., Jr.; Deflon, V.M.; Jurberg, I.D. Visible-Light-Mediated Strategies to Assemble Alkyl 2-Carboxylate-2,3,3-Trisubstituted beta-Lactams and 5-Alkoxy-2,2,4-Trisubstituted Furan-3(2H)-ones Using Aryldiazoacetates and Aryldiazoketones. *Org. Lett.* **2021**, *23*, 9292–9296. https://doi.org/10.1021/acs.orglett.1c03662.
